# Extrachromosomal circular DNA expressing miRNA promotes ovarian cancer progression

**DOI:** 10.1002/ctm2.70445

**Published:** 2025-09-23

**Authors:** Ning Wu, Ling Wei, Qiyu Liu, Tianhui He, Cuiyu Huang, Yunpeng Jiang, Kailong Li, Hongyan Guo, Fengbiao Mao, Xiaolu Zhao

**Affiliations:** ^1^ State Key Laboratory of Female Fertility Promotion Center for Reproductive Medicine Department of Obstetrics and Gynecology Peking University Third Hospital Beijing China; ^2^ National Clinical Research Center for Obstetrics and Gynecology Peking University Third Hospital Beijing China; ^3^ Key Laboratory of Assisted Reproduction (Peking University) Ministry of Education Beijing China; ^4^ Beijing Key Laboratory of Reproductive Endocrinology and Assisted Reproductive Technology Beijing China; ^5^ Institute of Medical Innovation and Research Peking University Third Hospital Beijing China; ^6^ Cancer Center Peking University Third Hospital Beijing China; ^7^ Department of Biochemistry and Molecular Biology Beijing Key Laboratory of Protein Posttranslational Modifications and Cell Function School of Basic Medical Sciences Peking University Health Science Center Beijing China; ^8^ Beijing Key Laboratory for Interdisciplinary Research in Gastrointestinal Oncology (BLGO) Beijing China

**Keywords:** extrachromosomal circular DNA, miRNA, ovarian cancer

## Abstract

**Background:**

Extrachromosomal circular DNA (eccDNA) has emerged as a critical driver of oncogenesis, yet its functional roles in high‐grade serous ovarian cancer (HGSOC) remain poorly characterized. This highlights the need for comprehensive investigations into the abundance, biogenesis, and functional implications of eccDNA in HGSOC.

**Methods:**

To characterize eccDNA in HGSOC, we performed comprehensive Circle‐seq analysis to assess eccDNA abundance and genomic annotation in HGSOC tissues compared to normal ovarian tissue. For mechanistic validation of eccDNA biogenesis pathways, targeted knockdown experiments of microhomology‐mediated end‐joining (MMEJ) dependent on LIG3 and POLQ were conducted. Functional characterization of HGSOC‐specific eccDNA‐harboring precursor microRNAs (eccMIRs) included in vitro assays using HGSOC cells and in vivo tumor growth experiments.

**Results:**

Circle‐seq analysis revealed a 13‐fold increase in eccDNA abundance in HGSOC compared to normal ovarian tissue, with significant enrichment in promoter and coding regions. The MMEJ pathway was identified as the predominant pathway for eccDNA biogenesis in HGSOC, supported by characteristic microhomologies at junction sites and validation via LIG3 and POLQ knockdown experiments. Notably, HGSOC‐specific eccDNA frequently contained functional eccMIRs (eccMIR3661, eccMIR618, and eccMIR2277), which generate oncogenic miRNAs. These miRNAs promote tumor progression by downregulating tumor suppressor genes and activating key oncogenic pathways. Functional assays confirmed that these eccMIRs significantly enhanced HGSOC cell proliferation, migration, and invasion in vitro and promoted tumor growth in vivo.

**Conclusions:**

These results underscore eccDNA as an oncogenic driver in HGSOC through non‐coding RNA‐mediated regulatory mechanisms, revealing novel therapeutic opportunities for targeting eccDNA biogenesis in this aggressive malignancy.

**Key points:**

- This study revealed a 13‐fold increase of eccDNA in HGSOC compared to normal tissues, with significant enrichment in promoter and coding regions.
- eccDNA‐derived miRNAs (eccMIRs) were shown to enhance cancer cell proliferation, invasion, and tumor growth through the expression of oncogenic miRNA sequences.
- The study highlights the importance of the MMEJ pathway in eccDNA generation and proposes that targeting eccDNA biogenesis in this aggressive malignancy presents a novel therapeutic opportunity.

## Graphical abstract

1. This study revealed a 13‐fold increase in eccDNA in HGSOC compared to normal tissues, with significant enrichment in promoter and coding regions.
2. eccDNA‐derived miRNAs (eccMIRs) were shown to enhance cancer cell proliferation, invasion and tumour growth through the expression of oncogenic miRNA sequences.
3. The study highlights the importance of the MMEJ pathway in eccDNA generation and proposes that targeting eccDNA biogenesis in this aggressive malignancy presents a novel therapeutic opportunity.

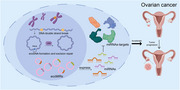

## 1 INTRODUCTION

Ovarian cancer (OC), a prevalent gynaecological malignancy originating primarily from ovarian tissues, represents a leading cause of female mortality.^1^, ^2^, ^3^ Among its histological subtypes, high‐grade serous ovarian cancer (HGSOC) is the most common and lethal variant, accounting for the majority of OC‐related deaths. Early detection of OC remains a major clinical challenge, as survival rates reach 90% for localised disease, but decline sharply in metastatic stages.^4^, ^5^ Peritoneal metastasis often occurs during the progression of OC, exacerbating patient prognosis, with a 5‐year survival rate of less than 30%.^6^ Cisplatin, a first‐line chemotherapeutic agent for OC, demonstrates an initial response rate of approximately 80%. However, the frequent development of acquired resistance often leads to disease relapse in advanced‐stage patients, ultimately resulting in treatment failure and increased mortality.^7^ The emergence of therapy resistance in OC poses a substantial obstacle to effective management. Given the significant burden of OC, particularly HGSOC, among gynecologic malignancies, elucidating its molecular mechanisms is urgently needed to improve diagnostic and therapeutic strategies.

eccDNA refers to circular DNA molecules that are derived from, but can exist independently of, a chromosome.^8^, ^9^ The prevalence of eccDNA is a widespread phenomenon observed in a range of eukaryotic organisms, including plants, ciliates, *Drosophila* and various mammalian species.^10^, ^11^ Extrachromosomal DNA (ecDNA), defined as eccDNA exceeding 1 Mb in size, has emerged as a critical oncogenic driver present in 17.1% of human tumours.^12^, ^13^, ^14^, ^15^ ecDNA offers an effective mechanism for oncogene amplification by directly increasing the copy number or indirectly upregulating transcription levels, serving as cis‐ or trans‐acting factors such as mobile super‐enhancers.^16^, ^17^, ^18^, ^19^ For example, researchers have shown that the copy number of ecDNA containing *EGFR* was identified as the primary driver of elevated *EGFR* transcription levels in *EGFR*‐containing glioblastoma (GBM)‐derived glioma stem cells.^20^ Moreover, the amplification of EGFR on ecDNA was frequently accompanied by the co‐amplification of its adjacent non‐coding region, facilitating the formation of novel enhancer–oncogene interactions that drove tumourigenesis.^21^ ecDNA was also shown to exhibit increased chromatin accessibility compared to the corresponding genomic oncogene, endowing ecDNA with elevated transcriptional activity in both colon cancer and glioblastoma.^13^ Furthermore, ecDNA could promote cancer evolution through copy number heterogeneity as well as genetic and epigenetic heterogeneity.^13^, ^14^, ^15^, ^19^, ^21^, ^22^, ^23^, ^24^, ^25^ In a recent study, Hung et al. demonstrated that different kinds of ecDNA could co‐occur in cancer cells and co‐segregate during mitosis. These evolutionary connections play a role in shaping ecDNA specialisation and influencing the response to targeted therapy.^26^ Therefore, ecDNA holds significant potential to serve as a therapeutic target for preventing the initiation and progression of tumours, as well as serving as a valuable biomarker for tumour diagnosis and prognostic prediction.^27^ For instance, Tang et al. revealed that the CHK1 inhibitor BBI‐2779 could enhance ecDNA damage during DNA replication, leading to the specific elimination of cancer cells containing ecDNA.^28^ The aforementioned studies suggested a close association between ecDNA and the initiation and progression of cancer. Current research on eccDNA in cancer has primarily focused on large ecDNA (>1 Mb) containing protein‐coding genes and enhancers, while the functional significance of small eccDNA (<100 kb) carrying non‐coding RNAs remains largely unexplored. eccDNA has been recognised as a potential source of functional RNAs, including microRNAs (miRNAs), small interfering (si)‐like RNAs, and short hairpin (sh)‐like RNAs.^29^, ^30^, ^31^, ^32^ Researchers found that small artificial eccDNA inhibits gene expression by producing short regulatory RNA in HCT116, HEK293A and HEK293T mammalian cells.^30^ Nevertheless, the functions of eccDNA containing miRNAs in ovarian cancer have not been thoroughly explored.

In this study, we employed Circle‐seq to investigate the landscape of eccDNA in HGSOC and confirmed the presence of eccDNA carrying carcinogenic pre‐miRNA (eccMIR) in HGSOC tissues. Moreover, we found that miRNA genes on these eccDNA upregulated the expression of their corresponding miRNAs, which in turn downregulated the transcription of associated tumour suppressor genes. Functional validation showed that HGSOC‐overexpressed eccMIRs enhance tumourigenicity by promoting cell proliferation and invasion via oncogenic miRNAs, which was further confirmed by accelerated tumour growth in eccMIR‐overexpressing xenografts. In addition, we revealed that MMEJ mediated by LIG3 and POLQ plays a key role in eccDNA generation in HGSOC. Our findings elucidate the landscape of eccDNA in HGSOC and highlight the functional importance of miRNA‐carrying eccDNAs as potential drivers of tumour pathogenesis.^33^ This study advances the molecular understanding of HGSOC development and progression, providing critical insights for future research and therapeutic strategies targeting eccDNA‐mediated oncogenic mechanisms.

## 2 RESULTS

### 2.1 The landscape of eccDNA in HGSOC

**FIGURE 1:**
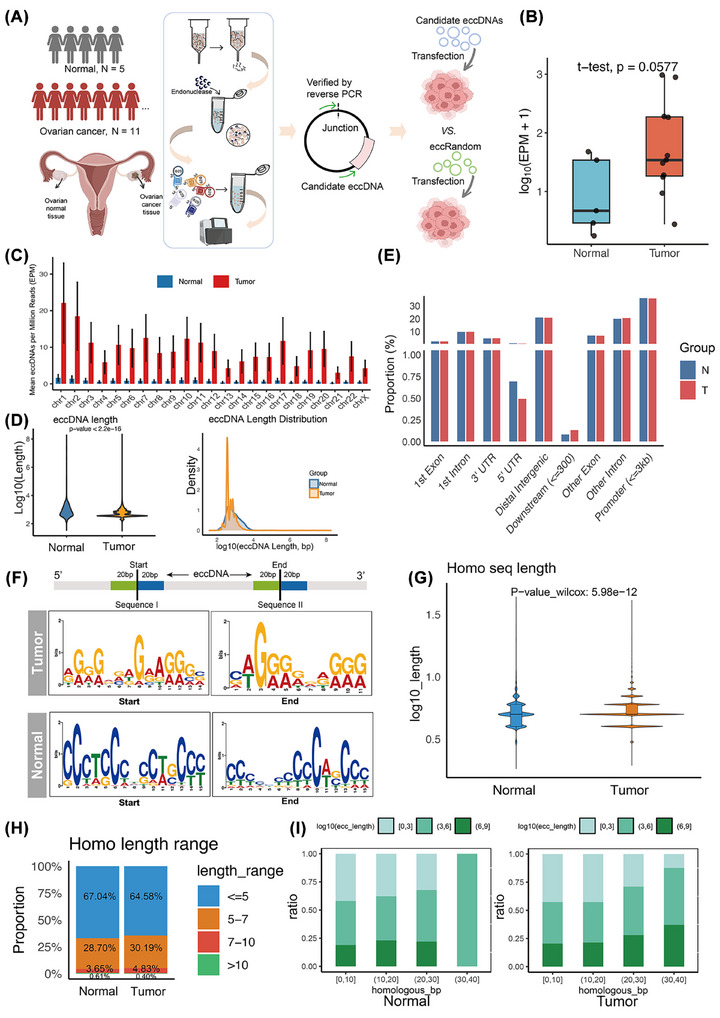
Basic characteristics of eccDNA detected in ovarian cancer (OC) and normal ovarian tissues. (A) The experimental workflow, including sample collection, Circle‐seq‐based sequencing and eccDNA validation. (B) Comparison of eccDNA abundance between OC and normal ovarian tissues. The statistical test employed was the *t*‐test. The average eccDNA counts were 37 556 (OC) and 2 888 (normal). *y*‐Axis: eccDNA abundance [log10(EPM + 1)]; *x*‐axis: experimental groups. (C) The abundance of eccDNA in 23 chromosomes of OC and normal ovarian tissues. (D) Length distribution of eccDNA in OC versus normal ovarian tissues. Median eccDNA sizes: 47 953 bp (OC) and 156 235 bp (normal). (E) Distribution of eccDNA in the indicated genomic region between OC and normal ovarian tissues. In tumour group: promoters (≤3 kb; 153 952, 35.62%), introns (129 385, 29.94%), and exons (37 442, 8.66%), downstream regions (≤300 bp; 582, .13%), 5′ UTRs (2 193, .49%), distal intergenic (88 602, 20.5%), and 3′ UTR (20 140, 4.66%). (F) Schematic description of the 20 bp upstream and downstream sequences flanking the 5′ and 3′ junction sites of eccDNA (the homologous sequences for the following cases G, H and I were all obtained in this manner), and motifs enriched on both sides of eccDNA junction sites in tumour tissues and normal tissues. (G) A comparison of homologous sequence lengths flanking eccDNA junctions between OC tissues and normal ovarian tissues. (H) Distribution of homology length ranges flanking eccDNA junctions in OC and normal ovarian tissues. (I) The relationship between the lengths of homologous sequences flanking eccDNA junction sites and the lengths of eccDNA in OC tissues and normal tissues.

To comprehensively identify eccDNA in HGSOC and normal ovarian tissue at a genomic scale, we conducted Circle‐seq analysis on 11 HGSOC tissues and five normal tissues (Figure 1A).^34^ A total of 447 562 eccDNA were detected, with HGSOC samples showing an average eccDNA count approximately 13 times higher than that in normal samples, reflecting the increased genomic instability characteristic of HGSOC tumours (Figure 1B and Figure S1B).^35^, ^36^, ^37^ HGSOC tissues exhibited significantly greater and more focal eccDNA distribution across chromosomes, supporting their pathogenic contribution through potential oncogene amplification (Figure 1C). The size distribution of eccDNA ranged from 100 bp to 100 Mb, with the majority falling between 100 bp and 10 kb (Figure S1C). Interestingly, eccDNA in HGSOC samples tended to be shorter compared to normal samples (median size: 47 953 bp vs. 156 235 bp), suggesting that heightened DNA fragmentation in HGSOC may promote eccDNA biogenesis (Figure 1D).^38^ Genomic region analysis revealed that eccDNAs were notably enriched in promoter and gene‐coding regions in both normal and tumour tissues. In HGSOC, eccDNAs showed particularly high enrichment in promoters (≤3 kb; 153 952, 35.62%), introns (129 385, 29.94%), and exons (37 442, 8.66%), while exhibiting minimal enrichment in downstream regions (≤300 bp; 582, .13%) and 5′UTRs (2 193, .49%). Normal tissues displayed a similar distribution pattern but with lower overall counts (Figure 1E and Figure S2A). This distribution pattern supports the potential involvement of eccDNAs in regulating gene expression processes in HGSOC. The differences in length and quantity of eccDNA between cancerous and normal tissues indicate a significant involvement of eccDNA in the initiation and progression of HGSOC.

### 2.2 Microhomologous recombination is required for eccDNA generation

The formation of eccDNA in cells is closely associated with endogenous DNA repair mechanisms.^39^ Notably, the presence of direct repeats flanking eccDNA junction sites has been proposed as a potential driver of its biogenesis.^40^ The landscape of eccDNA revealed in this study indicates that eccDNA exhibits a higher degree of fragmentation in HGSOC, suggesting a potential link to DNA damage repair pathways. To investigate the formation of eccDNA, we conducted a comprehensive analysis of the motif sequences within the 20 bp regions flanking both the start and end positions (junction site) of all detected eccDNA. The motif sequence observed at the junction site in the eccDNA from both tumours and normal tissues indicates a preference for guanine (G) and cytosine (C) bases (Figure 1F). While homologous sequences in both normal and tumour groups are predominantly concentrated within a range of less than 5 bp, the cancer group exhibits a higher incidence of microhomologous recombination involving longer homologous sequences compared to normal tissues (Figure 1G,H). We observed that eccDNA size positively correlated with homologous sequence length at junction sites (Figure 1I), where shorter homologous sequences corresponded to smaller eccDNAs and longer sequences to larger eccDNAs. This size‐dependent pattern indicates distinct eccDNA biogenesis mechanisms: smaller eccDNAs in tumours likely arise through microhomology‐mediated end joining (MMEJ), while larger eccDNAs may be generated via homologous recombination (HR) or non‐homologous end joining (NHEJ).

**FIGURE 2:**
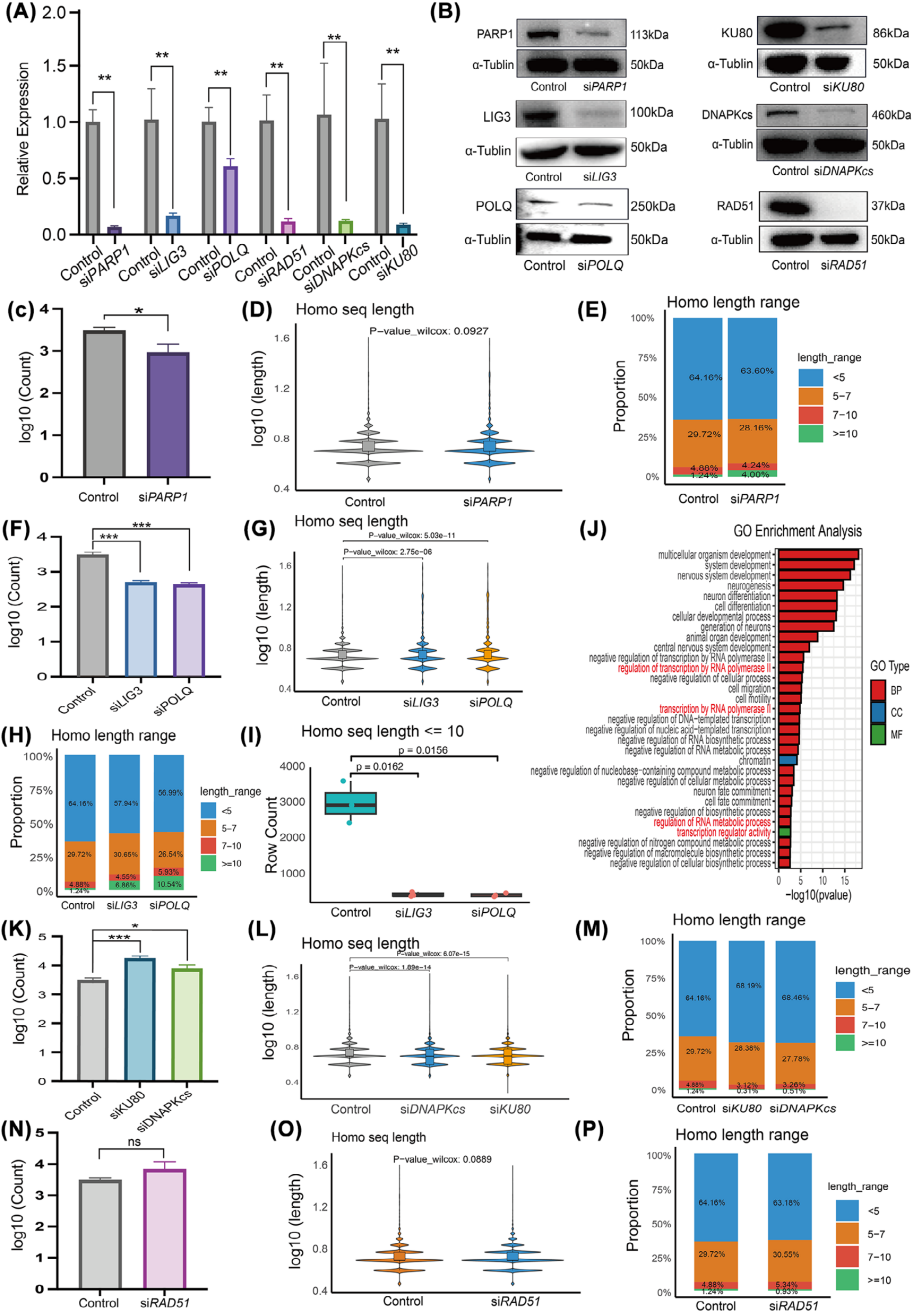
eccDNA formation mechanisms in ovarian cancer (OC) cells. (A and B) Validation of the knockdown efficiency of *PARP1*, *LIG3, POLQ, DNA‐PKcs, KU80* and *RAD51* at the mRNA level (A) and protein level (B), respectively. (C) Comparison of eccDNA abundance in wild‐type (WT) and si*PARP1* SKOV3 cells. *y*‐Axis: eccDNA abundance [log10(count)]; *x*‐axis: experimental groups. (D) Comparison of homologous sequence length at eccDNA junction sites in WT and si*PARP1* SKOV3 cells. *y*‐Axis: eccDNA length (log10(length)); *x*‐axis: experimental groups. (E) Proportion of eccDNA with various homologous sequence sizes in WT and si*PARP1* SKOV3 cells. (F) Comparison of eccDNA abundance in WT, si*LIG3* and si*POLQ* SKOV3 cells, respectively. *y*‐Axis: eccDNA abundance [log10(count)]; *x*‐axis: experimental groups. (G) Comparison of homologous sequence length at eccDNA junction sites in WT, si*LIG3* and si*POLQ* SKOV3 cells. *y*‐Axis: eccDNA length [log10(length)]; *x*‐axis: experimental groups. (H) Proportion of eccDNA with various homologous sequence sizes in WT, si*LIG3* and si*POLQ* SKOV3 cells. (I) Comparison of eccDNA count with microhomology (≤10 bp) in WT, si*LIG3* and si*POLQ* SKOV3 cells. *y*‐Axis: eccDNA count [log10(count)]; *x*‐axis: experimental groups. (J) Pathways enriched for downregulated eccDNA‐associated genes in si*LIG3* SKOV3 cells. (K) Comparison of eccDNA abundance in WT, si*KU80* and si*DNA‐PKcs* SKOV3 cells, respectively. *y*‐Axis: eccDNA abundance [log10(count)); *x*‐axis: experimental groups. (L) Comparison of homologous sequence length at eccDNA junction sites in WT, si*KU80* and si*DNA‐PKcs* SKOV3 cells. *y*‐Axis: eccDNA length [log10(length)]; *x*‐axis: experimental groups. (M) Proportion of eccDNA with various homologous sequence sizes in WT, si*KU80* and si*DNA‐PKcs* SKOV3 cells. *y*‐Axis: eccDNA length [log10(length)]; *x*‐axis: experimental groups. (N) Comparison of eccDNA abundance in WT and si*RAD51* SKOV3 cells. *y*‐Axis: eccDNA abundance [log10(count)]; *x*‐axis: experimental groups. (O) Comparison of homologous sequence length at eccDNA junction sites in WT and si*RAD51* SKOV3 cells. *y*‐Axis: eccDNA length [log10(length)]; *x*‐axis: experimental groups. (P) Proportion of eccDNA with various homologous sequence sizes in WT and si*RAD51* SKOV3 cells. Data are presented as mean ± SE of three or four independent experiments. Significance was determined by Student's *t*‐test: ns, not significant; *p < .05, **p < .01, ***p < .001 and ****p < .0001.

To delineate the contributions of DSB repair pathways (MMEJ, NHEJ and HR) to eccDNA biogenesis, we systematically knocked down key pathway‐specific factors in SKOV3 ovarian cancer cells: *LIG3* and *POLQ* for MMEJ, *KU80* and *DNA‐PKcs* for NHEJ, *RAD51* for HR, and *PARP1* as an upstream regulator of DNA repair (Figure 2A,B). Depletion of PARP1 led to a marked decrease in eccDNA abundance, demonstrating the essential requirement of DSB repair pathways for eccDNA biogenesis (Figure 2C–E). Knockdown of either *LIG3* or *POLQ*, two key MMEJ factors, significantly reduced eccDNA production (*LIG3*: *p *= 4e−04; *POLQ*: *p *= 2e−04) (Figure 2F), with a pronounced effect on eccDNAs containing less than 10 bp homologous sequences (Figure 2G–I), further supporting MMEJ's role in eccDNA biogenesis.^33^ Functional enrichment analysis revealed that *LIG3* knockdown downregulated pathways associated with its known functions, including RNA polymerase II‐mediated transcription and RNA metabolic processes (Figure 2J). It suggests that the disruption of the MMEJ DNA repair pathway could lead to disruptions in the transcription process, subsequently affecting downstream transcriptional regulatory pathways.

Knockdown of NHEJ factors (*KU80*/*DNA‐PKcs*) and HR factors (*RAD51*) yielded opposing phenotypes in eccDNA formation. Mechanistically, NHEJ mediates direct ligation of broken DNA ends without requiring homologous templates,^41^ whereas HR promotes genomic stability through homology‐directed repair via strand exchange and recombination. Knockdown of NHEJ components (*KU80*/*DNA‐PKcs*) significantly increased eccDNA abundance (Figure 2K), while reducing both global junctional homology length (Figure 2L) and the proportion of eccDNAs with longer homologous sequences (Figure 2M). *RAD51* knockdown showed a modest, non‐significant trend toward increased eccDNA accumulation (Figure 2N), but did not affect homologous sequence length at junction sites (Figure 2O). However, RAD51 depletion did reduce eccDNAs containing longer homologous sequences (Figure 2P), supporting previous evidence that HR generates large eccDNAs through long homologous junctions.^42^ Impairment of these two canonical repair pathways may induce genomic instability while promoting aberrant reliance on alternative mechanisms like MMEJ.^40^, ^43^ Consequently, this perturbation will not reduce but may even increase the abundance of eccDNA. To further investigate the crosstalk between these DNA repair pathways, we assessed LIG3 expression following *RAD51* knockdown using RT‐qPCR and Western blot analysis. While we observed a trend of LIG3 upregulation in some experiments (Figure S2E), the effect was not consistently reproducible across replicates. It suggests that RAD51 depletion may partially or indirectly modulate MMEJ‐related gene expression, but the regulatory mechanism appears to be context‐dependent or involves additional factors requiring further investigation.

### 2.3 Differentially expressed eccDNA‐harbouring genes in HGSOC

**FIGURE 3:**
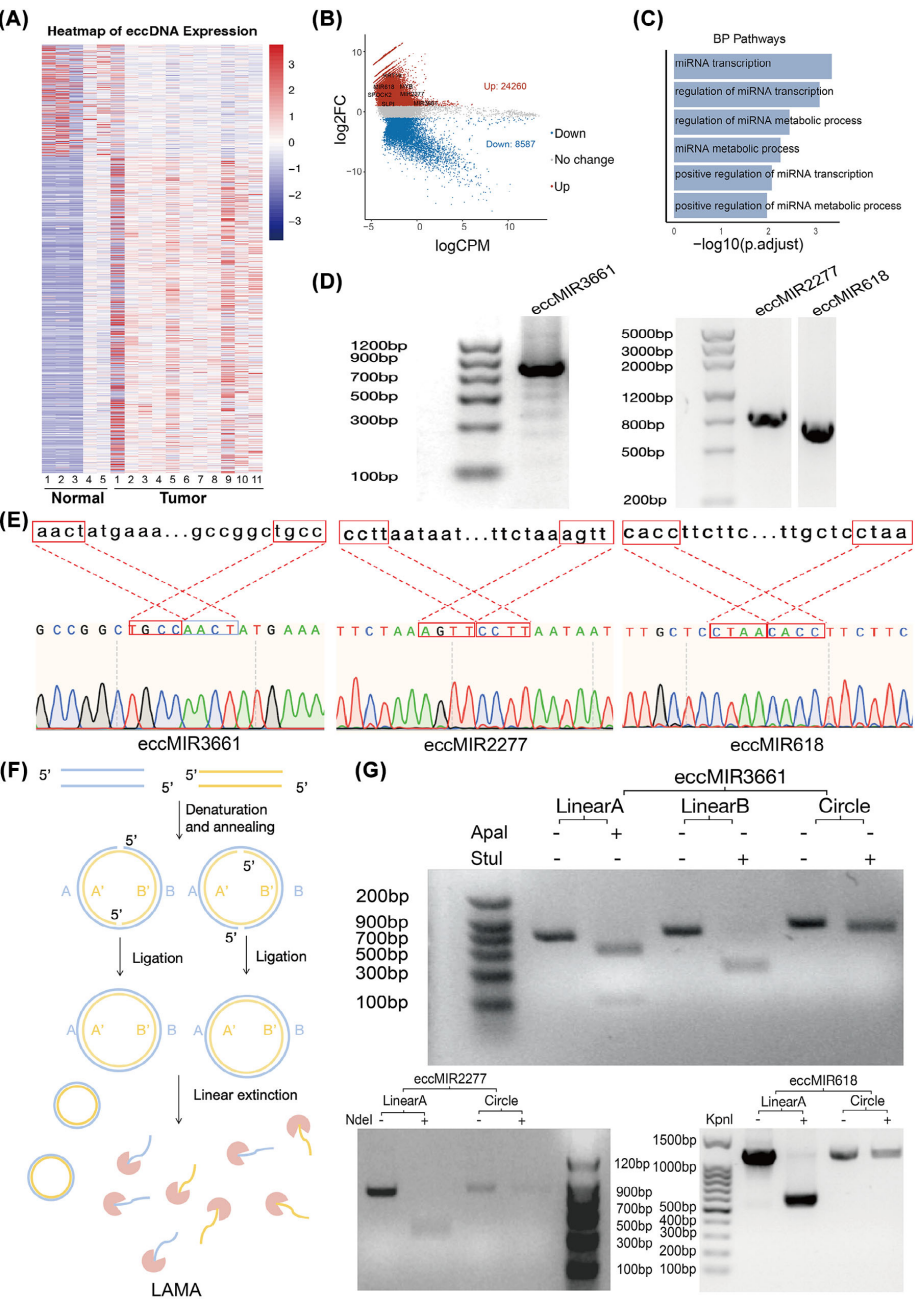
Validation and synthesis of upregulated eccMIRs in OC. (A) Heatmap showing differentially abundant eccDNA between OC and normal ovarian tissues identified by edgeR. (B) Volcano map showing differences in eccDNA abundance between OC and normal ovarian tissues. Each point represents an eccDNA, with log fold change (logFC) on the *y*‐axis and log counts per million (logCPM) on the *x*‐axis. Differential analysis was based on raw eccDNA read counts (sum of split, discordant and concordant reads). Significance thresholds were set as FDR‐adjusted *p*‐value ≤ .05 and logFC ≥ 1. eccDNAs meeting these criteria are coloured as ‘up’ (red) or ‘down’ (blue); others are labelled as ‘no’ (grey). Note that logCPM reflects edgeR's internal normalisation and is used here solely for visualisation, while eccDNA quantification across samples was performed using logTPM, as detailed in the Methods. (C) GO analysis of upregulated eccDNA‐associated genes in OC tissues. *x*‐Axis: significance of pathway analysis [−log10(adjusted *p*‐value)]. (D and E) Verification of eccMIR3661, eccMIR618 and eccMIR2277 by reverse PCR (D) and Sanger sequencing (E). (F) Flow diagram of eccDNA synthesis using the LAMA method: (1) synthesis of two semi‐complementary linear DNA fragments; (2) mixing equal amounts of linear A and B amplification products with Taq DNA ligase; (3) cleavage of LAMA DNA rings with an appropriate restriction enzyme to verify the circular structure. (G) Verification of the eccDNA synthesised by LAMA using restriction enzymes.

To further elucidate potential sequence determinants influencing eccDNA binding patterns in HGSOC tissue, we conducted an enrichment analysis of transcription factor binding motifs. Notably, enriched motifs associated with oncogenes (e.g., *MYB*, *TGIF2*, *DLX3*, *BATF*, *ZNF165* and *HOXB4*) were identified in HGSOC tissues (Figure S2B). After quantifying the eccDNA, we detected significant differential expression between HGSOC and normal samples (Figure 3A), with 32 847 eccDNAs (associated with 10 245 genes) showing altered levels (Table S1). Among these, 24 260 eccDNA were upregulated (related to 7691 genes, logFC > 2, adjusted *p*‐value < .05), while 8587 were downregulated (related to 3613 genes, logFC > .5, adjusted *p*‐value < .05) (Figure 3B). Previous research has demonstrated a positive correlation between eccDNA and the amplification of oncogenes, as well as cancer progression.^44^, ^45^ Consistent with this, we identified upregulated eccDNA harbouring oncogenes (e.g., *SPOCK2*, *SLC4A11, KLHL14*, *KRT19*, *RHPN2* and *SLPI*), which were associated with poor prognosis in HGSOC (Figure 3B). Gene expression profiling interactive analysis (GEPIA) further validated their elevated expression and survival significance (Figure S2C,D). To further explore the mechanisms promoting the progression of OC, we conducted GO analysis on the upregulated eccDNA in HGSOC. We found that the upregulated eccDNA were enriched in miRNA‐related processes, including the miRNA transcription (adjusted *p*‐value = .00046), miRNA transcription regulation (adjusted *p*‐value = .00083), miRNA metabolism regulation (adjusted *p*‐value = .00049), and negative regulation of RNA metabolism (adjusted *p*‐value = .00083) (Figure 3C). Therefore, these findings suggest that upregulated miRNAs encoded by eccDNA may contribute to HGSOC progression through negative regulation of their target genes.

### 2.4 HGSOC over‑represented eccMIRs produced functional miRNA molecules in host cells

Most eccDNA in cancer cells and normal tissues is too small to accommodate full‐length protein‐coding genes.^30^ However, precursor microRNAs (pre‐miRNAs or pre‐miRs), which typically range from 70 to 90 nucleotides in length, are compact enough to be harboured by eccDNA.^46^, ^47^, ^48^ eccDNA that carries pre‐miRs has been demonstrated to inhibit the expression of endogenous target genes by generating transcripts that can be processed into mature miRNA molecules, independent of classical promoter sequences.^30^, ^49^ Building upon the known regulatory potential of eccMIRs in cancer, we systematically identified significantly upregulated eccMIRs in HGSOC (Figure 3B, Wilcoxon test, *p*‐value < .05) and validated three key candidates—eccMIR3661 (chr5: 134 225 103–134 225 887), eccMIR618 (chr12: 80 934 853–80 936 003), and eccMIR2277 (chr5: 93 620 688–93 623 324)—through reverse PCR and Sanger sequencing (Figure 3D,E and Figure S3A). Database analysis (dbDEMC, https://www.biosino.org/dbDEMC/index) confirmed overexpression of their corresponding mature miRNAs in HGSOC (Figure S3B). To establish clinical relevance, we integrated TCGA‐OC data, revealing that these miRNA loci show recurrent amplification (CN ≥ 3) in a subset of OC patients (Figure S3C). By integrating TCGA miRNA‐seq data with our copy number analysis, we observed a copy number‐dependent expression pattern for all three eccMIRs. Notably, miR‐2277 exhibited significantly elevated expression in samples with copy number gains (CN ≥ 3, *p*‐value < .001), demonstrating a clear dosage effect (Figure S3C,D). These coordinated genomic amplifications and concomitant expression increases provide compelling evidence that eccMIR3661, eccMIR618 and eccMIR2277 may function as oncogenic drivers in ovarian carcinogenesis.

**FIGURE 4:**
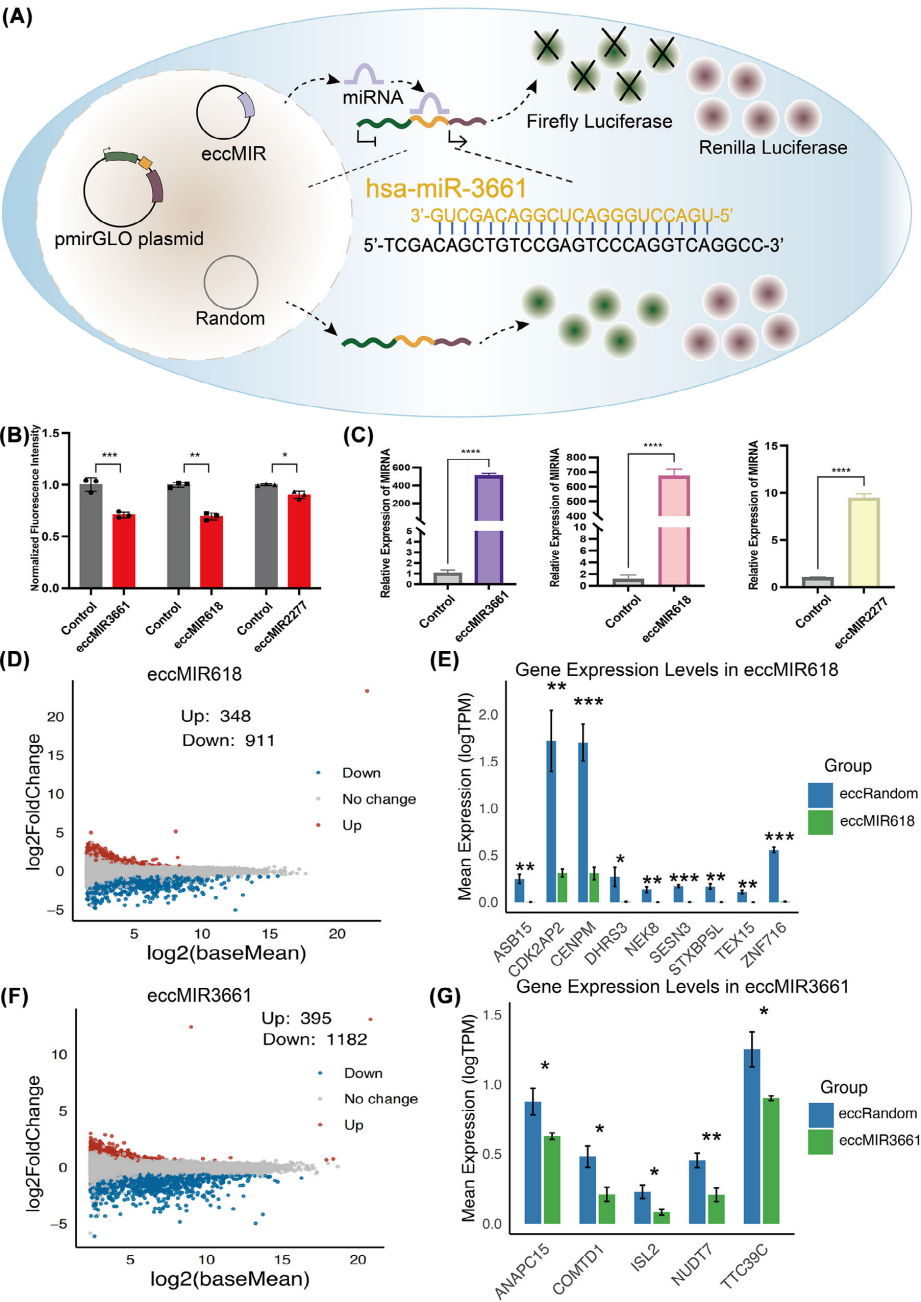
Validation of functionally expressed eccDNA carrying miRNA. (A) Schematic of the dual‐luciferase reporter gene assay for eccMIRs in SKOV3 cells. Synthetic eccMIRs were co‐transfected with the pmirGLO plasmid containing the miRNA‐target sequence in the 3′ UTR of the Firefly luciferase gene. eccMIRs produced miRNA molecules intracellularly, leading to repression of Firefly luciferase expression. Renilla luciferase was used as an internal control. (B) The normalised expression of Firefly luciferase in SKOV3 cells after transfection of eccMIRs. (C) Validation of miRNA expression by qPCR after eccMIRs transfection. (D) Volcano plot of differentially expressed genes from RNA‐seq analysis in SKOV3 cells transfected with eccMIR618. (E) Downregulated MIR618 targets in SKOV3 cells transfected with eccMIR618. (F) Volcano plot of differentially expressed genes from RNA‐seq analysis in SKOV3 cells transfected with eccMIR3661. (G) Downregulated MIR3661 targets in SKOV3 cells transfected with eccMIR3661. Data are presented as mean ± SE of three or four independent experiments. Significance was determined by Student's *t*‐test: ns, not significant; *p < .05, **p < .01, ***p < .001 and ****p < .0001.

To functionally characterise the identified eccMIRs, we first synthesised three candidate eccMIRs (eccMIR3661, eccMIR2277 and eccMIR618; 700–1 503 bp) using the ligase‐assisted minicircle accumulation method (LAMA), along with a 700 bp random eccDNA control (Figure 3F). The circular nature of these synthetic constructs was rigorously validated through exonuclease V resistance and restriction endonuclease digestion assays (Figure 3G), confirming their structural integrity for subsequent functional studies. We first confirmed that the artificial candidate eccMIRs can be successfully transcribed within cells using a dual luciferase reporter gene assay. This involved co‐transfecting SKOV3 cells with a pmirGLO plasmid (Promega) containing a target sequence complementary to the microRNA in the firefly 3′ UTR, alongside a synthetic eccMIR. The signal was measured 48 h post‐transfection (Figure 4A). Our results showed that the three identified eccMIRs significantly inhibited the corresponding reporter gene containing the miRNA target 3′ UTR sequences, with inhibition efficiencies ranging from 10% to 35% (Figure 4B). In addition, we transfected each kind of eccMIR and eccRandom into SKOV3 cells and assessed the relative expression levels of miRNA produced by the corresponding eccMIRs using quantitative PCR (qPCR). The results indicate that these eccMIRs can generate miRNA with high efficiency, exceeding that in the control group by more than 10‐fold (Figure 4C). In conclusion, both dual luciferase reporter gene assays and endogenous miRNA qPCR assays demonstrated that eccMIRs synthesised by LAMA were capable of generating functional miRNA molecules.

To further investigate the downstream pathway of eccMIRs in HGSOC, we transfected the relevant eccMIRs and validated their downstream targets in the SKOV3 cell line. The predicted target gene set for microRNAs (MIR3661, MIR618 and MIR2277) was obtained using miRDB (https://mirdb.org/).^50^ The downstream targets of eccMIR2277 were validated through qPCR analysis conducted at 48 h post‐transfection (Figure S3E). In addition, we conducted transcriptomic sequencing to identify the downstream targets of eccMIR3661 and eccMIR618. Based on the results of RNA sequencing, we further confirmed that the synthesised eccMIR was capable of expressing the corresponding miRNA (Tables S2 and S3). By integrating the downregulated genes identified from RNA sequencing data (*p*‐value < .05, logFC < −1), we successfully captured differentially expressed genes and downstream targets of eccMIR618 and eccMIR3661 (Figure 4D–G and Figure S3F). After the overexpression of eccMIR618 and eccMIR3661, the upregulated genes exhibit significant enrichment in pathways associated with tumourigenesis, such as the TGF‐β and NF‐κB signalling pathways (Figure S3G). The study illustrates the functionality of eccMIRs in regulating gene expression by suppressing target genes.

### 2.5 Candidate eccMIRs promote HGSOC cell growth and metastasis

**FIGURE 5:**
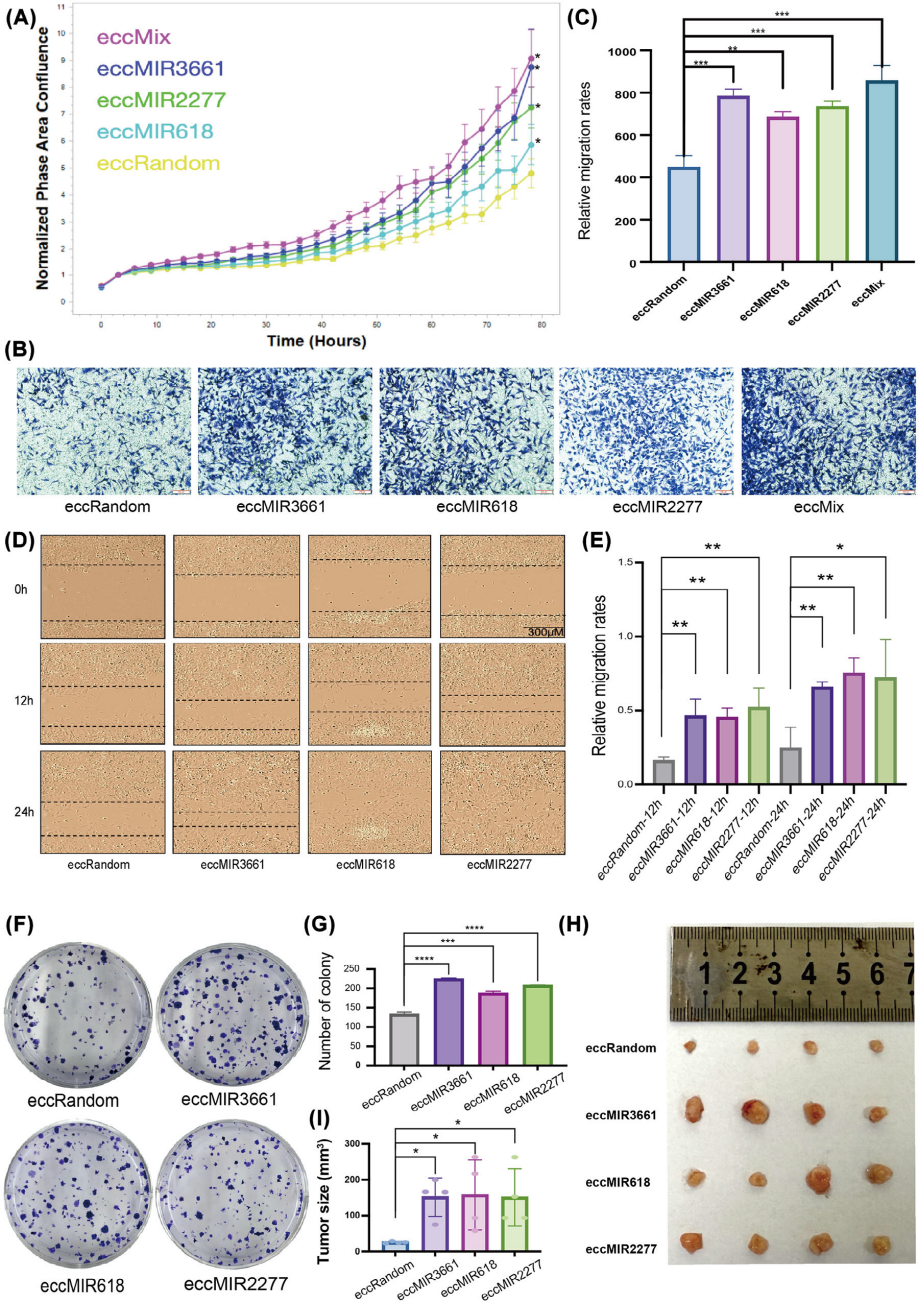
eccMIRs promote ovarian cancer malignancy by upregulating corresponding linear miRNAs in vitro and in vivo. (A) Cell viability assay was used to detect the proliferation ability of SKOV3 cells after transfection with eccMIR3661, eccMIR618, eccMIR2277 and eccMix. (B and C) The migration and invasion abilities of SKOV3 cells transfected with eccMIR3661, eccMIR618, eccMIR2277 and eccMix were detected by Transwell. Scale bar: 200 µm. (D and E) The migration and invasion abilities of SKOV3 cells transfected with eccMIR3661, eccMIR618 and eccMIR2277 were detected by the Scratch test. Scale bar: 300 µm. (F and G) The proliferation ability of SKOV3 cells transfected with eccMIR3661, eccMIR618 and eccMIR2277 was detected by a flat plate cloning experiment. (H and I) The images of the collected xenograft tumours (H) and tumour size (I). Data are presented as mean ± SE of three or four independent experiments. Significance was determined by Student's *t*‐test: ns, not significant; *p < .05, **p < .01, ***p < .001 and ****p < .0001.

While previous studies have established that eccMIRs can generate functional miRNAs,^32^ their direct roles in tumour growth and progression remain uncharacterised. To address this, we transfected three candidate eccMIRs (eccMIR3661, eccMIR618 and eccMIR2277) into HGSOC‐derived SKOV3 cells, with eccRandom‐transfected cells serving as controls, and systematically assessed their effects on proliferation, invasion, migration and clonogenic potential (Figure 5). We observed that the overexpression of eccMIR3661, eccMIR618 and eccMIR2277 significantly promoted the proliferation of SKOV3 cells (Figure 5A). Additionally, scratch assays and Transwell migration experiments further demonstrated that the migratory and invasive capabilities of SKOV3 cells were enhanced following transfection with eccMIR3661, eccMIR618 and eccMIR2277 (Figure 5B–D). Notably, OVCAR8 cells displayed comparable oncogenic phenotypes upon eccMIR transfection (Figure S4). In addition, we performed functional assays to evaluate the combinatorial effects of eccMIR3661, eccMIR618 and eccMIR2277 (eccMix). While the eccMix showed significantly stronger pro‐tumour effects (proliferation/migration/invasion, *p*‐value < .05 vs. control) than single eccMIR transfections, the enhancement over individual eccMIRs did not reach statistical significance, suggesting potential functional redundancy or pathway saturation (Figure 5E–G). In general, our findings suggest that eccMIR overexpression promotes the growth, survival and progression of HGSOC cells in vitro. Consistent with our in vitro findings, xenograft models revealed that eccMIR‐transfected SKOV3 cells (eccMIR3661/2277/618) formed significantly larger tumours than controls (Figure 5H,I), confirming that eccMIR‐driven oncogenic pathways remain functional in vivo. This strongly demonstrates the significant role of tumour‐specific formation of eccMIRs in tumourigenesis and development

## 3 DISCUSSION

The present study provides novel insights into the biogenesis mechanisms and functional implications of eccDNA in HGSOC, with a particular emphasis on its role in driving oncogenic processes through miRNA regulation. Our findings reveal that eccDNA generation in HGSOC is linked to microhomology‐mediated repair pathways and that eccDNA‐derived miRNAs contribute significantly to tumour progression by modulating critical signalling networks. These results expand our understanding of the molecular landscape of HGSOC and highlight eccDNA as a multifaceted player in cancer biology.^18^, ^23^, ^49^

### 3.1 Mechanistic insights into eccDNA biogenesis

The formation of eccDNA in HGSOC appears to be predominantly mediated by MMEJ, a DNA repair pathway characterised by its reliance on short homologous sequences (5–25 bp) for ligation of DSBs. This result is consistent with existing research,^40^, ^51^ and our innovation lies in analysing the regulatory network of the repair pathway at the overall level based on the eccDNA omics map of the OC cell line (rather than local gene loci). Our observation that HGSOC‐associated eccDNA harbours shorter microhomologous sequences at junction sites compared to normal tissues aligns with the heightened genomic instability and replication stress characteristic of advanced tumours.^52^, ^53^ The dependency on LIG3 and POLQ—key enzymes in the MMEJ pathway—for eccDNA generation underscores the mechanistic role of error‐prone repair processes in HGSOC. This is consistent with prior studies showing that MMEJ contributes to oncogene amplification and chromosomal rearrangements in cancer.^33^, ^54^, ^55^ Intriguingly, *KU80, DNA‐PKcs* and *RAD51* knockdown paradoxically increased eccDNA abundance, suggesting that suppression of NHEJ and HR—high‐fidelity repair mechanism—forces cells to rely on alternative repair pathways like MMEJ, thereby exacerbating eccDNA production. In HGSOC, frequent mutations of HR pathway genes such as *BRCA1/2* lead to defects in HR, forcing cells to rely on compensatory repair pathways. At this time, MMEJ is activated due to the release of HR inhibition, and the functional attenuation of NHEJ (such as low expression of *DNA‐PKcs*) further weakens the inhibitory effect on MMEJ. This imbalance in the repair network eventually drives a significant increase in the eccDNA load.^56^

Notably, the size‐dependent variation in homologous sequence length implies distinct biogenesis pathways for small (<10 kb) versus large (>100 kb) eccDNA. Smaller eccDNA, enriched in HGSOC, likely arise via MMEJ‐mediated repair of fragmented DNA, while larger structures may originate from NHEJ and HR repair pathway or chromothripsis.^38^, ^57^ The enrichment of eccDNA in promoter and coding regions further suggests that transcriptionally active loci are hotspots for DSBs, rendering them susceptible to circularisation. This spatial bias may facilitate the amplification of oncogenic elements, as observed in other cancers.^13^, ^21^

### 3.2 Functional roles of miRNA‐carrying eccDNA in HGSOC pathogenesis

Our observation of eccDNA enrichment in regulatory elements (Figure 1E) in HGSOC aligns with prior reports of ecDNA hubs amplifying oncogenic enhancers in glioblastoma.^21^ However, whereas glioblastoma ecDNA primarily carries whole‐gene amplifications (e.g., EGFR), our data reveal that HGSOC eccDNA frequently harbours fragmented regulatory sequences (e.g., promoter‐derived eccMIRs), suggesting tissue‐specific biogenesis mechanisms. Our study pioneers the exploration of eccDNA as a vehicle for functional non‐coding RNAs in HGSOC. The identification of eccMIRs harbouring pre‐miRNAs (eccMIR3661, eccMIR618 and eccMIR2277) and their capacity to generate mature miRNAs reveal a novel mechanism of oncogenic signalling. These eccMIRs act as mobile transcriptional units, bypassing canonical regulatory constraints to overexpress miRNAs that target tumour suppressor pathways. For instance, eccMIR618‐driven activation of TGF‐β signalling may promote epithelial–mesenchymal transition (EMT), a hallmark of OC metastasis.^58^ Similarly, eccMIR3661‐mediated activation of NF‐κB signalling could enhance cell survival and chemoresistance, aligning with the pro‐tumourigenic roles of this pathway in OC.^59^ Through in vivo and in vitro experiments, we demonstrated the functional consequences of eccMIR overexpression—enhanced proliferation, migration and invasion—highlighting their contribution to OC aggressiveness. However, eccMIRs may exert amplified oncogenic effects due to their ability to accumulate independently of chromosomal constraints, enabling rapid adaptation to therapeutic pressures. This ‘on‐demand’ amplification mechanism could explain the transient nature of drug resistance observed in OC patients, where eccDNA dynamics may facilitate clonal evolution.^26^

### 3.3 Clinical implications and future directions

The discovery of eccMIRs as functional regulators in HGSOC opens new avenues for therapeutic intervention. Targeting the MMEJ pathway (e.g., via *LIG3* inhibitors and *POLQ* inhibitors) could disrupt eccDNA biogenesis, potentially sensitising tumours to chemotherapy. Additionally, eccMIR‐derived miRNAs or their downstream targets (e.g., TGF‐β/NF‐κB components) may serve as biomarkers for early detection or prognostic stratification. The preferential localisation of eccDNA in regulatory regions further suggests that eccDNA profiles could reflect transcriptional vulnerabilities exploitable for precision therapies.

However, several questions remain unresolved. First, although we ensure the quality of specimens through strict inclusion criteria, the scarcity of high‐quality clinical specimens objectively causes an insufficient sample size. Second, the origin of eccMIRs—whether they arise from specific genomic loci under replication stress or random fragmentation—requires further investigation. Third, the crosstalk between eccDNA and the tumour microenvironment, particularly in peritoneal metastasis, remains unexplored. Finally, longitudinal studies tracking eccDNA dynamics during chemotherapy are needed to assess their role in acquired resistance.

In conclusion, our work establishes eccDNA as a facilitator of HGSOC progression, acting through non‐coding RNA‐mediated signalling. By elucidating the MMEJ‐dependent biogenesis of eccDNA and its functional interplay with miRNA networks, this study provides a foundation for developing eccDNA‐centric therapeutic strategies in HGSOC and other malignancies characterised by rampant genomic instability.

## 4 MATERIALS AND METHODS

### 4.1 Cell culture

The human HGSOC cell lines SKOV3 and OVCRA8 were obtained from the Cancer Center of Peking University Third Hospital. SKOV3 cells were maintained in Ham's F‐12 medium (HyClone, SH30026.01). OVCRA8 cells were maintained in RPMI‐1640 (HyClone, SH30809.01). All cell lines were supplemented with 10% fetal bovine serum (FBS) (Biological Industries, 04001) and 1% penicillin/streptomycin (Gibco, 15140122), under standard culture conditions in a humidified atmosphere at 37°C with 5% CO_2_. Routine subculturing was performed using TATRYPSIN .25% EDTA (Gibco, 25200072) upon reaching 80%–90% confluency. All cell lines were regularly tested for mycoplasma contamination and authenticated using short tandem repeat (STR) profiling.

### 4.2 HGSOC patients’ recruitment and samples’ collection

With the approval of the Human Ethics Committee of Peking University Third Hospital (ethical approval No.: M2024522), ovarian cancer tissues were collected from 11 patients with HGSOC, while healthy ovarian tissues were obtained from five patients with non‐ovarian gynaecological tumours. All patients underwent total hysterectomy with bilateral salpingo‐oophorectomy, and the ovarian tissues from the latter group were histopathologically confirmed as normal. Prior to sampling, the patient signed an informed consent form certified by the institute. None of the patients in this study had received chemotherapy or radiotherapy before surgery.

### 4.3 eccDNA purification and Circle‐seq

The eccDNA was purified and amplified according to previous research methods.^34^ Briefly, samples (∼30 mg) were cut into small pieces, and genomic DNA was extracted using the MagAtract HMW DNA Kit (Qiagen, 67563). Two micrograms of genomic DNA was added to each sample, and linear DNA was removed with plasmid safe ATP‐dependent DNase, according to the manufacturer's instructions. The reaction is carried out continuously at 37°C for 2 days with the addition of an additional 1 µL of ATP and DNase every 24 h, according to the manufacturer's agreement. Standard PCR methods were employed to validate the elimination of linear DNA (*COX5B*) in each sample (Figure S1A and Table S4). Subsequently, the purified eccDNA was continuously amplified at 30°C for 48 h using Phi29 polymerase (New England Biolabs, M0269S). DNA purification was performed using phenol‐chloroform‐isoamyl alcohol (PCIA). Libraries for next‐generation sequencing were prepared utilising the NEBNext Ultra DNA library kit for Illumina (New England Biolabs, E7103) following the manufacturer's protocol. All libraries were sequenced on an Illumina Novaseq 6000 platform using paired‐end 150 sequencing.

### 4.4 Circle‐seq data processing and eccDNA quantification

Raw sequencing reads generated from Circle‐seq libraries were first subjected to quality control using FastQC, followed by adapter and quality trimming using Trim Galore with the parameters ‐q 20 –phred33 –stringency 3 –length 20 ‐e 0.1. Cleaned paired‐end reads were then aligned to the Ensembl GRCh38 Release 107 reference genome using BWA‐MEM with default parameters. To reduce false‐positives, we performed alignment against a masked reference genome, in which interspersed repeats and low‐complexity regions were masked using RepeatMasker (replaced with ‘N’), and further filtered regions from the ENCODE blacklist (https://github.com/Boyle‐Lab/Blacklist/) were also excluded.

To accurately identify eccDNA, we used Circle‐Map v1.1.4 (https://github.com/iprada/Circle‐Map).^60^ The mapped BAM files were sorted both by read names and by genomic coordinates to facilitate circular structure reconstruction.^61^ Circle‐Map was then applied to detect candidate eccDNA based on split and discordant reads. We applied the following stringent filtering criteria to retain high‐confidence eccDNA: (i) ≥2 supporting split reads; (ii) ≥2 supporting discordant read pairs; (iii) circular DNA candidates with length less than 50 Mb.^60^

For quantification of each eccDNA, we implemented a custom read‐counting strategy: (i) read types—split, discordant and concordant—were classified using samblaster^62^; (ii) the number of each read type mapped to each identified eccDNA was counted using BEDTools v2.30.0^63^; (iii) the total read count for each eccDNA was calculated as the sum of split, discordant and concordant reads; (iv) to calculate TPM (transcripts per million) values, the read count of each eccDNA was first divided by its length in kilobases to obtain reads per kilobase (RPK). Then, the sum of all RPK values in each sample was computed. Finally, the TPM for each eccDNA was calculated by dividing its RPK by the total RPK sum and multiplying the result by one million:

$$\mathrm{TPM}=\frac{\left(\mathrm{Read}\;\mathrm{count}/\mathrm{eccDNA}\;\mathrm{length}\;\mathrm{in}\;\mathrm{kb}\right)}{\left(\mathrm{Sum}\;\mathrm{of}\;\mathrm{all}\;\mathrm{RPKs}\;\mathrm{in}\;\mathrm{the}\;\mathrm{sample}\right)}\times \;1\;000\;000.$$

This normalisation method accounts for both eccDNA length and total sequencing depth, enabling meaningful comparisons of eccDNA abundance across different samples and conditions.

### 4.5 Genomic distribution of eccDNA

To delineate the genomic localisation of eccDNA, it was designated according to the priority order (promoter > 5′ UTR > 3′ UTR > exon > intron > downstream > intergenic) facilitated by ChIPseeker (version 1.34.1) in cases where a single eccDNA encompassed multiple genomic features.^64^

### 4.6 GO enrichment analysis

GO enrichment analysis was conducted utilising the ‘enrichGO’ functions of the ClusterProfiler package (version 4.6.0).^64^ The *p*‐values were adjusted using the Benjamini–Hochberg correction to account for multiple comparisons, focusing on statistically significant enrichment.

### 4.7 Characterisation of the eccDNA junction sites

BEDTools was employed to extract DNA sequences, comprising 20 base pairs upstream and downstream of every eccDNA junction.

### 4.8 Motif‐enrichment analysis

Motif enrichment analyses were conducted using HOMER (v4.11) findMotifsGenome.pl algorithm with the parameters ‘‐size 200 and ‐mask’, leading to known enrichment results and de novo enrichment results; the former were selected and utilised.^65^

### 4.9 Inverse PCR

The junction region of the eccDNA of interest was validated using inverse PCR, followed by Sanger sequencing. Each 25 µL PCR reaction contained 1 µL of RCA product, 2 µL of forward primer (5 µM), 2 µL of reverse primer (5 µM), 12.5 µL 2× Rapid Taq Master mixture (Vazyme, P222‐01), and ddH_2_O to adjust the final volume. The PCR procedure was as follows: 95°C for 5 min; (95°C for 15 s, 55°C for 15 s, 72°C for 5 s), 35 cycles; 72°C for 5 min, and hold at 4°C. The primers used for validating eccMIRs are listed in Table S4.

### 4.10 Synthesise eccDNA using the LAMA method

The synthetic eccDNA was constructed using the LAMA method.^30^ As a control, a random 700 bp DNA sequence with 50% guanine‐cytosine content was generated using the ‘Random DNA Sequence Generator’ web tool (http://www.faculty.ucr.edu/~mmaduro/random.htm) (eccRandom).^66^ Specifically, eccRandom is a shuffled sequence with no homologous regions in the genome. The half‐complementary linear DNA fragments (linear A and B) corresponding to each candidate eccDNA, along with the PCR primers, are listed in the Table S4. 500 ng of linear amplification products A and B were mixed with 1 µL of Taq DNA ligase (New England Biolabs, M0208V) and 2.5 µL of 10× corresponding reaction buffer, and the 25 µL system was supplemented with DNase/RNase‐free water. The thermal cycle system used was as follows: 5 min at 95°C, (95°C 20 s, 4°C 1 min, 55°C 20 min) with 10 cycles. The residual linear DNA was removed by Exonuclease V (New England Biolabs, M0345S), and the LAMA reaction products were purified by VAHTS DNA Clean Beads (Vazyme, N411‐02).^67^ Then the synthetic eccDNA was digested by certain restriction enzymes using the linear fragments as controls to verify its circular structure. eccDNA with only one linear band after restriction enzyme digestion was selected for subsequent experiments.

### 4.11 Cell transfection with synthetic eccDNA

Transfection was performed using Lipomaster 2000 Transfection Reagent (Vazyme, TL201‐01) in a 24‐well plate according to the manufacturer's instructions. Briefly, 60 000 cells were seeded into each well of a 24‐well plate 24 h prior to transfection. For each transfection, 500 ng of synthesised eccDNA was mixed with 2.5 µL of liposome 2000 and supplemented with Opti‐MEM (Gibco, 31985062) to prepare the transfection complex. Each experimental group was transfected in at least three independent replicates. Cells were harvested 48 h post‐transfection for downstream analysis. In this study, eccRandom was used as the control group for all transfection experiments.

### 4.12 qPCR assay of miRNA and mRNA

Following 48‐h transfection with synthesised eccDNA, cells were harvested for total RNA extraction using the FastPure Cell/Tissue Total RNA Isolation Kit V2 (Vazyme, RC112‐01). For miRNA analysis, first‐strand cDNA synthesis was performed with the miRNA 1st Strand cDNA Synthesis Kit (by stem‐loop) (Vazyme, MR101‐01), with U6 snRNA serving as the endogenous control co‐reverse transcribed in the same reaction. MiRNA quantification was carried out using miRNA Universal SYBR qPCR Master Mix (Vazyme, MQ101‐01) according to manufacturer specifications. Parallel mRNA analysis involved cDNA synthesis using HiScript III 1st Strand cDNA Synthesis Kit (+gDNA wiper) (Vazyme, R312‐02), followed by qPCR amplification with Taq Pro Universal SYBR qPCR Master Mix (Vazyme, MQ101‐02). All reactions were performed in triplicate, with relative expression levels of both miRNA and mRNA calculated using the 2^−ΔΔCt^ method. Primers used here are detailed in Table S4.

### 4.13 Luciferase assays

DNA oligonucleotides encoding mature miRNAs derived from microDNA (miR3661, miR618 and miR2277) were cloned into the 3ʹ UTR region of the firefly luciferase gene within the pmirGLO dual‐luciferase reporter vector (Promega). The miRNA complementary sequences were inserted in the sense (+) orientation (Table S5). To assess miRNA activity, transfected cells were lysed and analysed using the Dual‐Luciferase Reporter Assay System (Promega, E1910), with firefly luciferase signal normalised to Renilla luciferase as an internal control.

### 4.14 RNA sequencing

After transfection with eccMIR3661 and eccMIR618, cells were harvested 48 h post‐transfection, and cell lysates were collected. Libraries were then constructed and sequenced following the Smart‐seq2 protocol. Briefly, low‐input cells were lysed to release RNA. Subsequently, reverse transcription PCR (RT‐PCR) was performed by adding MMLV reverse transcriptase, free dNTPs, oligo(dT) VN primer, betaine, MgCl_2_ and template‐switching oligonucleotide (TSO) to the lysate to generate double‐stranded cDNA. The cDNA was then subjected to library preparation, which included mechanical fragmentation, size selection of small fragments, adapter ligation, PCR amplification and purification using magnetic beads. Finally, the quality‐verified libraries were sequenced on the Illumina platform (sequencing services provided by Annoroad).

### 4.15 Processing of gene expression datasets

For each dataset incorporated, we first underwent quality control and read trimming.

Raw sequencing reads were first processed using fastp (version 0.23.2) to remove adapter sequences and low‐quality bases. The parameters ‐q 20 and ‐u 30 were applied to ensure high‐quality reads by trimming bases with a quality score below 20 and discarding reads with more than 30% of bases below the quality threshold. The trimmed reads were then aligned to the ENSEMBL hg38 reference genome using HISAT2 (version 2.2.1) with default parameters. The aligned reads were quantified using featureCounts (version 2.0.1). Reads were assigned to genes based on the Ensembl gene annotation file, with the parameters ‐p (to count paired‐end reads), ‐a (to specify the GTF annotation file) and ‐g (to define gene IDs).

To prepare for further analysis, raw read counts were normalised to transcripts per million (TPM) to account for differences in sequencing depth and gene length. To avoid issues with zero values during downstream analysis, a pseudocount of 1 was added to each TPM value. The normalised counts were then subjected to a log10 transformation to stabilise variance and ensure compatibility with statistical analyses.

### 4.16 The relationship between eccMIR copy number and expression

The gene‐level copy number data were derived from TCGA‐OC datasets using the ASCAT3 pipeline for processing. The miRNA expression data were obtained from public TCGA miRNA‐seq repositories. Based on their CNV levels, miRNAs were categorised into high‐ and low‐copy number groups. Subsequently, an integrated analysis of these data was performed to investigate the association between copy number alterations and expression trends.

### 4.17 RNAi knockdown

SKOV3 cells were seeded in six‐well plates at 60% confluence (2 mL medium per well). After 24‐h incubation, siRNA transfection complexes were prepared as follows: 10 µL of 20 µM siRNA targeting *LIG3, POLQ, KU80, DNA‐PKcs* and *RAD51* (Tsingke) was diluted in 100 µL Opti MEM (Gibco, 31985062), respectively, while 7.5 µL Lipomaster 2000 Transfection Reagent (Vazyme, TL201‐01) was separately mixed with 100 µL Opti‐MEM. The diluted siRNA and Lipofectamine solutions were mixed, incubated at room temperature for 15 min, and then added dropwise to the cell culture. Non‐targeting siRNA (si*Non*, Tsingke) was used as the negative control. After 5 h of transfection, the medium was replaced with fresh complete medium. Cells were harvested 48 h post‐transfection, and the knockdown efficiency was assessed by qPCR and Western blot. The siRNA sequences (si*LIG3*, si*POLQ*, si*KU80*, si*DNA‐PKcs*, si*RAD51* and si*Non*) and qPCR primers are provided in Table S4.

### 4.18 Western blot

Following siRNA‐mediated knockdown, SKOV3 cells were washed three times with PBS and harvested using cell scrapers. Total protein extraction was performed using ice‐cold RIPA lysis buffer (Beyotime, P0013B) through a 30‐min incubation on ice with intermittent vortexing at 10‐min intervals. Following centrifugation at 12 000 × *g* for 10 min at 4°C, the supernatant was collected and mixed with SDS‐PAGE loading buffer (Beyotime, P0015). The mixture was subsequently heat‐denatured at 95°C for 5 min. Protein samples were then separated via SDS‐PAGE and electrophoretically transferred onto PVDF membranes (Bio‐Rad, 1704271). Membranes were initially blocked with 5% non‐fat milk in TBST for 1 h at room temperature. Subsequently, they were incubated overnight at 4°C with the following primary antibodies: anti‐α‐tubulin (1:1000; Abcam, ab7291), anti‐PARP1 (1:1000; Abcam, ab191217), anti‐LIG3 (1:1000; Proteintech, 26583‐1‐AP), anti‐POLQ (1:1000; Proteintech, 28590‐1‐AP), anti‐DNA‐PKcs (1:1000; Abcam, ab3256), anti‐Ku80 (1:1000; Cell Signaling Technology, a12338), anti‐RAD51 (1:1000; Abcam, ab176458). Following three 10‐min TBST washes, membranes were incubated for 1 h at room temperature with species‐matched HRP‐conjugated secondary antibodies: either anti‐mouse IgG (1:5000; TransGen, HS201‐01) or anti‐rabbit IgG (1:5000; TransGen, HS101‐01). Protein signal detection was achieved using ECL Prime Western Blotting Substrate (Thermo Scientific, 34580), with chemiluminescent imaging performed on a ChemiDoc MP System (Bio‐Rad).

### 4.19 Cell proliferation experiment

SKOV3 cells were plated in 96‐well plates at a density of 5 × 10^3^ cells/well in 200 µL of complete growth medium consisting of Ham's F‐12 medium (HyClone, SH30026.01) supplemented with 10% FBS (Biological Industries, 04001). Plates were immediately transferred to a live‐cell imaging system (IncuCyte S3, Sartorius) housed in a humidified incubator at 37°C and 5% CO_2_. Phase‐contrast images were automatically acquired for 5 consecutive days, and cell proliferation kinetics were analysed using IncuCyte software (v2022A).

### 4.20 Scratch test

SKOV3 cells were seeded in 96‐well plates (Corning, 3599) at a density of 1 × 10^4^ cells/well and cultured to 90%–95% confluence in complete Ham's F‐12 medium (HyClone, SH30026.01) supplemented with 10% FBS (Biological Industries, 04001). Uniform linear scratches were generated using WoundMake (4493) under standardised pressure. Following the scratching procedure, three successive gentle PBS washes were performed to remove detached cells, with subsequent medium replacement using serum‐free Ham's F‐12 medium. Plates were immediately transferred to a live‐cell imaging system (IncuCyte S3, Sartorius) maintained at 37°C and 5% CO_2_, with phase‐contrast images captured for 24 h. Scratch closure dynamics were quantified by measuring normalised wound width reduction using ImageJ (v1.54f) with the MRI Wound Healing Tool plugin.

### 4.21 Transwell invasion assay

Cell migration capacity was evaluated using Corning Transwell chambers (8 µm pore size, 3422). Serum‐starved cells were seeded in the upper chamber at a density of 2 × 10^4^ cells/well in 120 µL serum‐free Ham's F‐12 medium (HyClone, SH30026.01). The lower chamber contained 600 µL Ham's F‐12 medium supplemented with 10% FBS (Biological Industries, 04001) as a chemoattractant. After 24‐h incubation at 37°C and 5% CO_2_, migrated cells on the membrane underside were fixed with 4% paraformaldehyde (PFA) for 30 min, stained with .1% crystal violet (Solarbio, G1064) for 20 min, and gently rinsed with PBS to remove non‐migratory cells. Three random bright‐field images per well were captured using an inverted microscope (Nikon). Migrated cells were quantified using threshold‐based area analysis in ImageJ (v1.534f).

### 4.22 Clonal formation

Cells were seeded at a density of 1 × 10^3^ cells/well in a six‐well plate (Corning, 3516). Subsequently, 2 mL of Ham's F12 medium (HyClone, SH30026.01) supplemented with 10% FBS (Biological Industries, 04001) was added to each well. The plates were then incubated in a cell culture incubator at 37°C and 5% CO_2_ for 14 days. After incubation, cells were fixed using a solution of 4% PFA at 4°C for approximately 20 min to preserve cellular architecture. Following fixation, staining was performed using a solution of crystal violet at a concentration of .1%, applied at room temperature for approximately 15–20 min. Cell coverage was quantified utilising ImageJ (v1.534f).

### 4.23 Xenograft mouse models

All animal experiments were conducted in compliance with Peking University's Institutional Animal Welfare and Ethics Committee guidelines (approval No. LA2021579). Four 5‐week‐old female BALB/c nude mice per group were randomly assigned to either experimental (eccMIR‐transfected SKOV3 cells) or control (eccRandom‐transfected) groups. Following a 48‐h transfection with synthetic eccMIR. Approximately 2 × 10^6^ viable cells suspended in a 1:1 PBS/Matrigel mixture (Corning 356234) were subcutaneously injected into the back of each mouse's forelimb. Tumour growth was monitored weekly by measuring dimensions with callipers, with volumes calculated as .52 × length × width^2^. Mice were humanely euthanased at 6 weeks post‐inoculation for tumour resection and final analysis.

### 4.24 Statistical analysis

Statistical evaluations were performed using the R programming. In the creation of boxplots, the median was depicted by the central line, while *p*‐values were computed using either the Wilcoxon–Mann–Whitney test through the ‘wilcox.test’ function or a Student's *t*‐test via the ‘t.test’ function. Significant differences were indicated by asterisks (*, **, ***), corresponding to *p*‐values less than .05, .01 and .001, respectively. The ggplot2 package (version 3.4.3) was used for generating most plots.

## AUTHOR CONTRIBUTIONS

X. Z., F. M. and G. H. conceived and designed the study. N. W. performed the experiments, drafted the manuscript and prepared the figures. L. W. performed the bioinformatic analysis and drafted figures. Q. L., T. H. and C. H. collected the samples. Y. J. assisted in conducting the experiments. X.Z, F. M. and K. L. revised the manuscript with input from all authors.

## CONFLICT OF INTEREST STATEMENT

The authors declare that they have no conflicts of interest.

## ETHICS STATEMENT

All procedures performed in studies involving human participants were in accordance with the ethical standards of the Human Ethics Committee of Peking University Third Hospital (ethical approval No.: M2024522), and with the 1964 Helsinki declaration and its later amendments or comparable ethical standards.

## Supporting information



Supporting Information

Supporting Information

Supporting Information

Supporting Information

Supporting Information

Supporting Information

Supporting Information

Supporting Information

Supporting Information

Supporting Information

## Data Availability

All data used in the paper are included in the paper and/or the Supporting Information. The data generated in this study are publicly available in the Genome Sequence Archive (GSA) under accession number HRA010542. Other raw data are available upon reasonable request from the corresponding author.
