# Supplementary figures and images for: Extrachromosomal circular DNA expressing miRNA promotes ovarian cancer progression
Source: Clin Transl Med. 2025 Sep 23;15(9):e70445. doi: 10.1002/ctm2.70445 (PMC12455017; doi:10.1002/ctm2.70445)

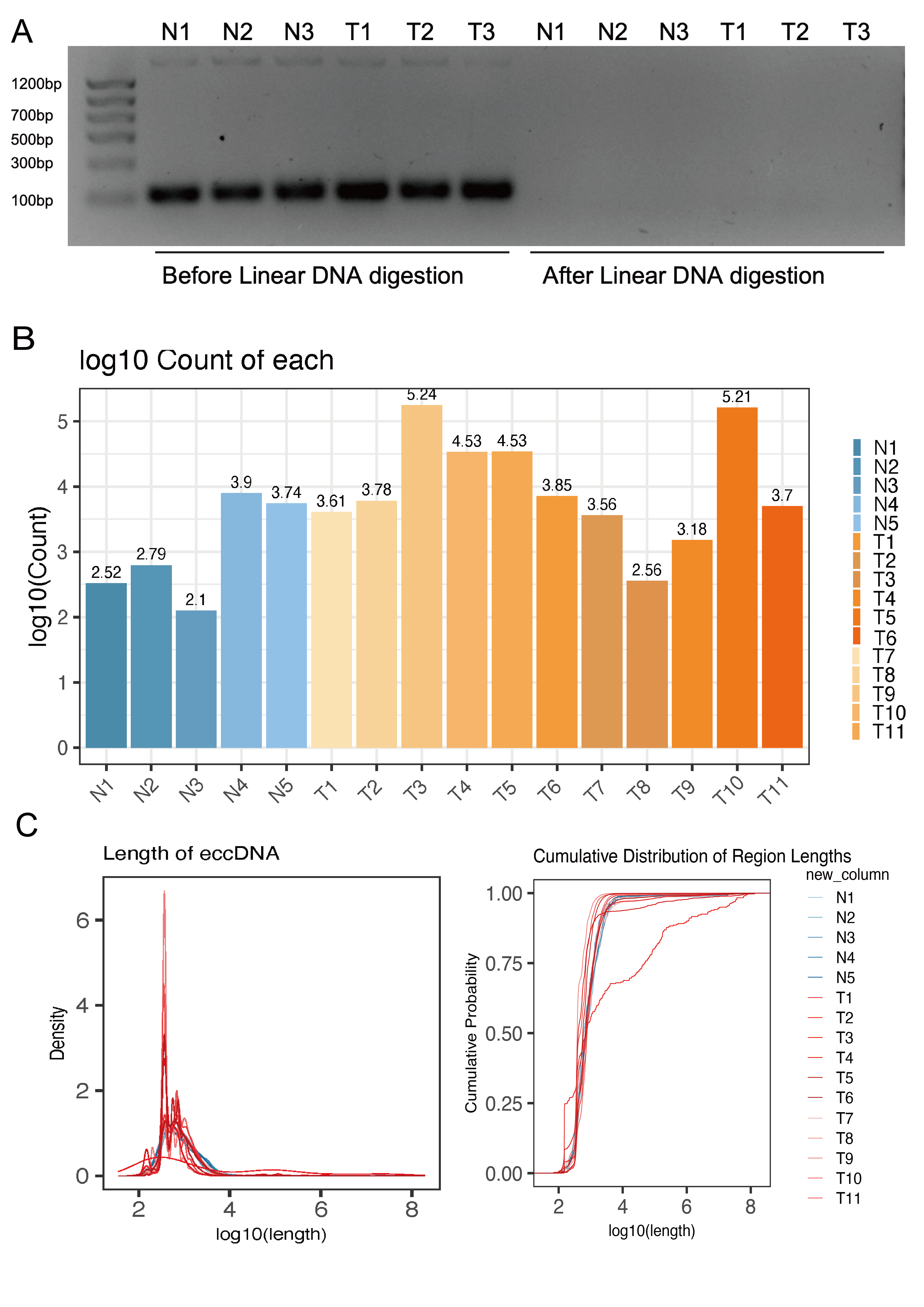

Supplement: Supplementary file 1 — Supporting Information [file CTM2-15-e70445-s006.png]

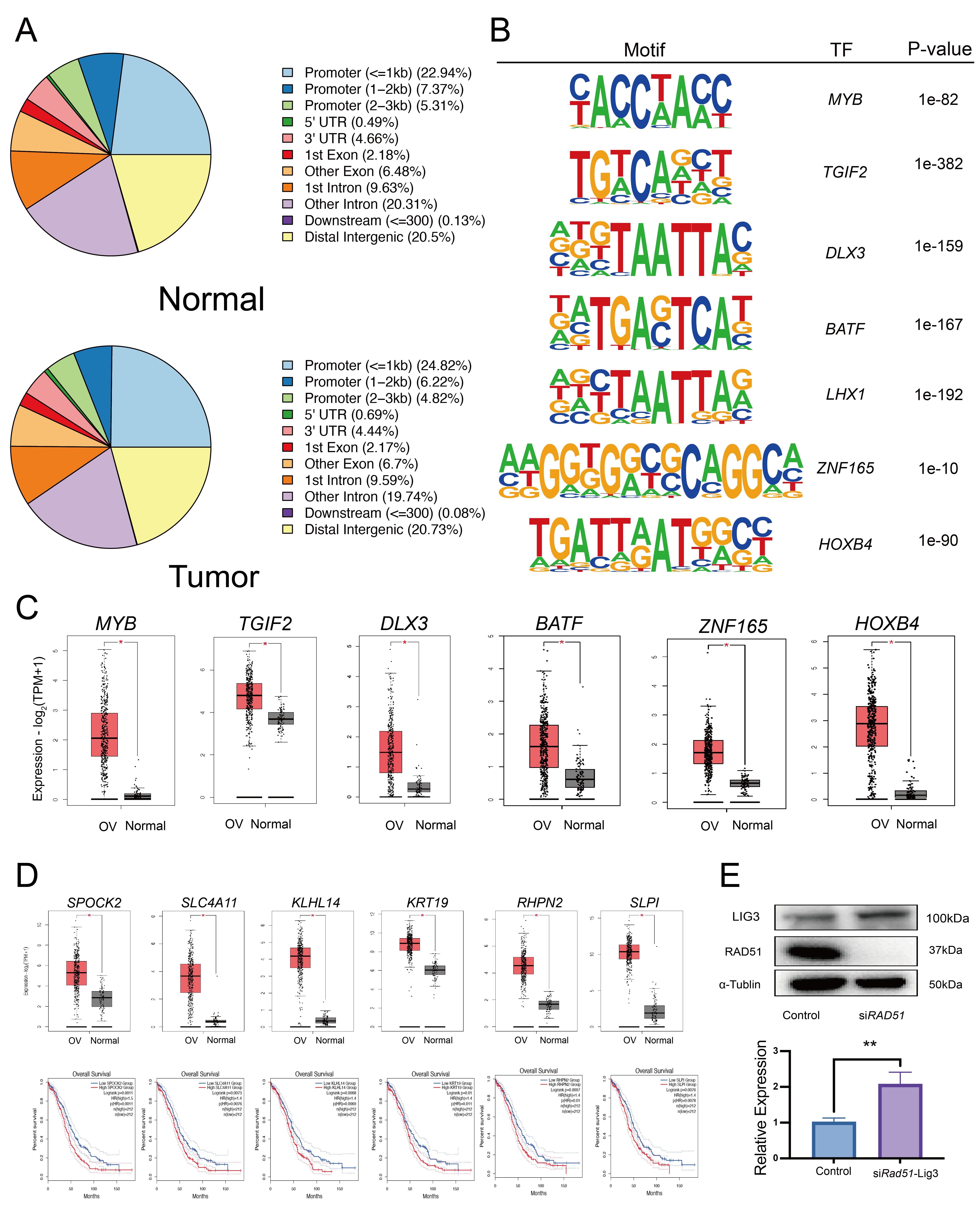

Supplement: Supplementary file 2 — Supporting Information [file CTM2-15-e70445-s008.png]

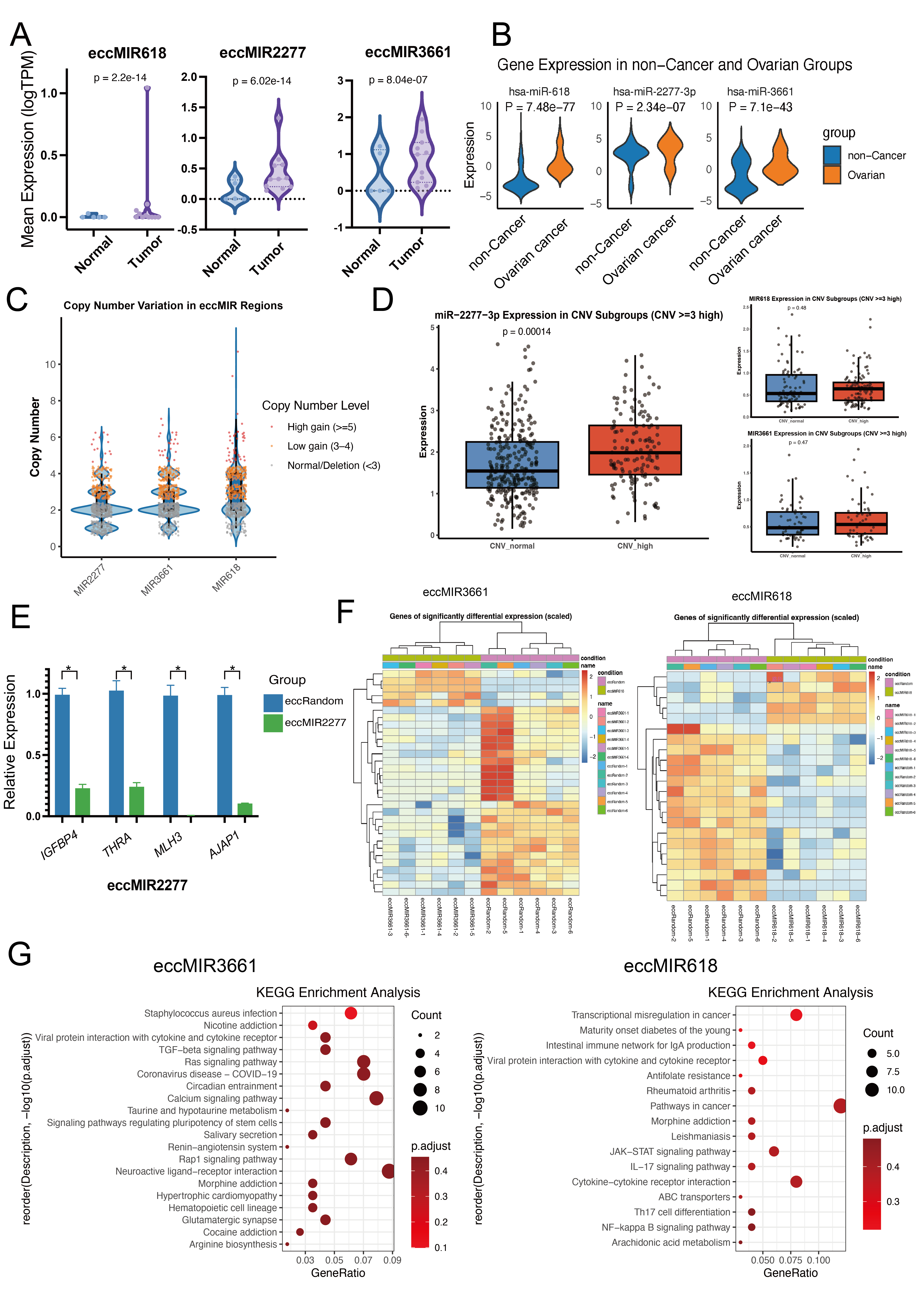

Supplement: Supplementary file 3 — Supporting Information [file CTM2-15-e70445-s001.png]

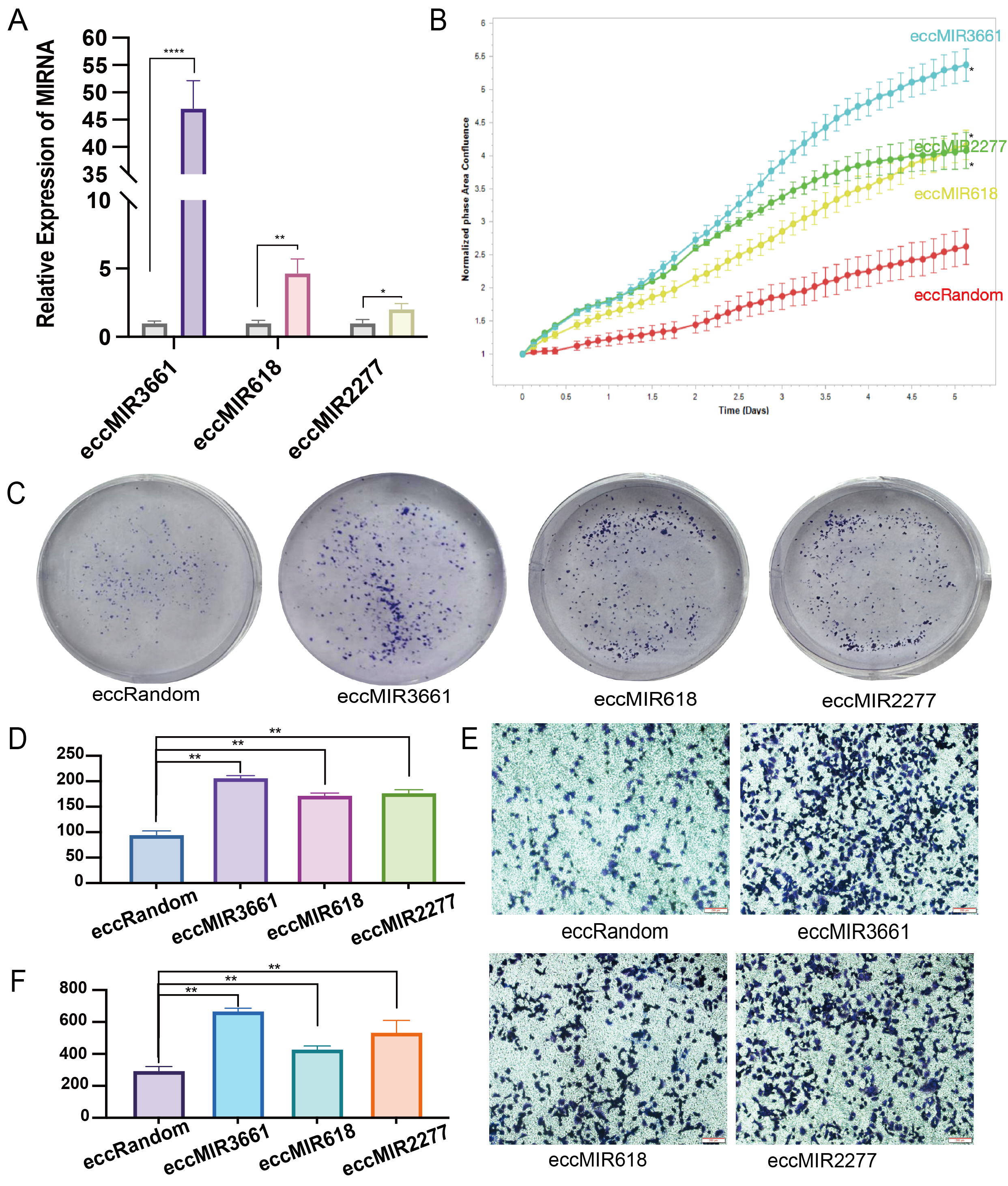

Supplement: Supplementary file 4 — Supporting Information [file CTM2-15-e70445-s002.png]
