# Supplementary material for: Extrachromosomal circular DNA expressing miRNA promotes ovarian cancer progression
Source: Clin Transl Med. 2025 Sep 23;15(9):e70445. doi: 10.1002/ctm2.70445 (PMC12455017; doi:10.1002/ctm2.70445)
Supplement: Supplementary file 5 — Supporting Information [file CTM2-15-e70445-s003.docx]

**Figure S1**


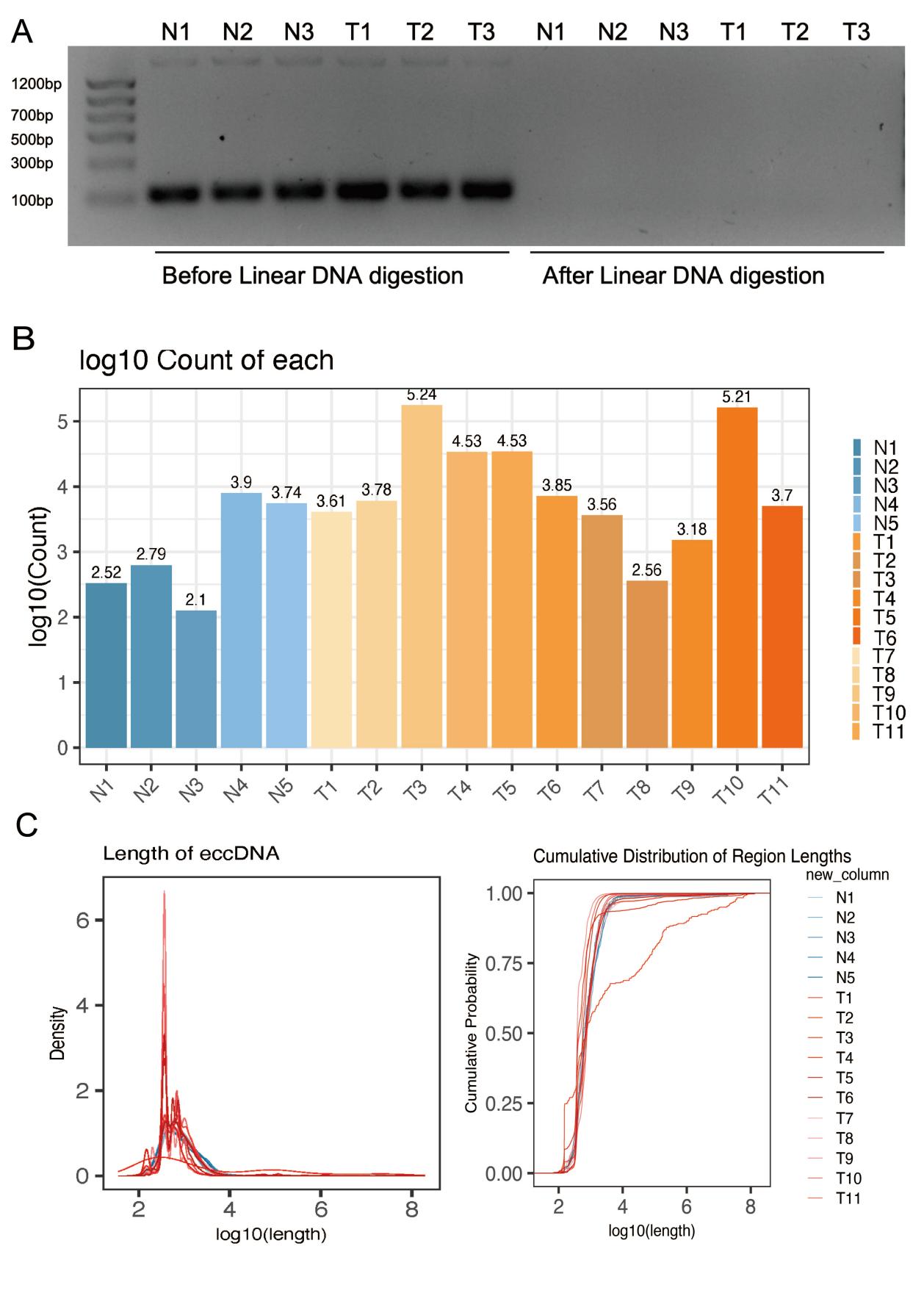


**Figure S1 eccDNA profile of ovarian cancer**

**(A)** validation of linear DNA elimination using the linear *COX5B* gene. (**B)** Number of eccDNA in individual OC and normal ovarian tissues. (**C)** Length distribution of eccDNA in individual OC and normal ovarian tissues.

**Figure S2**


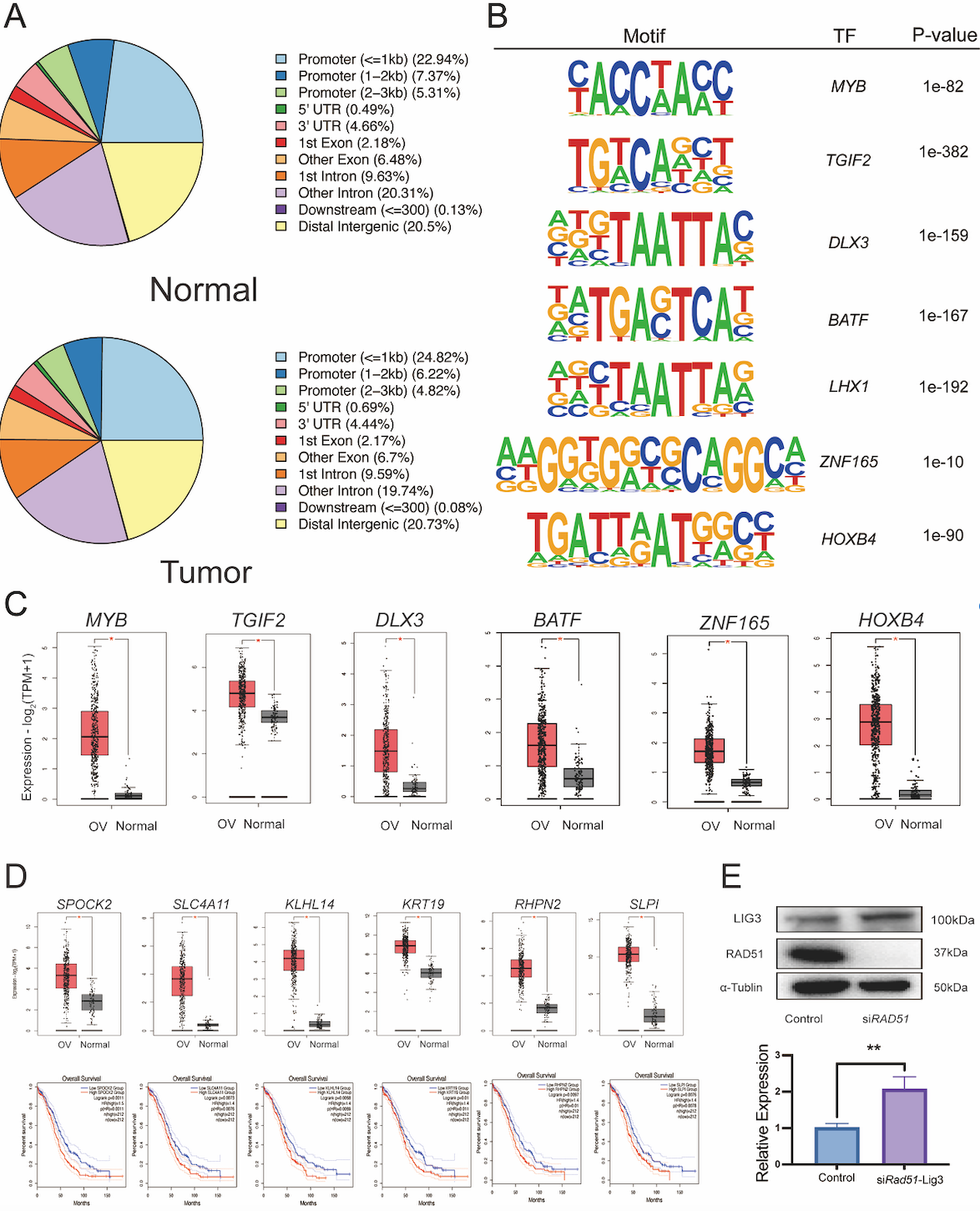


**Figure S2 eccDNA coding gene in ovarian cancer**

**(A)** Genomic regions enriched in eccDNA in OC and normal ovarian tissues. (**B)** Specified transcription factor motifs identified within 20 bp upstream and downstream sequences in OC tissues. (**C)** Expression profile of enriched transcription factor associated genes at eccDNA junction sites in OC patients and healthy people. Data sourced from the GEPIA website. (**D)** Expression profile and survival analysis of up-regulated eccDNA-containing genes in OC patients and healthy people. Data sourced from the GEPIA website. (E) The changes of *LIG3* at the protein and mRNA levels after SKOV3 knocked down *RAD51.* Data were presented as mean±S.E. of three or four independent experiments. Significance was determined by Student’s t-test: ns, not significant; **P*<0.05, ***P*<0.01, ****P*<0.001 and *****P*<0.0001.

**Figure S3**


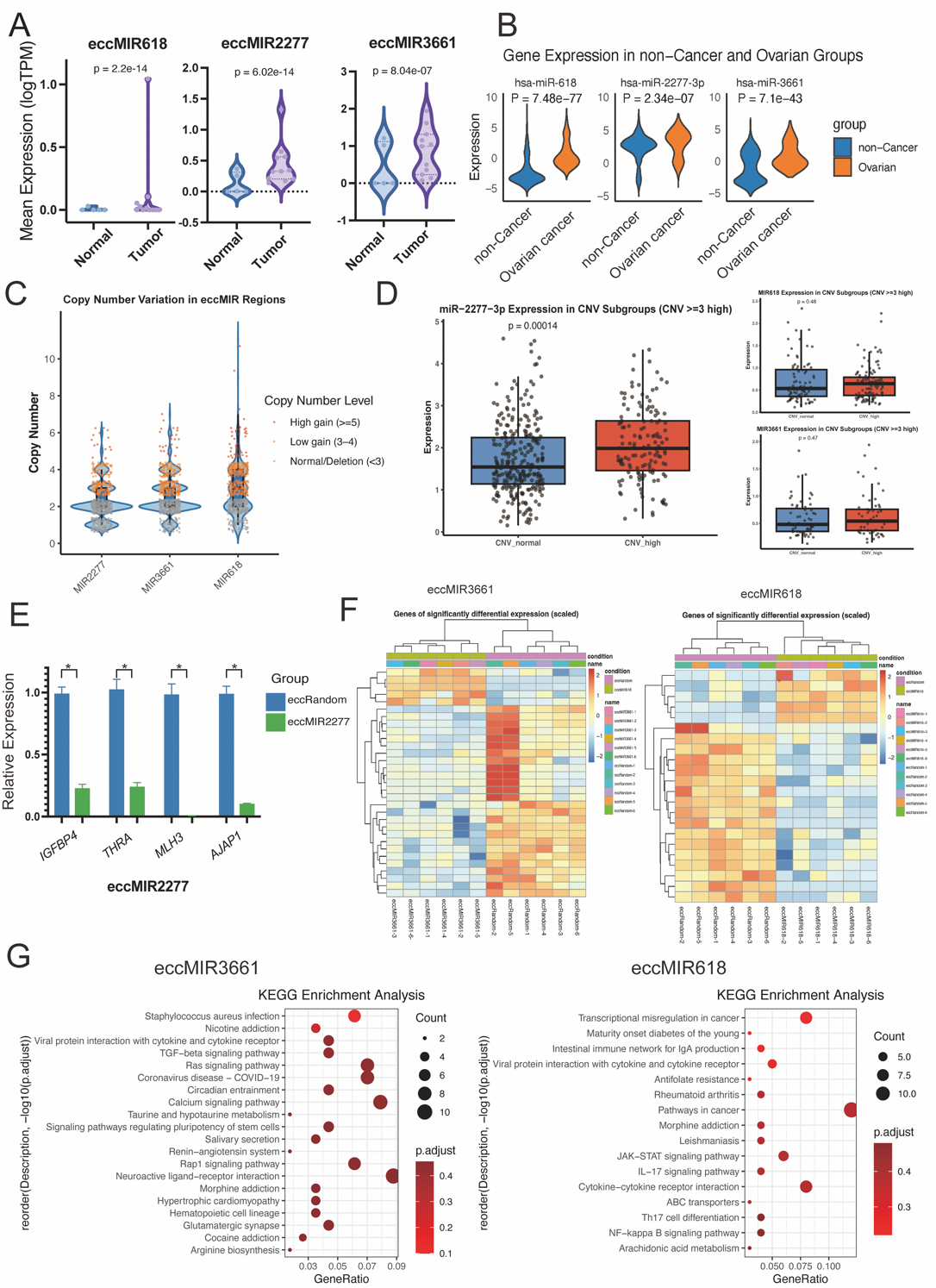


**Figure S3 Analysis of eccM3661, eccMIR618, eccMIR2277 overexpression**

**(A)** In tumor tissues versus normal tissues, the abundance of eccMIR3661, eccMIR618, and eccMIR2277 was comparatively analyzed. (B) Comparative expression profile of MIR3661, MIR618, and MIR2277 in OC patients and healthy people. Data sourced from the Gene Expression Omnibus (GEO) database (accession: GSE106817). (C) Violin plots display the distribution of copy number values for each eccMIR gene. Boxplots represent the interquartile range with the median line, while individual samples are shown as jittered dots. Dots are color-coded based on CNV levels: high-level gain (CNV >= 5, red), low-level gain (CNV = 3-4, orange), and normal/deletion (CNV < 3, gray). (D) Boxplots show the distribution of MIR3661, MIR618, and MIR2277 expression levels in samples with normal copy number (CN < 3, blue) and those with copy number gain (CN >= 3, red). The expression values are expressed as log2(RPM + 1). Individual samples are overlaid as jittered points. Statistical comparison between groups was performed using an unpaired two-sided t-test, and the p-value is indicated. (E) Down-regulated MIR2277 targets in SKOV3 cells transfected with eccMIR2277, validated by qPCR. (F) Heatmap of differentially expressed genes in SKOV3 cells transfected with eccMIR3661 and eccMIR618, respectively. (G) GO enrichment analysis of differentially expressed genes in SKOV3 cells transfected with eccMIR3661 and eccMIR618, respectively. Data were presented as mean±S.E. of three or four independent experiments. Significance was determined by Student’s t-test: ns, not significant; **P*<0.05, ***P*<0.01, ****P*<0.001 and *****P*<0.0001.

**Figure S4**

**
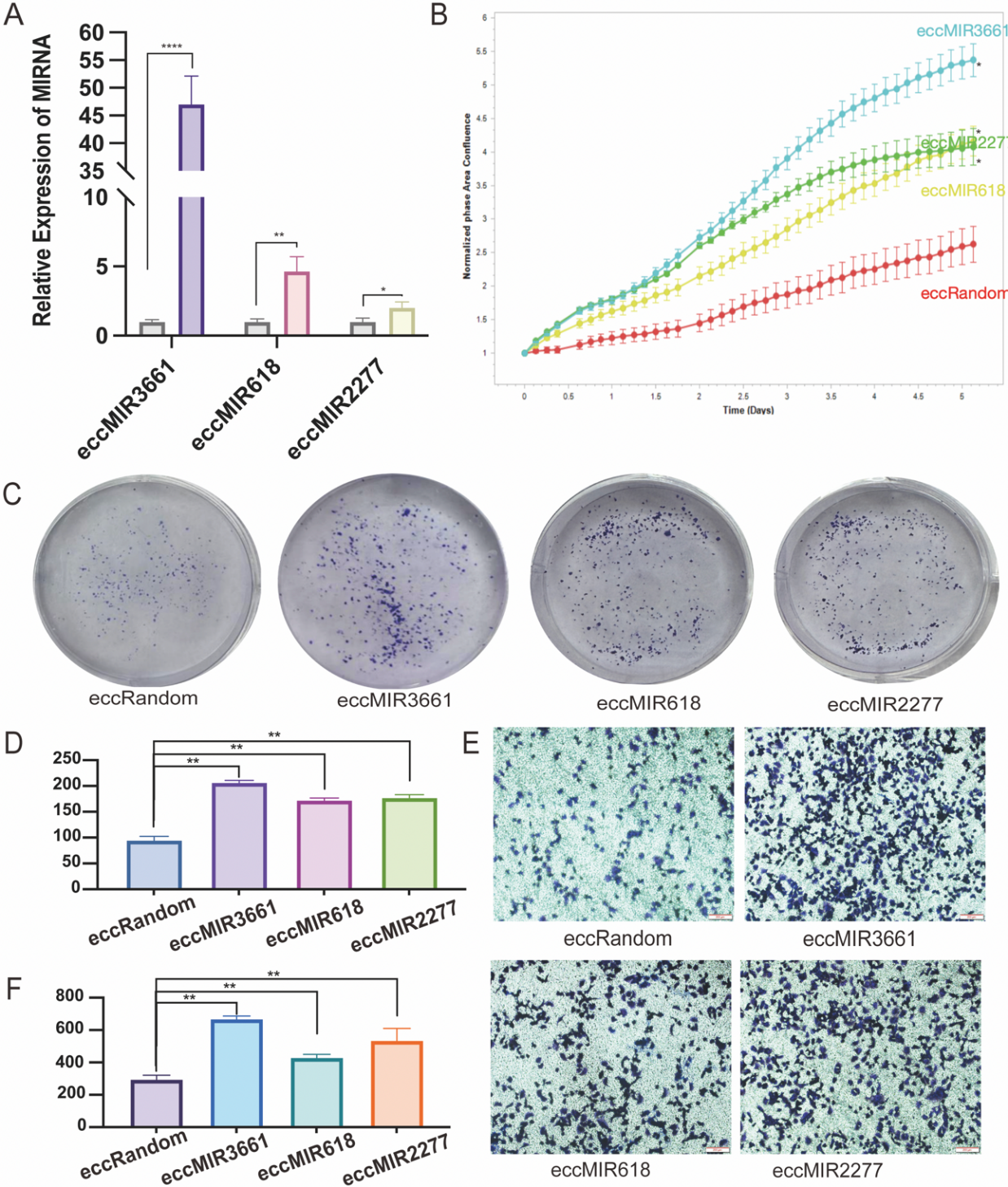
Figure S4 EccMIR promotes OVCAR8 cell migration and invasion via up-regulating the corresponding miRNA**

(A) Validation of miRNA expression by qPCR after eccMIRs transfection in OVCAR8 cells. (B) Cell viability assay demonstrating the accelerated proliferation of OVCAR8 cells after transfection with eccMIR3661, eccMIR618 and eccMIR2277. (C-F) Flat plate cloning assay and Transwell assay showing enhanced migration and invasion of OVCAR8 cells transfected with eccMIR3661, eccMIR618 and eccMIR2277. Data were presented as mean±S.E. of three or four independent experiments. Significance was determined by Student’s t-test: ns, not significant; **P*<0.05, ***P*<0.01, ****P*<0.001 and *****P*<0.0001.
