# Supplementary material for: Extrachromosomal circular DNA expressing miRNA promotes ovarian cancer progression
Source: Clin Transl Med. 2025 Sep 23;15(9):e70445. doi: 10.1002/ctm2.70445 (PMC12455017; doi:10.1002/ctm2.70445)
Supplement: Supplementary file 6 — Supporting Information [file CTM2-15-e70445-s005.docx]

**Table S1 The verified eccDNA information**

| ID | chr | start | end | eccDNA_gene | logFC | PValue | adj.PValue |
| --- | --- | --- | --- | --- | --- | --- | --- |
| ID107289 | chr12 | 80934853 | 80936003 | MIR618 | 4.877830496 | 4.61E-13 | 1.35E-12 |
| ID352281 | chr5 | 93620688 | 93623324 | MIR2277 | 2.170349951 | 1.32E-09 | 2.89E-09 |
| ID354828 | chr5 | 134225103 | 134225887 | MIR3661 | 1.361915992 | 5.01E-05 | 7.46E-05 |
