# Supplementary material for: Extrachromosomal circular DNA expressing miRNA promotes ovarian cancer progression
Source: Clin Transl Med. 2025 Sep 23;15(9):e70445. doi: 10.1002/ctm2.70445 (PMC12455017; doi:10.1002/ctm2.70445)
Supplement: Supplementary file 7 — Supporting Information [file CTM2-15-e70445-s007.docx]

**Table S2 Differentially expressed genes after overexpression of eccMIR3661 (pvalue < 0.05)**

| SYMBOL | baseMean | log2FoldChange | lfcSE | stat | pvalue |
| --- | --- | --- | --- | --- | --- |
| BAD | 28.3224608 | -2.802653064 | 0.966609831 | -2.899466749 | 0.00373798 |
| CD99 | 870.5683945 | -1.018551666 | 0.386459368 | -2.635598336 | 0.008398909 |
| ARF5 | 42.43980244 | -2.41183631 | 0.862170947 | -2.797399191 | 0.005151584 |
| RHBDD2 | 28.76629489 | -1.233159293 | 0.541765958 | -2.27618453 | 0.022834967 |
| TNFRSF12A | 3509.441818 | -3.687865612 | 1.01189796 | -3.644503457 | 0.000267909 |
| CCDC124 | 403.9241528 | -3.398301253 | 0.973538245 | -3.490670521 | 0.00048181 |
| TRAPPC6A | 38.04303368 | -1.362562373 | 0.594131673 | -2.293367674 | 0.021826846 |
| IL32 | 62.05121181 | -1.184738494 | 0.575914041 | -2.057144662 | 0.039672313 |
| TRAF3IP3 | 6.133202484 | -1.534776371 | 0.657335823 | -2.33484365 | 0.01955159 |
| UQCRC1 | 140.8312672 | -2.13876708 | 0.841041356 | -2.542998706 | 0.010990564 |
| IFFO1 | 9.828289473 | -1.41298563 | 0.511359328 | -2.763195181 | 0.005723853 |
| PRSS3 | 167.1129437 | -1.04347728 | 0.432483542 | -2.412756042 | 0.015832413 |
| FMO1 | 11.27089184 | -1.629646655 | 0.577902599 | -2.819933078 | 0.004803367 |
| LYPLA2 | 43.14117324 | -1.635093619 | 0.559875943 | -2.920457006 | 0.003495184 |
| PLAUR | 765.0535235 | -1.533752279 | 0.660802091 | -2.321046345 | 0.020284343 |
| PPP5C | 26.18535209 | -2.476841231 | 0.757431206 | -3.27005438 | 0.001075268 |
| ZC3H3 | 8.694307767 | -1.547121306 | 0.610974714 | -2.532218223 | 0.011334343 |
| CAPN1 | 57.49634555 | -1.679893047 | 0.625281338 | -2.68661952 | 0.007217912 |
| SYT13 | 10.11243022 | -1.458981671 | 0.742528344 | -1.964883472 | 0.049427726 |
| PLEKHO1 | 48.98274002 | -1.247221111 | 0.597483642 | -2.087456499 | 0.036846889 |
| EHD2 | 199.5821245 | -1.594208362 | 0.726005352 | -2.195863099 | 0.028101744 |
| GRN | 106.6831017 | -1.909587241 | 0.597344503 | -3.196793863 | 0.001389642 |
| RANBP3 | 132.7254303 | -1.549019482 | 0.724316854 | -2.13859373 | 0.032468586 |
| MAP2K3 | 29.45423565 | -1.103555843 | 0.495194404 | -2.228530519 | 0.025845159 |
| CYP46A1 | 65.08004903 | -1.318295027 | 0.531338601 | -2.481082731 | 0.013098397 |
| USP2 | 11.77006414 | -1.639519016 | 0.664842302 | -2.466026925 | 0.013662106 |
| TUBG2 | 78.60181743 | -2.160661425 | 0.814633795 | -2.652310078 | 0.007994309 |
| THAP3 | 141.3209996 | -1.607580809 | 0.548463473 | -2.931062665 | 0.003378046 |
| CAPG | 309.8307641 | -2.204358579 | 0.805122663 | -2.737916443 | 0.006182978 |
| AP2S1 | 1369.786236 | -2.521957916 | 0.779377169 | -3.235863221 | 0.001212755 |
| CYBA | 39.08090024 | -1.03536402 | 0.483389034 | -2.141885617 | 0.032202688 |
| MCF2L2 | 55.43320972 | -1.023618156 | 0.400742895 | -2.554301444 | 0.01064011 |
| THRAP3 | 3856.021951 | -1.035992113 | 0.248579186 | -4.167654297 | 3.08E-05 |
| SZRD1 | 227.8937555 | -2.457791399 | 0.80679859 | -3.046350639 | 0.002316375 |
| ISOC2 | 149.0076241 | -2.805775969 | 0.900777978 | -3.114836327 | 0.00184047 |
| RFXANK | 192.4631924 | -1.460981207 | 0.723514299 | -2.019284497 | 0.043457658 |
| HMG20B | 36.81747746 | -2.82661278 | 1.210297465 | -2.335469472 | 0.019518909 |
| SLC9A3R2 | 44.54103032 | -1.489961057 | 0.680803743 | -2.188532411 | 0.028630843 |
| NTHL1 | 52.44899558 | -1.227152117 | 0.602316569 | -2.03738728 | 0.041611248 |
| WDR18 | 83.4180182 | -2.200588322 | 0.834769922 | -2.636161491 | 0.008384982 |
| GLP2R | 5.54253137 | -1.939373316 | 0.706071446 | -2.746709739 | 0.006019638 |
| NGEF | 67.78482636 | -1.097045341 | 0.4670965 | -2.348648169 | 0.018841701 |
| ELOVL1 | 38.99312147 | -1.137026828 | 0.508779701 | -2.2348117 | 0.025429713 |
| IDH3G | 45.91352347 | -1.316859108 | 0.546877339 | -2.40796064 | 0.01604191 |
| ROGDI | 15.71006833 | -2.233242935 | 0.832486402 | -2.682617913 | 0.00730484 |
| IFI35 | 24.05416966 | -1.679612193 | 0.689334292 | -2.436571358 | 0.014827244 |
| FTSJ1 | 88.7891997 | -1.424993801 | 0.522955388 | -2.724885972 | 0.00643237 |
| SIRT2 | 45.553557 | -2.134676896 | 0.636989975 | -3.351193862 | 0.00080464 |
| BCL3 | 9.763329854 | -1.494472245 | 0.682784472 | -2.188790614 | 0.028612062 |
| FSTL3 | 110.3958494 | -1.45328799 | 0.526790546 | -2.758758677 | 0.005802136 |
| RNF126 | 93.87665248 | -2.26646922 | 0.863929751 | -2.623441568 | 0.008704637 |
| ING3 | 632.5258922 | -1.146702332 | 0.371155533 | -3.089546647 | 0.002004622 |
| ATP6AP1 | 33.57320954 | -1.240427397 | 0.495477685 | -2.503498008 | 0.012297237 |
| FAM50A | 103.3750455 | -1.321905549 | 0.516667773 | -2.558521388 | 0.010511835 |
| PRKACA | 76.26338404 | -1.506065627 | 0.469524883 | -3.207637508 | 0.001338301 |
| LIMS2 | 22.99153578 | -1.150700484 | 0.557166278 | -2.065273026 | 0.038897174 |
| HSD17B10 | 215.0877006 | -1.06453502 | 0.404756576 | -2.630062321 | 0.008536922 |
| MRPS34 | 127.6800739 | -1.151350632 | 0.484265169 | -2.377521048 | 0.017429448 |
| TEAD2 | 9.610608869 | -1.600065439 | 0.598499477 | -2.673461716 | 0.007507284 |
| BCS1L | 9.865026399 | -1.351707789 | 0.611966079 | -2.208795282 | 0.027188883 |
| ENO1 | 1567.798994 | -1.029487441 | 0.444492405 | -2.316096809 | 0.020552984 |
| MYDGF | 1687.046386 | -1.743739994 | 0.507157421 | -3.438261813 | 0.000585461 |
| MKRN2 | 57.80561786 | -1.305981851 | 0.450514762 | -2.898865835 | 0.003745151 |
| ARHGEF1 | 23.08669365 | -1.05039053 | 0.532449342 | -1.97275205 | 0.048523819 |
| SNRPA | 36.26377093 | -3.269805045 | 1.336500014 | -2.446543217 | 0.01442335 |
| ARAF | 44.59619575 | -1.801751112 | 0.49539743 | -3.636981143 | 0.000275852 |
| PGM1 | 74.18786846 | -1.289864511 | 0.448662456 | -2.874910737 | 0.004041417 |
| RPS5 | 4193.445634 | -1.071314096 | 0.42629305 | -2.513093039 | 0.011967777 |
| GSTP1 | 2205.140992 | -1.136431725 | 0.403140811 | -2.818944881 | 0.004818179 |
| AGBL5 | 33.13544091 | -1.010604001 | 0.484179453 | -2.087250906 | 0.03686546 |
| CTTN | 2207.38382 | -1.338349619 | 0.488835923 | -2.737829926 | 0.006184605 |
| B4GALT1 | 460.9491986 | -1.058443091 | 0.361760866 | -2.925808709 | 0.003435621 |
| MRPL28 | 112.6938513 | -1.562839069 | 0.56577895 | -2.76227857 | 0.005739948 |
| SRRT | 45.19666341 | -1.664267398 | 0.604700965 | -2.752215546 | 0.005919355 |
| PDRG1 | 303.327275 | -1.044688882 | 0.451965583 | -2.311434591 | 0.020808862 |
| TMEM40 | 12.62519861 | -1.675647711 | 0.63407292 | -2.642673511 | 0.008225431 |
| FXYD5 | 2769.529558 | -2.462489836 | 0.844316913 | -2.916546853 | 0.003539296 |
| GRAMD1A | 61.15571476 | -1.606621917 | 0.51505795 | -3.119303209 | 0.001812793 |
| MLF2 | 396.5794192 | -1.365495181 | 0.479649163 | -2.846862429 | 0.004415244 |
| BLVRB | 47.71419785 | -2.22872981 | 1.00055408 | -2.227495599 | 0.02591417 |
| NUDC | 3541.282569 | -1.050322314 | 0.43027793 | -2.441032277 | 0.014645346 |
| MCOLN1 | 16.47753971 | -1.771433987 | 0.743445295 | -2.382736159 | 0.017184506 |
| TBX15 | 131.5049618 | -1.034661084 | 0.458065998 | -2.258759847 | 0.023898327 |
| COMT | 78.67320322 | -1.989146167 | 0.613958358 | -3.239871466 | 0.001195836 |
| CRAT | 27.47600583 | -1.59200915 | 0.620929113 | -2.563914488 | 0.010349904 |
| NUBP2 | 145.4594925 | -2.183707646 | 0.788215948 | -2.770443368 | 0.005598003 |
| MRPS18A | 62.481678 | -1.730935345 | 0.597513448 | -2.896897718 | 0.003768726 |
| ACOT7 | 189.6028102 | -1.847935147 | 0.751957341 | -2.457499975 | 0.013990782 |
| PSMD8 | 2014.062964 | -1.446924805 | 0.523583944 | -2.763501099 | 0.00571849 |
| ATP5F1D | 176.9085403 | -2.755282347 | 0.834946123 | -3.299952263 | 0.000967013 |
| NDUFB7 | 773.6960727 | -1.546801368 | 0.497541393 | -3.10888981 | 0.001877917 |
| TECR | 277.2863644 | -2.138920362 | 0.845277576 | -2.530435472 | 0.011392104 |
| TIMM13 | 172.0051115 | -2.022543204 | 0.574155733 | -3.522638696 | 0.000427273 |
| POLR2E | 197.3798614 | -2.232831229 | 0.772493597 | -2.890420372 | 0.00384727 |
| GADD45B | 1142.728396 | -2.293159852 | 0.798853684 | -2.870563032 | 0.004097415 |
| SUSD2 | 8.252862924 | -1.471672928 | 0.700055285 | -2.102223867 | 0.03553367 |
| UPB1 | 18.16382392 | -1.229918261 | 0.524939477 | -2.342971554 | 0.019130842 |
| CCDC134 | 76.65474268 | -1.023428915 | 0.469630477 | -2.179221678 | 0.029315204 |
| CENPM | 547.0491885 | -1.766022829 | 0.682814176 | -2.58638864 | 0.00969875 |
| LMF2 | 53.04251664 | -2.202753134 | 0.82977908 | -2.654626018 | 0.007939638 |
| MCAT | 80.19393547 | -1.330435748 | 0.438517667 | -3.033938764 | 0.002413834 |
| TSPO | 117.2728184 | -1.483864508 | 0.519504271 | -2.856308585 | 0.004285984 |
| TXN2 | 516.991679 | -1.397143419 | 0.511083434 | -2.733689506 | 0.006262907 |
| PHF5A | 186.4744359 | -1.086332194 | 0.350469507 | -3.099648251 | 0.001937506 |
| POLR3H | 10.38382261 | -1.386983099 | 0.698598283 | -1.985380056 | 0.047102203 |
| TRMU | 264.8443604 | -1.226103294 | 0.429784833 | -2.852830528 | 0.004333172 |
| PMM1 | 98.15109898 | -1.975714171 | 0.755026315 | -2.616748758 | 0.008877165 |
| GSTZ1 | 80.48718535 | -1.011502997 | 0.418128491 | -2.419120005 | 0.015558107 |
| ZFYVE21 | 30.69615514 | -1.19267036 | 0.529745703 | -2.251401671 | 0.024360108 |
| NFKBIA | 871.5696723 | -1.292074143 | 0.498764885 | -2.590547535 | 0.009582339 |
| PROCR | 83.64004408 | -1.068450168 | 0.366368426 | -2.916327096 | 0.00354179 |
| C20orf27 | 93.13732522 | -3.962103025 | 1.065644967 | -3.718032881 | 0.00020078 |
| ARFRP1 | 168.4977691 | -1.440065048 | 0.560358286 | -2.569900516 | 0.010172772 |
| HM13 | 1465.017534 | -1.044307827 | 0.493507284 | -2.116094047 | 0.03433681 |
| GPR143 | 11.50404238 | -1.477421263 | 0.611695072 | -2.415290446 | 0.015722667 |
| PLP2 | 152.076492 | -2.107435917 | 0.549624805 | -3.834317336 | 0.000125914 |
| PCSK1N | 10.516496 | -2.039670038 | 0.945272523 | -2.15775873 | 0.030946595 |
| TIMP1 | 4830.559382 | -1.619266136 | 0.523924215 | -3.090649545 | 0.001997192 |
| KLHL4 | 26.18347505 | -1.124288285 | 0.457248792 | -2.458810834 | 0.013939805 |
| MAPK3 | 24.77547866 | -1.037133271 | 0.529041959 | -1.960398892 | 0.049949185 |
| NME3 | 32.23604741 | -1.764012676 | 0.691375661 | -2.551453248 | 0.010727472 |
| VAC14 | 67.15215499 | -1.109712181 | 0.529253442 | -2.09675005 | 0.036015707 |
| HCFC1R1 | 117.1473418 | -3.647865375 | 1.088286366 | -3.351935199 | 0.000802488 |
| MPG | 29.46648522 | -2.173864064 | 0.760733449 | -2.857589694 | 0.00426872 |
| STUB1 | 10.57983653 | -2.194303901 | 0.808672507 | -2.713464205 | 0.006658376 |
| ELOB | 424.7863606 | -1.406396736 | 0.435082045 | -3.232486266 | 0.00122718 |
| HMOX2 | 31.28606636 | -1.00286738 | 0.48345312 | -2.074383926 | 0.038043669 |
| QPRT | 435.0273664 | -1.401624886 | 0.42409689 | -3.304963838 | 0.000949887 |
| DNAJC17 | 220.4056339 | -1.025976578 | 0.338193621 | -3.033695834 | 0.002415779 |
| JPH1 | 33.14993675 | -1.004411024 | 0.366183922 | -2.742914047 | 0.006089662 |
| NUCB1 | 34.22446175 | -1.491452817 | 0.559753286 | -2.664482466 | 0.007710689 |
| CLPTM1 | 53.33128024 | -2.297998189 | 0.863705379 | -2.660627392 | 0.007799522 |
| PIH1D1 | 106.0949463 | -1.350925716 | 0.482528502 | -2.799680663 | 0.005115318 |
| PLEKHJ1 | 99.82385245 | -2.509084428 | 0.81174364 | -3.090981322 | 0.001994962 |
| OAZ1 | 23529.68779 | -1.16690007 | 0.475888434 | -2.452045454 | 0.014204672 |
| TLE5 | 10.39578368 | -2.143556821 | 0.852909933 | -2.513227642 | 0.011963211 |
| C19orf53 | 1491.225698 | -1.480660174 | 0.472115769 | -3.136222663 | 0.001711392 |
| TIMM50 | 468.4368473 | -1.091510904 | 0.499420743 | -2.185553801 | 0.028848266 |
| YJU2 | 37.92233121 | -1.734517299 | 0.572987811 | -3.027145198 | 0.002468753 |
| FZR1 | 22.38910624 | -1.408014334 | 0.59568875 | -2.363674544 | 0.018094701 |
| NOP53 | 41.28633078 | -1.394288824 | 0.559046738 | -2.494046973 | 0.012629583 |
| PTPRS | 111.289322 | -1.347572988 | 0.414707237 | -3.249456167 | 0.001156259 |
| TMEM205 | 22.82051348 | -1.097381229 | 0.539182729 | -2.035267766 | 0.041823938 |
| BCAT2 | 57.39295634 | -1.275040351 | 0.518018737 | -2.461378825 | 0.013840414 |
| PPP2R1A | 632.6190945 | -1.53475412 | 0.506215846 | -3.031817619 | 0.00243086 |
| TFPT | 29.90875166 | -2.321625759 | 0.683111471 | -3.39860456 | 0.000677306 |
| COPE | 141.0529146 | -3.595980149 | 1.047527803 | -3.432825496 | 0.000597326 |
| ARMC6 | 111.736594 | -1.179712257 | 0.466002605 | -2.531557218 | 0.011355729 |
| KXD1 | 202.0418425 | -2.442434248 | 0.717840986 | -3.402472546 | 0.000667791 |
| CASP2 | 373.2132347 | -1.527775338 | 0.472306864 | -3.234709154 | 0.001217667 |
| HSPB1 | 413.4801552 | -3.840536422 | 1.045720433 | -3.672622531 | 0.000240074 |
| PTCD1 | 6.583758429 | -1.414015747 | 0.653274045 | -2.16450624 | 0.030425518 |
| AIMP2 | 408.8370288 | -1.076717789 | 0.427976587 | -2.515833395 | 0.01187513 |
| IMPDH1 | 53.25076933 | -1.774452767 | 0.661927462 | -2.680735985 | 0.007346045 |
| AP1S1 | 50.45186908 | -3.247182245 | 0.695948281 | -4.665838442 | 3.07E-06 |
| ZNHIT1 | 115.8731868 | -1.254598613 | 0.420214762 | -2.985612899 | 0.002830106 |
| RARRES2 | 29.47858334 | -1.160347842 | 0.404386272 | -2.869404635 | 0.004112453 |
| POLD2 | 260.1190172 | -1.316261538 | 0.483826621 | -2.720523182 | 0.00651787 |
| EDF1 | 2185.338991 | -1.023006304 | 0.435986469 | -2.346417553 | 0.018954859 |
| NPM3 | 34.19171939 | -1.334171941 | 0.631439786 | -2.11290446 | 0.034608946 |
| FBXL15 | 14.11827441 | -2.024964494 | 0.784067716 | -2.582639805 | 0.009804763 |
| CUEDC2 | 42.82916618 | -2.125334769 | 0.915747714 | -2.320873682 | 0.020293662 |
| MTPAP | 1132.112757 | -1.160343577 | 0.283557506 | -4.092092622 | 4.27E-05 |
| RPL28 | 219.0328255 | -1.189175692 | 0.472615108 | -2.516160977 | 0.011864098 |
| GIT1 | 62.68203697 | -1.508306589 | 0.586249081 | -2.572808448 | 0.010087702 |
| UBTF | 88.39596199 | -1.443184624 | 0.534598308 | -2.69956826 | 0.006942951 |
| PFN1 | 693.0981053 | -2.235953761 | 0.837140146 | -2.670943176 | 0.007563845 |
| RNF167 | 30.51730887 | -1.397122288 | 0.50704067 | -2.755444228 | 0.00586125 |
| RAB5C | 115.3753731 | -1.817920427 | 0.529032629 | -3.43631059 | 0.000589695 |
| PPP1R9B | 26.73947007 | -1.078205515 | 0.529344713 | -2.036868395 | 0.041663232 |
| YWHAE | 7009.986681 | -1.027270153 | 0.27234259 | -3.77197761 | 0.000161959 |
| SLC9A3R1 | 140.4322611 | -1.234274533 | 0.369908078 | -3.336706076 | 0.000847776 |
| UNC119 | 96.63550138 | -1.633715456 | 0.476402293 | -3.429277062 | 0.000605191 |
| TRIM3 | 25.66678548 | -1.460189856 | 0.731912227 | -1.995034106 | 0.04603916 |
| ANAPC15 | 88.60565443 | -1.22207466 | 0.389429958 | -3.138111578 | 0.001700401 |
| FOLR3 | 23.3144022 | -1.011080242 | 0.475558261 | -2.126091219 | 0.033495665 |
| PTPMT1 | 5.580954767 | -1.37654367 | 0.61630169 | -2.233554918 | 0.025512373 |
| PITPNM1 | 23.5719616 | -1.296290908 | 0.526137541 | -2.463787143 | 0.013747773 |
| CD69 | 11.99918935 | -2.735131226 | 0.908699164 | -3.009941392 | 0.002612981 |
| GAPDH | 5550.971106 | -2.55261321 | 0.914983267 | -2.789792231 | 0.005274188 |
| B3GAT2 | 41.53292435 | -1.079710566 | 0.352883133 | -3.059683125 | 0.002215713 |
| DNPH1 | 127.8075145 | -1.53573805 | 0.439196424 | -3.496699805 | 0.000471052 |
| SLC29A1 | 111.6122537 | -1.191128464 | 0.427243833 | -2.787936006 | 0.005304502 |
| DAP | 142.3313519 | -1.257993704 | 0.391779428 | -3.210974375 | 0.001322857 |
| THBS4 | 14.89918263 | -1.011714424 | 0.507231689 | -1.994580476 | 0.046088655 |
| OGG1 | 27.08492389 | -1.294439879 | 0.503515868 | -2.570802553 | 0.010146316 |
| FGF12 | 223.1790486 | -1.042600317 | 0.26060745 | -4.000654299 | 6.32E-05 |
| TUSC2 | 116.3674604 | -1.086093321 | 0.417431631 | -2.601847201 | 0.009272315 |
| CYB561D2 | 51.99026095 | -1.551294895 | 0.494918628 | -3.134444342 | 0.001721799 |
| FAHD2A | 167.9836072 | -1.239724452 | 0.529687371 | -2.340483312 | 0.019258799 |
| NRBP1 | 186.2692964 | -1.039443442 | 0.461361123 | -2.252993131 | 0.024259582 |
| NDUFS7 | 61.57718437 | -2.736744838 | 1.008414195 | -2.713909475 | 0.006649433 |
| PCGF1 | 94.64526672 | -1.031310806 | 0.283485698 | -3.637964153 | 0.000274802 |
| GNLY | 6.143102594 | -1.259198687 | 0.563608159 | -2.234173984 | 0.025471628 |
| MAD2L2 | 278.328776 | -1.246132093 | 0.472818833 | -2.635538192 | 0.008400397 |
| PLA2G4A | 10.5195529 | -1.386269425 | 0.562286763 | -2.465413585 | 0.013685518 |
| OLFML3 | 27.07113234 | -1.439961065 | 0.352873386 | -4.080673467 | 4.49E-05 |
| PLEKHM2 | 111.7017303 | -1.158697903 | 0.552092151 | -2.09874004 | 0.035839823 |
| TMEM9 | 428.7466165 | -1.201338734 | 0.45544189 | -2.637743167 | 0.008345976 |
| ADPRS | 20.83911991 | -1.229771505 | 0.549680806 | -2.237246583 | 0.025270228 |
| ID3 | 679.5083835 | -2.445498176 | 0.728925251 | -3.354936836 | 0.000793831 |
| ATP6V0B | 5455.15499 | -1.585438986 | 0.683515586 | -2.319535967 | 0.020365993 |
| ERI3 | 98.24915376 | -3.648711683 | 1.02879395 | -3.54659131 | 0.000390249 |
| CTSD | 205.1684381 | -2.245537926 | 0.814769012 | -2.756042378 | 0.005850542 |
| COLEC11 | 8.34107053 | -1.787929738 | 0.576398179 | -3.101900392 | 0.001922826 |
| FILIP1 | 16.86811209 | -1.339334649 | 0.481355113 | -2.78242531 | 0.005395428 |
| CASQ2 | 8.470571456 | -1.221414703 | 0.589925477 | -2.070455932 | 0.038409669 |
| EEF2KMT | 49.73449218 | -1.587844105 | 0.646325407 | -2.456725494 | 0.014020978 |
| ACOT2 | 25.26812743 | -1.02151304 | 0.434253928 | -2.352340356 | 0.018655696 |
| CNNM1 | 5.598707493 | -1.616002936 | 0.780202334 | -2.071261345 | 0.03833438 |
| AVPI1 | 63.72595623 | -1.293809498 | 0.471889148 | -2.741765737 | 0.006110991 |
| CLU | 31.85676581 | -1.082011731 | 0.512350631 | -2.111857909 | 0.034698639 |
| TMEM39B | 53.46700303 | -1.267721465 | 0.525509837 | -2.412364861 | 0.015849412 |
| TMEM54 | 9.201501023 | -1.649690946 | 0.64886536 | -2.542424126 | 0.01100865 |
| RASL11A | 38.31452158 | -1.848434477 | 0.568383568 | -3.252089931 | 0.001145598 |
| FKBP9 | 455.457437 | -1.364455568 | 0.466482784 | -2.924985906 | 0.003444718 |
| CYREN | 40.68939108 | -1.324364947 | 0.426570692 | -3.104678713 | 0.001904858 |
| ACADS | 7.964809548 | -1.912061939 | 0.671247183 | -2.848521361 | 0.00439229 |
| ATG101 | 35.30032939 | -1.901381922 | 0.574187081 | -3.311432779 | 0.000928195 |
| TUBA1B | 913.7661256 | -2.450954637 | 0.845605371 | -2.898461529 | 0.003749983 |
| B9D2 | 96.85046399 | -1.445443414 | 0.597256744 | -2.42013745 | 0.015514642 |
| SDC4 | 714.5692853 | -1.437183988 | 0.457562008 | -3.140960052 | 0.00168395 |
| PAIP2B | 9.461210684 | -1.679540185 | 0.603647265 | -2.782320542 | 0.00539717 |
| USP22 | 116.0209222 | -2.079292501 | 0.75008636 | -2.772070806 | 0.005570092 |
| ZNF576 | 94.74196328 | -1.062942835 | 0.454912119 | -2.336589399 | 0.019460544 |
| BTN2A2 | 29.07482536 | -1.365118452 | 0.581111864 | -2.349149169 | 0.018816367 |
| H2BC11 | 65.09439009 | -1.011413738 | 0.362001217 | -2.793951211 | 0.005206833 |
| CDKN1A | 32.48936804 | -1.138638224 | 0.482538677 | -2.359682814 | 0.018290566 |
| MIF4GD | 139.9210547 | -1.168140665 | 0.476743493 | -2.450249837 | 0.014275712 |
| CHCHD5 | 225.0915765 | -1.619136361 | 0.472460818 | -3.427027809 | 0.000610227 |
| SLC25A23 | 37.30892052 | -1.383868201 | 0.471161062 | -2.93714467 | 0.003312495 |
| ALKBH7 | 116.8507406 | -2.046514216 | 0.798278643 | -2.563658985 | 0.010357525 |
| CLPP | 141.3334936 | -1.466869602 | 0.504138071 | -2.909658458 | 0.003618239 |
| CD70 | 89.8617156 | -1.354668007 | 0.562600665 | -2.407867768 | 0.016045992 |
| RBCK1 | 588.8656648 | -1.093391546 | 0.479680245 | -2.2794175 | 0.022642259 |
| SNRPB | 393.8741283 | -1.84480924 | 0.742378639 | -2.484997738 | 0.012955226 |
| MRPS26 | 485.6255896 | -2.09895131 | 0.774637244 | -2.709592556 | 0.006736591 |
| RALY | 124.9301968 | -1.287339247 | 0.582319659 | -2.21070889 | 0.027056002 |
| NR1D1 | 55.53323115 | -1.550271488 | 0.646693137 | -2.397228914 | 0.0165196 |
| PRDX5 | 653.7267711 | -1.241522692 | 0.464165319 | -2.67474247 | 0.007478667 |
| BCL2L12 | 111.9649119 | -1.327621002 | 0.489999193 | -2.70943508 | 0.006739789 |
| IRF3 | 53.40810285 | -1.728217885 | 0.8350408 | -2.069620891 | 0.03848786 |
| PRMT1 | 117.3255032 | -1.9196349 | 0.592872902 | -3.237852315 | 0.001204331 |
| TIMM17B | 69.52450893 | -3.146605396 | 1.239291335 | -2.539036067 | 0.011115836 |
| PZP | 8.463942025 | -1.038857334 | 0.506817372 | -2.049766623 | 0.04038721 |
| MAP2K2 | 140.295208 | -1.504793282 | 0.74439904 | -2.021487405 | 0.043229335 |
| PIN1 | 275.9275778 | -3.363383597 | 0.924664842 | -3.637408327 | 0.000275395 |
| UQCR11 | 419.2369501 | -1.075615876 | 0.43145212 | -2.493013308 | 0.01266641 |
| GFER | 22.25748087 | -1.942495006 | 0.740721269 | -2.622437194 | 0.008730336 |
| TUBA4A | 532.5277103 | -2.003746195 | 0.693357223 | -2.889918973 | 0.003853411 |
| DGCR6L | 485.8238546 | -2.141932871 | 0.810883164 | -2.641481495 | 0.008254432 |
| SDF2L1 | 232.1970561 | -1.812098988 | 0.690229892 | -2.625355711 | 0.008655849 |
| KRT17 | 18.57263007 | -1.248366709 | 0.613157567 | -2.035963961 | 0.041753974 |
| MRPS12 | 76.83940389 | -1.868427875 | 0.816282384 | -2.288947932 | 0.022082378 |
| MPDU1 | 68.24167061 | -2.130646928 | 0.634218151 | -3.359485886 | 0.000780876 |
| KLF16 | 8.404529623 | -2.174850212 | 0.769282496 | -2.827115169 | 0.004696943 |
| ECSIT | 63.62532959 | -1.462274638 | 0.566290809 | -2.582197369 | 0.009817342 |
| ELOF1 | 59.6189966 | -1.735389429 | 0.523457443 | -3.315244542 | 0.00091563 |
| BST2 | 93.79507245 | -1.852788339 | 0.541798629 | -3.419699205 | 0.000626904 |
| DDA1 | 99.14276004 | -2.546419836 | 0.778580395 | -3.270593316 | 0.001073221 |
| PGLS | 263.9730219 | -1.859123538 | 0.785734618 | -2.366096002 | 0.017976782 |
| ACTN4 | 190.1944918 | -1.240242189 | 0.518294947 | -2.392927419 | 0.016714547 |
| LSM4 | 1740.798953 | -1.12437274 | 0.500477731 | -2.246598939 | 0.02466567 |
| ADRM1 | 90.79508822 | -1.1693966 | 0.594714561 | -1.966315738 | 0.04926215 |
| UBE2M | 137.1021791 | -2.106197014 | 0.786101619 | -2.679293572 | 0.007377768 |
| TRIM28 | 202.5665657 | -1.450851145 | 0.708348517 | -2.048216536 | 0.040538787 |
| METTL26 | 286.5945702 | -1.866387782 | 0.80112354 | -2.329712819 | 0.019821335 |
| YIPF2 | 12.74646067 | -1.938291566 | 0.880441629 | -2.201499228 | 0.027700701 |
| EIF3G | 431.5658898 | -1.939645521 | 0.811446597 | -2.39035511 | 0.016832089 |
| ULBP3 | 344.4896582 | -1.297176227 | 0.44295908 | -2.928433541 | 0.003406747 |
| TUBG1 | 514.0123326 | -2.488314485 | 0.892305572 | -2.788634929 | 0.005293069 |
| TRAF7 | 22.18549224 | -1.863325449 | 0.547833381 | -3.401263072 | 0.000670752 |
| NINJ1 | 177.9427058 | -2.67513145 | 0.900928952 | -2.969303457 | 0.002984757 |
| LGALS3 | 1105.716639 | -1.278448424 | 0.448621004 | -2.849729312 | 0.004375645 |
| ARFIP2 | 179.4702314 | -1.079667403 | 0.460616926 | -2.343959464 | 0.019080245 |
| PER2 | 739.2458797 | -1.107149288 | 0.316118552 | -3.502323039 | 0.00046122 |
| EIF5A | 256.9103829 | -2.296581002 | 0.720118083 | -3.1891728 | 0.001426806 |
| FLOT2 | 97.61616767 | -1.694160808 | 0.747291978 | -2.267066766 | 0.023386147 |
| LOXL2 | 426.686417 | -1.003988448 | 0.399365574 | -2.513958422 | 0.011938451 |
| RTL8C | 245.5439375 | -1.29161743 | 0.502210877 | -2.571862715 | 0.010115299 |
| ELF5 | 11.64096536 | -1.542292316 | 0.652019115 | -2.365409666 | 0.018010136 |
| CDK4 | 73.96601166 | -2.095367432 | 0.846800517 | -2.474452235 | 0.013344066 |
| DPM2 | 1199.249263 | -2.249514171 | 0.850109644 | -2.646145925 | 0.008141469 |
| GRHPR | 199.4940761 | -1.135664173 | 0.356506782 | -3.185533151 | 0.001444875 |
| CNPY3 | 129.2667584 | -1.910467221 | 0.594620043 | -3.212920997 | 0.001313924 |
| FOXP4 | 18.08910088 | -1.063017189 | 0.505647205 | -2.102290249 | 0.035527858 |
| GCM1 | 14.92503329 | -1.233394007 | 0.487179501 | -2.531703417 | 0.011350996 |
| HMGA1 | 432.6871321 | -1.426259987 | 0.496137073 | -2.874729716 | 0.004043735 |
| RNF144B | 80.44839596 | -1.67834071 | 0.421344964 | -3.983293624 | 6.80E-05 |
| SLC44A5 | 12.22161801 | -1.092661703 | 0.438191049 | -2.493573762 | 0.012646431 |
| UBL7 | 131.2774804 | -1.535634309 | 0.512489794 | -2.996419302 | 0.002731705 |
| MTTP | 12.28348163 | -1.132171373 | 0.518226634 | -2.184703175 | 0.028910617 |
| DHH | 12.64136653 | -1.034214505 | 0.503984814 | -2.052074737 | 0.0401624 |
| VPS37B | 20.28938328 | -1.667940385 | 0.686088564 | -2.431086121 | 0.015053636 |
| TGFB1I1 | 46.42795091 | -1.626394358 | 0.638851041 | -2.545811547 | 0.010902403 |
| NUDT7 | 12.22230649 | -1.471137728 | 0.547403095 | -2.687485223 | 0.007199229 |
| NOB1 | 101.5928864 | -1.030845668 | 0.498229581 | -2.069017389 | 0.038544455 |
| TOB1 | 397.4959549 | -1.70899396 | 0.382087702 | -4.472779286 | 7.72E-06 |
| LRRC46 | 20.96294265 | -1.549119025 | 0.651996764 | -2.375961218 | 0.017503302 |
| SCRN2 | 25.30634264 | -1.006761307 | 0.51326072 | -1.961500789 | 0.049820633 |
| G6PC3 | 85.67626931 | -1.839377726 | 0.600481109 | -3.063173344 | 0.002190032 |
| PELP1 | 41.62749852 | -1.179448368 | 0.527237115 | -2.237035928 | 0.025283992 |
| NARF | 349.8808211 | -1.410741876 | 0.536007044 | -2.631946525 | 0.008489722 |
| CBX4 | 10.83659241 | -1.049972979 | 0.499605363 | -2.101604702 | 0.035587918 |
| PFKL | 117.2571347 | -2.148238039 | 0.602644219 | -3.564687044 | 0.000364291 |
| SH3GL1 | 30.94332043 | -1.631155642 | 0.658005019 | -2.478941034 | 0.013177309 |
| IFITM3 | 1120.543653 | -1.318172177 | 0.447812336 | -2.943581654 | 0.003244382 |
| TRPM2 | 34.09461939 | -1.011562696 | 0.484160468 | -2.089312869 | 0.036679569 |
| EMP3 | 391.2789366 | -1.646350923 | 0.486954865 | -3.38091072 | 0.00072246 |
| NOSIP | 1050.313466 | -2.178756986 | 0.839285889 | -2.595965231 | 0.009432561 |
| SLC2A5 | 14.95256949 | -1.314448058 | 0.538100839 | -2.442754152 | 0.014575663 |
| SH3BGRL3 | 1778.116322 | -2.82726344 | 0.894640596 | -3.16022261 | 0.001576486 |
| FNDC7 | 7.786361369 | -1.103171199 | 0.523679994 | -2.106575031 | 0.035154433 |
| METTL25B | 16.73847137 | -1.750041823 | 0.763985132 | -2.290675237 | 0.021982204 |
| SF3B4 | 48.98448381 | -2.523808974 | 0.949834282 | -2.657104531 | 0.0078815 |
| MRPL9 | 248.065795 | -1.145981309 | 0.437260323 | -2.620821621 | 0.008771815 |
| GUK1 | 1336.779416 | -2.123677426 | 0.78434899 | -2.707566981 | 0.006777839 |
| RABL2A | 19.98231366 | -1.127963355 | 0.418541669 | -2.694984606 | 0.007039188 |
| HES6 | 28.2645287 | -1.157957778 | 0.492665925 | -2.35039145 | 0.018753677 |
| MARCHF4 | 9.183159895 | -1.319127188 | 0.584354135 | -2.257410549 | 0.023982433 |
| MED12L | 76.27981232 | -1.055347851 | 0.332694676 | -3.172121245 | 0.001513298 |
| NHP2 | 334.2298498 | -1.283046904 | 0.492464842 | -2.605357369 | 0.009177848 |
| FARS2 | 114.759997 | -1.008321994 | 0.448907224 | -2.246170122 | 0.024693113 |
| CYP39A1 | 53.3726216 | -1.041675441 | 0.361316174 | -2.883002521 | 0.003939043 |
| SCML4 | 16.72336034 | -1.665384458 | 0.619696014 | -2.687421607 | 0.0072006 |
| SLC2A12 | 6.743171038 | -2.771259023 | 0.796406714 | -3.479703238 | 0.000501969 |
| VIP | 6.55028395 | -1.902850376 | 0.758966824 | -2.507158832 | 0.012170601 |
| C7orf50 | 162.8282747 | -3.133642421 | 0.994189252 | -3.151957652 | 0.001621798 |
| AWAT2 | 11.63532152 | -2.2115097 | 0.880029715 | -2.512994346 | 0.011971125 |
| BIN3 | 125.4222536 | -1.233548961 | 0.488824255 | -2.523501951 | 0.011619242 |
| SLC39A4 | 84.40530637 | -1.088361697 | 0.517835937 | -2.101750031 | 0.035575178 |
| SIGMAR1 | 296.6989415 | -1.118051313 | 0.465332509 | -2.402693324 | 0.01627483 |
| NTMT1 | 177.6962794 | -2.129289949 | 0.795005691 | -2.678332966 | 0.007398963 |
| CIZ1 | 109.0867687 | -1.763177425 | 0.588940008 | -2.99381499 | 0.00275513 |
| SH3GLB2 | 58.37716058 | -1.192409303 | 0.553228405 | -2.155365291 | 0.03113326 |
| PAXX | 87.01715949 | -1.563547829 | 0.794061357 | -1.969051653 | 0.04894716 |
| A1CF | 43.31567538 | -1.248912223 | 0.32393753 | -3.855410711 | 0.000115535 |
| ADM | 213.9850981 | -1.109045127 | 0.435586166 | -2.546098139 | 0.010893456 |
| LAMTOR1 | 179.3355009 | -1.1115451 | 0.314056762 | -3.539312742 | 0.00040117 |
| OOSP2 | 6.896338603 | -1.575595894 | 0.587382439 | -2.682402111 | 0.007309554 |
| TAGLN | 84.44405362 | -2.406755 | 0.8419068 | -2.858695287 | 0.004253872 |
| TRPT1 | 8.352434326 | -1.726624918 | 0.758432457 | -2.276570446 | 0.022811889 |
| MRPL49 | 127.7093931 | -1.100299063 | 0.45280458 | -2.429964521 | 0.015100301 |
| TMEM219 | 861.5226265 | -1.24583149 | 0.449248921 | -2.773142978 | 0.005551772 |
| PIP4K2A | 247.386498 | -1.100436545 | 0.346218441 | -3.178445787 | 0.001480669 |
| DRD3 | 9.711277213 | -1.459676896 | 0.731575443 | -1.995251357 | 0.046015472 |
| TMEM178A | 7.90892219 | -2.250393617 | 0.74091267 | -3.037326407 | 0.002386868 |
| NMRAL1 | 116.2699917 | -1.514891376 | 0.591977382 | -2.559035905 | 0.01049629 |
| C16orf74 | 68.77882755 | -1.317388125 | 0.525508278 | -2.506883678 | 0.012180079 |
| C10orf90 | 61.31383599 | -1.010257404 | 0.326495476 | -3.094246258 | 0.001973136 |
| RHOC | 857.6361588 | -2.581030149 | 0.808666763 | -3.191710441 | 0.00141433 |
| MED19 | 82.60580378 | -1.661304083 | 0.458285471 | -3.62504201 | 0.000288914 |
| MS4A1 | 18.74175091 | -1.44359609 | 0.571115652 | -2.527677334 | 0.011481982 |
| DHRS4 | 102.8595154 | -1.22424164 | 0.533904604 | -2.292996971 | 0.021848179 |
| KCNJ6 | 37.13398654 | -1.30370755 | 0.430018833 | -3.031745241 | 0.002431443 |
| UBXN11 | 27.89628875 | -1.533467021 | 0.553342237 | -2.771281348 | 0.005583616 |
| TSPAN33 | 8.450430844 | -1.349942411 | 0.659757806 | -2.046118134 | 0.040744749 |
| TAGLN2 | 2896.768567 | -1.349423122 | 0.49927763 | -2.702751013 | 0.006876824 |
| DUSP23 | 174.8809636 | -3.447674214 | 0.962905945 | -3.580489073 | 0.000342952 |
| VWA5B1 | 12.66477029 | -1.306736598 | 0.581853088 | -2.245818791 | 0.024715617 |
| NDUFS2 | 133.9151818 | -1.282855811 | 0.490147 | -2.617287898 | 0.008863155 |
| FBXW5 | 38.265687 | -2.346184799 | 0.946583387 | -2.478582268 | 0.013190569 |
| CCDC24 | 30.4580431 | -1.464790854 | 0.715924204 | -2.046013873 | 0.040755006 |
| CBR3 | 19.93161116 | -1.305310936 | 0.429214678 | -3.041160992 | 0.002356678 |
| CLDN14 | 18.79086764 | -1.339139529 | 0.620958682 | -2.156567847 | 0.031039352 |
| ATP13A2 | 17.55403232 | -1.574428942 | 0.581155061 | -2.709137455 | 0.006745839 |
| ISL2 | 5.590184765 | -1.640037464 | 0.661423092 | -2.479558825 | 0.013154503 |
| ZDHHC1 | 11.95489611 | -1.168460636 | 0.558789016 | -2.091058706 | 0.036522802 |
| ATP6V0D1 | 106.7475341 | -2.262600546 | 0.786525647 | -2.876702818 | 0.004018539 |
| ZYX | 41.14704161 | -3.417395925 | 1.136140223 | -3.007899779 | 0.002630599 |
| IQCC | 19.28743975 | -1.098129902 | 0.365461633 | -3.004774792 | 0.002657776 |
| TMEM234 | 52.42064418 | -1.00388013 | 0.443586526 | -2.263098791 | 0.0236296 |
| UBE2J2 | 177.4654237 | -1.893261836 | 0.730327078 | -2.592347856 | 0.009532333 |
| G6PD | 57.62103629 | -1.570892013 | 0.608667487 | -2.580870582 | 0.009855152 |
| SLX9 | 150.7090494 | -1.989035773 | 0.725989836 | -2.739757053 | 0.006148461 |
| FTCD | 89.85734632 | -1.211023635 | 0.597513782 | -2.02677105 | 0.042685832 |
| PIERCE1 | 13.20719915 | -1.244878241 | 0.546511134 | -2.277864371 | 0.02273466 |
| TOR2A | 25.14715339 | -1.446993316 | 0.727232302 | -1.989726407 | 0.046621082 |
| SHKBP1 | 111.958445 | -2.095718994 | 0.854211451 | -2.453396044 | 0.014151444 |
| ZDHHC12 | 35.47177782 | -2.090705096 | 0.626229701 | -3.338559465 | 0.00084214 |
| MED27 | 178.5746888 | -1.085807987 | 0.396904011 | -2.735694163 | 0.006224885 |
| PMF1 | 162.3142217 | -2.299673221 | 0.583694613 | -3.939856853 | 8.15E-05 |
| PPP1R35 | 8.995576028 | -2.160277488 | 0.747943979 | -2.888287824 | 0.003873453 |
| LY6E | 21.25693561 | -2.066482741 | 0.628426997 | -3.288341767 | 0.001007794 |
| VPS28 | 259.3372014 | -1.592887624 | 0.751697732 | -2.119053384 | 0.034085956 |
| SQSTM1 | 254.3206448 | -1.887303541 | 0.752648793 | -2.507548752 | 0.012157181 |
| RPL8 | 259.2146185 | -1.236715713 | 0.492520086 | -2.510995488 | 0.012039124 |
| YDJC | 21.31251033 | -1.553116234 | 0.661346175 | -2.348416447 | 0.018853429 |
| AP2M1 | 929.5316382 | -2.663187224 | 0.835728125 | -3.186666984 | 0.001439224 |
| U2AF1L4 | 12.56746708 | -2.577635468 | 0.793984477 | -3.246455749 | 0.001168516 |
| THAP8 | 13.23097356 | -1.779839637 | 0.820841703 | -2.168310444 | 0.030135074 |
| JOSD2 | 18.69356624 | -2.358885319 | 0.834761089 | -2.825820885 | 0.004715963 |
| IP6K3 | 6.760593037 | -2.469669705 | 0.947765761 | -2.605780674 | 0.009166514 |
| FLYWCH2 | 182.5907251 | -2.107722653 | 0.860903102 | -2.44826932 | 0.014354431 |
| RPL29 | 1404.512337 | -2.562019915 | 0.758584482 | -3.377369268 | 0.000731827 |
| ITIH3 | 9.637901886 | -1.009930639 | 0.515013317 | -1.960979658 | 0.049881396 |
| SELENON | 65.59221765 | -1.570307838 | 0.571631714 | -2.747062136 | 0.006013174 |
| SLC25A34 | 11.85323039 | -1.185455764 | 0.559426854 | -2.119054092 | 0.034085896 |
| DHRS3 | 14.91256519 | -1.777663488 | 0.743812247 | -2.389935761 | 0.01685132 |
| KLHDC9 | 44.37424806 | -1.438775282 | 0.568596934 | -2.530395779 | 0.011393393 |
| MRPL55 | 69.02213125 | -1.067437561 | 0.499653952 | -2.136353682 | 0.032650596 |
| PMVK | 22.14467548 | -1.939651439 | 0.714146644 | -2.716040824 | 0.006606777 |
| LMOD3 | 16.70158802 | -1.081915351 | 0.484158062 | -2.234632522 | 0.025441484 |
| NAXE | 607.3519727 | -1.49249773 | 0.505155429 | -2.954531706 | 0.00313144 |
| RNF25 | 17.82990669 | -1.79014651 | 0.707292506 | -2.530984699 | 0.011374281 |
| EFHB | 7.274989308 | -2.14106093 | 0.667379646 | -3.208160366 | 0.00133587 |
| CXCL3 | 226.4898063 | -1.371904122 | 0.398709768 | -3.440859071 | 0.00057987 |
| ATRIP | 16.03136027 | -1.189850809 | 0.483361664 | -2.461616009 | 0.013831266 |
| ATRIP-TREX1 | 16.03136027 | -1.189850809 | 0.483361664 | -2.461616009 | 0.013831266 |
| TEX264 | 196.3344555 | -1.384536767 | 0.501212871 | -2.762372729 | 0.005738293 |
| DUSP7 | 123.0999224 | -1.084895589 | 0.512530356 | -2.116744064 | 0.034281575 |
| BMT2 | 110.4015864 | -1.029098294 | 0.462201832 | -2.226512797 | 0.025979853 |
| IQUB | 25.60491575 | -1.032808839 | 0.316137099 | -3.266965007 | 0.001087071 |
| CDK5 | 33.69272606 | -1.498592297 | 0.61296478 | -2.444826108 | 0.0144922 |
| RPP25L | 126.163376 | -1.665005222 | 0.578678608 | -2.877253798 | 0.004011528 |
| CARD19 | 52.01308295 | -1.220372317 | 0.59060185 | -2.066319835 | 0.03879829 |
| STOML2 | 2174.837687 | -1.314483175 | 0.492947228 | -2.666579911 | 0.007662739 |
| COMTD1 | 15.47627998 | -1.713952829 | 0.546555564 | -3.135916901 | 0.001713177 |
| PIP4P1 | 49.48739208 | -1.133735583 | 0.489637431 | -2.315459381 | 0.020587805 |
| PSMC3 | 909.4042756 | -1.229649822 | 0.516823894 | -2.379243369 | 0.017348218 |
| ANPEP | 117.8233012 | -1.093171959 | 0.504558065 | -2.166592977 | 0.030265903 |
| PLK1 | 23.34112917 | -1.396221202 | 0.581357731 | -2.401655862 | 0.016321055 |
| GREM1 | 16.95554526 | -1.233734598 | 0.447889792 | -2.754549493 | 0.0058773 |
| NKX3-1 | 554.8981495 | -1.214272416 | 0.383439207 | -3.166792527 | 0.001541302 |
| PHB1 | 773.549894 | -1.150420032 | 0.495204222 | -2.323122425 | 0.020172577 |
| TMEM92 | 14.23396494 | -1.521777532 | 0.703120215 | -2.164320554 | 0.030439756 |
| SLC27A4 | 80.84964389 | -1.63984955 | 0.763352049 | -2.148221847 | 0.031696136 |
| PRRX2 | 7.052792947 | -1.733779686 | 0.731413138 | -2.370451932 | 0.017766354 |
| RAB8A | 285.5362924 | -1.496817977 | 0.498072306 | -3.005222254 | 0.002653869 |
| GPX4 | 1823.511947 | -3.546240883 | 0.934259734 | -3.795776222 | 0.000147182 |
| TUBA1A | 601.8362173 | -1.11256265 | 0.423089975 | -2.629612411 | 0.008548227 |
| TUBA1C | 1361.286941 | -1.718398426 | 0.731120493 | -2.350362822 | 0.018755119 |
| AXL | 26567.7227 | -1.301195287 | 0.423610997 | -3.07167495 | 0.002128614 |
| YIF1B | 61.87083066 | -2.0836709 | 0.618288554 | -3.370062226 | 0.000751512 |
| EEF2 | 383.9142069 | -1.556810241 | 0.675562435 | -2.304465375 | 0.021196534 |
| HSD11B1L | 35.50169732 | -1.520823954 | 0.543555042 | -2.797920795 | 0.005143272 |
| C19orf48 | 49.40743365 | -2.43036228 | 0.65528086 | -3.708886417 | 0.000208173 |
| OTUB1 | 426.2337075 | -1.824632495 | 0.536658318 | -3.399989219 | 0.000673885 |
| CD320 | 113.5979589 | -2.794018138 | 0.923363807 | -3.025912557 | 0.00247884 |
| SPRYD3 | 48.75588617 | -1.504618972 | 0.559043535 | -2.691416461 | 0.007114931 |
| IGFBP6 | 560.5379016 | -2.76440225 | 0.811662977 | -3.405849877 | 0.000659584 |
| NDUFV1 | 70.52725262 | -1.172912246 | 0.50802024 | -2.308790388 | 0.020955216 |
| CDK2AP2 | 1055.114139 | -2.907392904 | 0.932534171 | -3.117733372 | 0.001822476 |
| NUDT8 | 16.23010283 | -2.469778752 | 0.746532346 | -3.308334547 | 0.000938526 |
| PRDX2 | 117.8170398 | -3.047105956 | 0.869913672 | -3.50276821 | 0.00046045 |
| CD300C | 8.999892718 | -1.514940141 | 0.746196578 | -2.030215878 | 0.042334601 |
| TK1 | 502.1947151 | -1.566962345 | 0.541537002 | -2.893546221 | 0.003809182 |
| GHDC | 24.05592256 | -1.009366541 | 0.501758006 | -2.011660063 | 0.044255783 |
| FTH1 | 13474.00067 | -4.168558933 | 1.050137895 | -3.969534812 | 7.20E-05 |
| COPS6 | 338.0200172 | -1.183001222 | 0.448575223 | -2.637241565 | 0.008358329 |
| TTC39C | 584.5397054 | -1.632336796 | 0.426255935 | -3.829475823 | 0.000128416 |
| H1-4 | 77.56106022 | -1.382803537 | 0.610205777 | -2.266126591 | 0.023443634 |
| DTYMK | 1639.620216 | -1.647771395 | 0.684441025 | -2.407470233 | 0.016063472 |
| WFDC13 | 9.990534006 | -2.321979674 | 0.923141032 | -2.51530329 | 0.011893002 |
| PKIG | 129.9887469 | -1.502994042 | 0.538378657 | -2.791704357 | 0.005243124 |
| CXXC4 | 28.64357757 | -1.706725135 | 0.668714897 | -2.55224632 | 0.010703082 |
| DHRSX | 277.6109005 | -1.408207984 | 0.474217573 | -2.969539857 | 0.002982461 |
| TBC1D10B | 41.7416663 | -1.116542683 | 0.392918216 | -2.841666888 | 0.004487836 |
| LMAN2 | 544.9813621 | -1.295098725 | 0.487353413 | -2.657411831 | 0.007874318 |
| PRELID1 | 14.18234636 | -2.969715061 | 0.92614136 | -3.206546204 | 0.001343387 |
| EFNA1 | 98.3581926 | -2.429840669 | 0.776670569 | -3.128534499 | 0.001756804 |
| SHE | 17.01939663 | -1.251120982 | 0.476988637 | -2.622957625 | 0.008717011 |
| SNUPN | 95.36721606 | -1.01239897 | 0.47448539 | -2.133677856 | 0.03286916 |
| CENPX | 332.2414378 | -1.035211167 | 0.378873931 | -2.732336755 | 0.006288683 |
| AGPAT2 | 52.72998302 | -1.416145574 | 0.665634225 | -2.127513163 | 0.033377469 |
| GPS1 | 244.9902752 | -1.764475553 | 0.713844202 | -2.471793632 | 0.01344371 |
| DCXR | 208.3198534 | -1.748386618 | 0.735385356 | -2.377510788 | 0.017429933 |
| MUC17 | 6.583619893 | -3.151690012 | 0.933867203 | -3.374880284 | 0.000738478 |
| TMEM42 | 67.95156877 | -1.028993959 | 0.519358593 | -1.981278393 | 0.047560062 |
| TRAPPC1 | 56.16497585 | -2.53718381 | 0.813381256 | -3.119304497 | 0.001812785 |
| COMMD5 | 106.126078 | -1.626628451 | 0.577350567 | -2.817401669 | 0.004841394 |
| CAVIN3 | 118.9469784 | -1.982622675 | 0.781183545 | -2.53797291 | 0.011149661 |
| FAM86JP | 15.32819068 | -1.206827077 | 0.510399822 | -2.364473936 | 0.018055698 |
| BBLN | 124.6311646 | -3.946477434 | 1.015878792 | -3.884791634 | 0.000102418 |
| SCAND1 | 484.2785817 | -2.549686171 | 0.911831179 | -2.796226134 | 0.005170321 |
| FAM241B | 119.9249482 | -1.00191275 | 0.432847127 | -2.314703477 | 0.020629165 |
| KRT19 | 36.43236656 | -1.001531881 | 0.487187209 | -2.055743382 | 0.039807258 |
| LURAP1 | 7.879735087 | -1.1568374 | 0.535038801 | -2.162156086 | 0.030606147 |
| DLK2 | 6.455868181 | -1.583633513 | 0.636200579 | -2.489204767 | 0.01280292 |
| PTEN | 1971.3895 | -1.28822233 | 0.400875579 | -3.213521597 | 0.001311179 |
| MBOAT1 | 51.44161389 | -1.154506728 | 0.41720119 | -2.767266142 | 0.005652859 |
| BSG | 757.6604218 | -1.754072731 | 0.769442868 | -2.279665981 | 0.022627506 |
| POP7 | 231.2448802 | -1.400875938 | 0.55760118 | -2.512325994 | 0.011993824 |
| GNB2 | 156.2169911 | -2.061696963 | 0.74898891 | -2.752640175 | 0.005911683 |
| PPP1CA | 303.2420057 | -3.637622724 | 1.014645445 | -3.585117089 | 0.000336927 |
| TMEM134 | 114.0548963 | -1.984048006 | 0.788164941 | -2.517300509 | 0.011825792 |
| CORO1B | 15.79477129 | -1.617676466 | 0.718101154 | -2.252713921 | 0.024277193 |
| CFL1 | 9692.306622 | -4.956800492 | 1.128182739 | -4.3936149 | 1.11E-05 |
| C11orf86 | 6.477351431 | -2.172529102 | 0.987190133 | -2.200720032 | 0.02775585 |
| RCE1 | 51.56537304 | -2.0617405 | 0.597861285 | -3.448526526 | 0.000563654 |
| PHLDA3 | 25.2131986 | -1.56025774 | 0.598405638 | -2.607358021 | 0.00912439 |
| SNHG11 | 96.6291018 | -1.131816997 | 0.437375906 | -2.587744274 | 0.009660667 |
| YIF1A | 59.4391392 | -2.282651155 | 0.848297747 | -2.690860802 | 0.007126792 |
| RAB1B | 14.32502639 | -2.887514565 | 1.30172809 | -2.218216375 | 0.026540083 |
| MED16 | 45.12472322 | -2.005722455 | 0.641453363 | -3.12684066 | 0.001766957 |
| DRAP1 | 303.8311005 | -4.831574114 | 1.063445097 | -4.543322573 | 5.54E-06 |
| UCP2 | 10.47503739 | -1.092088229 | 0.49550586 | -2.203986504 | 0.027525293 |
| C11orf68 | 67.8697356 | -1.143896146 | 0.532162928 | -2.149522424 | 0.03159301 |
| CCDC85B | 75.96643672 | -2.063430072 | 0.846284302 | -2.438223263 | 0.014759655 |
| B3GNTL1 | 44.34680691 | -1.056668874 | 0.457859313 | -2.307846195 | 0.021007693 |
| AURKAIP1 | 203.1527738 | -2.12161498 | 0.805545278 | -2.633762543 | 0.008444452 |
| SFN | 13.52422787 | -2.759928186 | 0.800171992 | -3.449168695 | 0.000562315 |
| ARL4D | 152.5646075 | -1.052176176 | 0.457530092 | -2.299687374 | 0.021465938 |
| NUPR1 | 74.85368203 | -1.622657204 | 0.628516245 | -2.58172675 | 0.009830739 |
| SSNA1 | 285.4189459 | -1.647187216 | 0.744519739 | -2.212415778 | 0.02693795 |
| WDR25 | 18.25707829 | -1.036966359 | 0.414655527 | -2.500789909 | 0.012391666 |
| SGF29 | 136.8911214 | -3.00496833 | 0.936345803 | -3.209250601 | 0.001330814 |
| BOK | 66.27427383 | -1.424940357 | 0.511851959 | -2.78389158 | 0.005371098 |
| TYMS | 58.70255926 | -1.325065977 | 0.443544774 | -2.987445808 | 0.002813192 |
| RHOG | 299.2917055 | -1.850440934 | 0.683209529 | -2.708453051 | 0.006759768 |
| EPS8L2 | 25.44030735 | -1.236990596 | 0.584967931 | -2.114629763 | 0.034461515 |
| SLC25A22 | 20.37459341 | -1.093697158 | 0.52322299 | -2.090307917 | 0.036590149 |
| PNPLA2 | 23.83957085 | -1.557894236 | 0.648013453 | -2.404107859 | 0.016211989 |
| AGTRAP | 63.73270594 | -1.32446708 | 0.555471562 | -2.384401237 | 0.017106939 |
| CD151 | 98.38685521 | -1.597284049 | 0.504853956 | -3.163853685 | 0.00155695 |
| POLR2L | 410.1661727 | -1.496398913 | 0.450243902 | -3.32352955 | 0.00088886 |
| TMEM94 | 33.70021303 | -1.206774827 | 0.51996883 | -2.320859938 | 0.020294404 |
| CHID1 | 69.55349496 | -1.910521692 | 0.638569948 | -2.991875358 | 0.002772694 |
| BOLA1 | 124.9928379 | -1.895551447 | 0.552048551 | -3.433668007 | 0.000595473 |
| PLEC | 232.3477583 | -1.620928749 | 0.723822575 | -2.239400657 | 0.025129859 |
| GRINA | 16.1380396 | -1.802919718 | 0.782698824 | -2.303465476 | 0.021252667 |
| LMO7DN | 6.522192142 | -3.362152856 | 0.753511612 | -4.461978822 | 8.12E-06 |
| CD300LB | 67.99983412 | -1.269333739 | 0.492744061 | -2.57605081 | 0.009993596 |
| EXOSC4 | 90.88589431 | -2.03057559 | 0.608987727 | -3.334345666 | 0.000855003 |
| CYBC1 | 36.50311288 | -1.348756006 | 0.582822072 | -2.314181414 | 0.020657773 |
| SELENOW | 1125.080806 | -1.118131562 | 0.457583429 | -2.44355781 | 0.014543239 |
| CYC1 | 1991.333715 | -1.819193621 | 0.721028786 | -2.523052694 | 0.011634097 |
| CALR | 536.5886167 | -1.559894284 | 0.523959933 | -2.97712513 | 0.002909652 |
| SHARPIN | 81.12159336 | -2.241974624 | 0.803830678 | -2.789113036 | 0.005285261 |
| MAF1 | 221.8572852 | -1.805484373 | 0.809726642 | -2.229745544 | 0.02576434 |
| FOXS1 | 16.49910989 | -2.688912098 | 1.229639705 | -2.186747946 | 0.028760929 |
| DCTPP1 | 315.2016416 | -1.722655435 | 0.688675534 | -2.501403562 | 0.012370213 |
| OAZ2 | 134.5209184 | -1.041658733 | 0.403924298 | -2.578846429 | 0.009913085 |
| SLC47A2 | 12.77874919 | -1.072083906 | 0.488847171 | -2.193086038 | 0.028301181 |
| H2AC25 | 32.36249624 | -1.022139902 | 0.457268657 | -2.235315907 | 0.025396616 |
| LRRC75A | 8.756362603 | -1.685070454 | 0.527488927 | -3.194513418 | 0.001400668 |
| PHLDA2 | 307.4778347 | -2.42525607 | 0.945256266 | -2.565712768 | 0.010296405 |
| ZNF716 | 56.56902311 | -1.096015787 | 0.441833969 | -2.48060553 | 0.013115943 |
| LDOC1 | 120.8990467 | -1.394123536 | 0.522173098 | -2.669849406 | 0.007588527 |
| MOB2 | 32.01656493 | -1.493393709 | 0.607044224 | -2.460106941 | 0.013889562 |
| CRIP2 | 420.0507095 | -2.203048858 | 0.855850085 | -2.574106023 | 0.010049947 |
| RUVBL2 | 339.3785022 | -2.234698608 | 0.85046913 | -2.627606963 | 0.00859878 |
| CCDC159 | 52.96353465 | -1.645514702 | 0.469834331 | -3.50232964 | 0.000461209 |
| FAM167B | 8.933747306 | -1.885886519 | 0.671634351 | -2.80790659 | 0.004986469 |
| PSG9 | 204.2359103 | -1.041032006 | 0.37192658 | -2.799025564 | 0.005125708 |
| UPP1 | 187.1450348 | -1.161781741 | 0.505844834 | -2.296715637 | 0.021634997 |
| CBX6 | 450.0702556 | -2.602111227 | 0.825640828 | -3.151626154 | 0.00162364 |
| TRAIP | 48.43963152 | -1.037684481 | 0.491933627 | -2.109399368 | 0.034910124 |
| NUDT14 | 45.70175072 | -2.057727029 | 0.677875706 | -3.035552108 | 0.002400957 |
| TSSC4 | 44.16833528 | -1.643497897 | 0.633187849 | -2.59559292 | 0.009442787 |
| THAP7 | 29.41516675 | -1.631307129 | 0.634233013 | -2.572094318 | 0.010108535 |
| TXNRD2 | 90.98416868 | -1.344886139 | 0.481758342 | -2.791619824 | 0.005244494 |
| SMIM10 | 11.78626774 | -1.900169454 | 0.566219351 | -3.355889287 | 0.000791102 |
| SIVA1 | 286.7244798 | -1.640942216 | 0.700729036 | -2.341764264 | 0.019192833 |
| HSF1 | 48.01367112 | -1.501885156 | 0.601346885 | -2.497535439 | 0.012505996 |
| IFITM2 | 148.4257364 | -1.282013811 | 0.465385895 | -2.754732844 | 0.005874008 |
| GAS2L1 | 13.3113901 | -2.086948531 | 0.785551235 | -2.65666762 | 0.007891721 |
| TMEM179B | 701.6540372 | -1.691169845 | 0.496489828 | -3.406252759 | 0.000658612 |
| PSMD13 | 1416.994275 | -1.37259669 | 0.481647022 | -2.849797934 | 0.004374701 |
| SLC52A2 | 16.82316029 | -1.536977048 | 0.647769482 | -2.372722228 | 0.017657537 |
| SPATA12 | 26.02221255 | -1.648576512 | 0.505975582 | -3.258213581 | 0.00112116 |
| TMPRSS12 | 10.55816436 | -1.232668694 | 0.599531312 | -2.056053903 | 0.039777321 |
| TMEM222 | 52.71375878 | -1.668102707 | 0.658210136 | -2.534301154 | 0.011267185 |
| SMIM29 | 40.47536136 | -1.989361738 | 0.598764736 | -3.322443052 | 0.000892329 |
| HYAL3 | 42.93878064 | -1.172246172 | 0.548918656 | -2.135555348 | 0.032715674 |
| RPS19BP1 | 531.8024945 | -1.26308418 | 0.501551617 | -2.518353321 | 0.011790498 |
| EIF4EBP1 | 1237.073459 | -3.380015121 | 0.989885758 | -3.414550714 | 0.000638873 |
| GFRAL | 5.466378289 | -2.697816479 | 0.885868322 | -3.045392202 | 0.002323771 |
| ZFTRAF1 | 47.1204999 | -1.307824543 | 0.491114121 | -2.662974829 | 0.007745321 |
| TMEM276 | 47.1204999 | -1.307824543 | 0.491114121 | -2.662974829 | 0.007745321 |
| LOC84773-CYHR1 | 47.1204999 | -1.307824543 | 0.491114121 | -2.662974829 | 0.007745321 |
| NWD1 | 33.81457962 | -1.149387438 | 0.39030589 | -2.944837541 | 0.003231243 |
| HEPACAM2 | 5.210201791 | -2.188609188 | 0.997112272 | -2.1949476 | 0.028167357 |
| PPP3R2 | 10.55609029 | -3.175571289 | 0.995824497 | -3.188886495 | 0.001428219 |
| UROS | 557.3668475 | -1.236776624 | 0.435143514 | -2.842226954 | 0.004479959 |
| UTS2B | 19.66296282 | -1.170689626 | 0.354366961 | -3.303608275 | 0.000954491 |
| CGB5 | 20.2267384 | -4.151506188 | 1.863745202 | -2.227507377 | 0.025913383 |
| TMEM120A | 25.24141045 | -1.334348984 | 0.386206278 | -3.455016292 | 0.00055026 |
| BLOC1S3 | 89.21213589 | -1.218666816 | 0.513983147 | -2.371024854 | 0.017738838 |
| CLDN4 | 8.921668946 | -1.247711915 | 0.570384227 | -2.187493722 | 0.028706501 |
| DRICH1 | 6.524134757 | -1.458782536 | 0.695699225 | -2.096858072 | 0.036006141 |
| RRP7A | 205.3929723 | -1.802549932 | 0.729730477 | -2.470158488 | 0.01350532 |
| ADAM5 | 11.76047795 | -1.493446252 | 0.456274014 | -3.273134578 | 0.001063618 |
| CGB7 | 11.66662455 | -2.610262072 | 0.89598234 | -2.913296338 | 0.00357635 |
| LONP1 | 219.7507753 | -1.140086415 | 0.535979269 | -2.127109164 | 0.033411014 |
| BORCS6 | 11.71847119 | -1.072683781 | 0.519292898 | -2.065662336 | 0.038860374 |
| PDLIM7 | 206.3106199 | -1.028782022 | 0.494283451 | -2.081360442 | 0.037400927 |
| SERTAD1 | 114.4521801 | -1.766895678 | 0.767422555 | -2.30237653 | 0.021313947 |
| HHLA3 | 64.48146013 | -2.122977679 | 0.591785298 | -3.587411998 | 0.000333976 |
| CFD | 44.49506833 | -2.217089051 | 1.069127909 | -2.073736016 | 0.038103834 |
| GPAA1 | 129.3624427 | -1.086490591 | 0.535963193 | -2.02717389 | 0.042644632 |
| DDRGK1 | 85.34883295 | -1.083472948 | 0.36089754 | -3.002162191 | 0.002680693 |
| GET3 | 138.7328455 | -3.680636988 | 1.11529072 | -3.300159252 | 0.0009663 |
| MSRB1 | 150.0072248 | -1.675904736 | 0.798035317 | -2.100038306 | 0.035725472 |
| ND5 | 6784.908057 | -1.235781122 | 0.436425336 | -2.831598028 | 0.004631603 |
| CES1 | 35.96433443 | -1.378826573 | 0.52958133 | -2.603616281 | 0.009224597 |
| R3HDM4 | 60.31316785 | -1.069146064 | 0.521432707 | -2.050400846 | 0.040325331 |
| APRT | 97.68291136 | -1.797065909 | 0.565023722 | -3.180514089 | 0.00147014 |
| SMG5 | 84.13459333 | -1.313501119 | 0.466186029 | -2.817547156 | 0.004839201 |
| RNU6-1188P | 12.96531318 | -1.161160421 | 0.543719209 | -2.135588371 | 0.03271298 |
| HSD3B1 | 9.03091127 | -2.053776134 | 0.763311703 | -2.690612662 | 0.007132095 |
| ADD3-AS1 | 14.39950153 | -1.136021826 | 0.496902312 | -2.286207568 | 0.022242118 |
| RTL8A | 456.6271799 | -1.486103276 | 0.487626212 | -3.047627956 | 0.002306553 |
| CCDC160 | 12.25592301 | -1.287515283 | 0.649747688 | -1.98156193 | 0.047528292 |
| HSD17B8 | 64.5201519 | -1.057692092 | 0.481230583 | -2.197890429 | 0.027956915 |
| OXLD1 | 39.38283125 | -1.407206543 | 0.472628867 | -2.977402869 | 0.002907017 |
| NELFE | 563.2820328 | -2.210855287 | 0.785579059 | -2.814300181 | 0.004888356 |
| PRRC2A | 72.47942369 | -1.786084047 | 0.594061649 | -3.006563457 | 0.002642189 |
| MICA | 253.945304 | -1.362489197 | 0.445798252 | -3.056291026 | 0.002240936 |
| MIR1915HG | 70.96476159 | -1.31427904 | 0.260867944 | -5.038100966 | 4.70E-07 |
| OR2H1 | 14.95950955 | -1.597112652 | 0.81367816 | -1.96283092 | 0.049665824 |
| SDHAF1 | 25.40136085 | -1.034960545 | 0.525626971 | -1.969001979 | 0.048952864 |
| E2F4 | 220.9219983 | -1.357957805 | 0.528869062 | -2.567663535 | 0.010238648 |
| SARNP | 11.60510131 | -2.047997548 | 0.6952231 | -2.945813436 | 0.003221066 |
| SLCO6A1 | 7.019221113 | -1.302950219 | 0.603051034 | -2.160596942 | 0.030726488 |
| KRT6A | 7.536075353 | -1.26686786 | 0.58278676 | -2.173810298 | 0.029719385 |
| KRT81 | 73.4711596 | -1.537948907 | 0.534514516 | -2.877281834 | 0.004011172 |
| SLC48A1 | 96.53285664 | -1.552171903 | 0.523896138 | -2.962747368 | 0.003049067 |
| IGHG3 | 5.110773031 | -2.051046056 | 0.767683965 | -2.671732312 | 0.007546082 |
| ZNF580 | 39.34273217 | -2.112003479 | 0.668055297 | -3.161420153 | 0.001570019 |
| CGB8 | 10.02013966 | -4.314852836 | 1.081333306 | -3.990307902 | 6.60E-05 |
| TRIM59 | 2255.33066 | -1.039034356 | 0.345344353 | -3.00869074 | 0.00262376 |
| QTRT1 | 16.18493193 | -1.467583343 | 0.574627115 | -2.553975097 | 0.010650087 |
| ARL2 | 280.0879988 | -2.728744019 | 0.860962543 | -3.169410845 | 0.001527483 |
| SRA1 | 1555.831215 | -1.105009697 | 0.414220581 | -2.667684193 | 0.007637601 |
| RPLP0P6 | 133.8678608 | -1.374836152 | 0.505057749 | -2.722136535 | 0.006486134 |
| SLC35F6 | 178.3709979 | -1.211195203 | 0.476070823 | -2.5441492 | 0.010954428 |
| TSPAN4 | 48.57047335 | -1.073359232 | 0.485534874 | -2.210673817 | 0.027058433 |
| ALG3 | 327.0706049 | -1.749877417 | 0.708439946 | -2.470043405 | 0.013509666 |
| IFRD2 | 35.42216867 | -2.597083125 | 0.958633229 | -2.709151996 | 0.006745543 |
| NLRP2B | 16.885996 | -3.469376307 | 1.529741704 | -2.267949091 | 0.02333231 |
| LOC100129844 | 7.293276105 | -3.775892908 | 1.586781969 | -2.379591515 | 0.017331839 |
| VAMP2 | 23.97780266 | -1.393549704 | 0.527469011 | -2.641955595 | 0.008242886 |
| RNU6-101P | 5.896932651 | -1.696826823 | 0.727055842 | -2.33383287 | 0.019604475 |
| RNY1P15 | 22.88239999 | -1.360693634 | 0.374063367 | -3.637601947 | 0.000275188 |
| LINC00370 | 10.71745108 | -1.338038723 | 0.63231 | -2.116111911 | 0.034335291 |
| CPTP | 15.8800012 | -1.524935361 | 0.725094963 | -2.103083648 | 0.035458458 |
| LINC00691 | 5.792283472 | -2.110049065 | 0.955313088 | -2.208751342 | 0.027191941 |
| LOC105373175 | 9.036783189 | -1.961941986 | 0.995550126 | -1.970711402 | 0.048756896 |
| LINC02470 | 12.6653699 | -2.783788605 | 0.771082886 | -3.610232643 | 0.000305922 |
| LINC01623 | 7.783205195 | -1.596672378 | 0.789220557 | -2.023100341 | 0.043062803 |
| MCRIP1 | 183.6390461 | -2.425210595 | 0.827726774 | -2.929965144 | 0.00339 |
| PTPRD-AS1 | 8.368425732 | -1.523745182 | 0.586800343 | -2.596701244 | 0.009412375 |
| TMEM191A | 27.14119506 | -1.128287119 | 0.410506961 | -2.748521284 | 0.005986475 |
| TAS2R46 | 6.052915025 | -1.508985206 | 0.703961748 | -2.143561365 | 0.032068049 |
| LYPLAL1-DT | 16.18392653 | -1.438203751 | 0.577609449 | -2.489924212 | 0.012777034 |
| FAM174C | 157.7605331 | -3.406888857 | 1.009293949 | -3.375516975 | 0.000736771 |
| RLIMP1 | 6.965690667 | -1.245185252 | 0.615838042 | -2.021936236 | 0.04318294 |
| SERBP1P4 | 5.267190992 | -4.300646919 | 2.193982936 | -1.960200715 | 0.049972335 |
| LINC00309 | 11.05504074 | -2.062318666 | 0.729998956 | -2.825098105 | 0.004726614 |
| MRPS9-AS1 | 6.128929484 | -1.468401602 | 0.677158498 | -2.168475485 | 0.030122528 |
| RPL7L1P12 | 6.746471607 | -1.95733602 | 0.671268166 | -2.915877917 | 0.003546893 |
| LINC00867 | 17.24275056 | -1.33337915 | 0.460306591 | -2.896719653 | 0.003770866 |
| LINC02518 | 5.145848317 | -2.000350704 | 0.844639905 | -2.368288181 | 0.01787061 |
| RGS5-AS1 | 8.245406822 | -1.506167947 | 0.626629703 | -2.403601264 | 0.01623447 |
| LOC124904411 | 7.373574805 | -1.710814383 | 0.5611623 | -3.048698002 | 0.002298354 |
| LOC101929297 | 5.208454105 | -1.479979342 | 0.729194196 | -2.029609327 | 0.042396266 |
| LINC01503 | 17.50800508 | -1.171762632 | 0.503065965 | -2.329242512 | 0.019846222 |
| RNASEH1-DT | 98.46461869 | -1.052194577 | 0.412396644 | -2.551414011 | 0.01072868 |
| LOC100419824 | 5.737120142 | -1.799066843 | 0.735236919 | -2.446921255 | 0.014408231 |
| FTH1P2 | 241.4084858 | -1.996215693 | 0.784632613 | -2.544140606 | 0.010954698 |
| LINC00330 | 6.407609206 | -1.552385406 | 0.770922493 | -2.013672476 | 0.044043932 |
| LOC101927020 | 7.94113888 | -1.82647661 | 0.68086212 | -2.682593958 | 0.007305363 |
| KRT18P63 | 7.486188618 | -2.042382585 | 0.761162514 | -2.683241157 | 0.007291239 |
| NFAM1 | 11.0700307 | -1.247484731 | 0.604772718 | -2.062733147 | 0.039137989 |
| LINC01102 | 8.44392492 | -1.114908439 | 0.537583089 | -2.073927661 | 0.03808603 |
| RPL13AP12 | 16.22002008 | -2.080098912 | 0.783341834 | -2.655416604 | 0.007921052 |
| LINC01010 | 22.77375454 | -1.261968373 | 0.480814703 | -2.624645971 | 0.00867391 |
| NDUFA6-DT | 11.89781848 | -1.008366281 | 0.509390899 | -1.979552998 | 0.047753779 |
| LINC00851 | 5.667330347 | -2.61509675 | 0.956983049 | -2.732646888 | 0.006282765 |
| OR7E24 | 5.708488743 | -2.645173742 | 1.015994502 | -2.603531551 | 0.009226877 |
| IRGM | 11.15932758 | -1.997218466 | 0.789666365 | -2.529192775 | 0.011432521 |
| MTATP6P20 | 5.91039365 | -3.41771781 | 1.151430291 | -2.968236841 | 0.002995134 |
| OR2W3 | 5.964098369 | -1.655134587 | 0.619532325 | -2.671587132 | 0.007549347 |
| LOC124901856 | 9.996758051 | -1.734909833 | 0.856629099 | -2.02527539 | 0.042839094 |
| RPL34P28 | 12.70237411 | -1.137707999 | 0.525921908 | -2.163264131 | 0.03052087 |
| RPS2P5 | 162.2056075 | -1.531801409 | 0.534089935 | -2.868058931 | 0.004129986 |
| ARPC4 | 673.7109213 | -1.570880809 | 0.468626575 | -3.352095024 | 0.000802025 |
| RPL14P3 | 12.88354627 | -1.067307557 | 0.539294222 | -1.979082129 | 0.04780676 |
| EIF6 | 332.2697449 | -1.221257606 | 0.528701846 | -2.309917422 | 0.020892726 |
| RPL23AP55 | 8.758137116 | -2.086427786 | 0.803727247 | -2.595940095 | 0.009433251 |
| C4orf48 | 92.95021192 | -2.071198337 | 0.864426423 | -2.396037745 | 0.016573384 |
| AOX2P | 12.00130918 | -1.090081994 | 0.527128376 | -2.067963032 | 0.038643499 |
| RPS6P17 | 9.967970646 | -2.302424412 | 0.744845919 | -3.091141876 | 0.001993883 |
| DDOST | 368.7087156 | -1.40717285 | 0.516887901 | -2.72239464 | 0.00648107 |
| LINC01550 | 7.191334266 | -1.572436951 | 0.546806989 | -2.875670909 | 0.004031698 |
| TWF2 | 67.96547225 | -1.29286026 | 0.58065583 | -2.226551759 | 0.025977246 |
| NOL8P1 | 8.084111778 | -2.479553861 | 0.98200208 | -2.524998583 | 0.011569876 |
| LNCBRM | 14.73795449 | -1.640932477 | 0.715194381 | -2.294386704 | 0.021768296 |
| ZNF286B | 21.05278579 | -1.047787173 | 0.430062657 | -2.436359342 | 0.014835938 |
| PCGF3-AS1 | 32.8530059 | -1.042237067 | 0.447834169 | -2.327283487 | 0.019950182 |
| LINC02372 | 13.92948635 | -2.336869806 | 0.730166914 | -3.200459734 | 0.001372085 |
| EEF1A1P20 | 6.175103585 | -3.235111515 | 1.006845669 | -3.21311559 | 0.001313034 |
| LOC100289037 | 6.71363119 | -4.350234514 | 1.924858883 | -2.260027762 | 0.023819528 |
| LINC02701 | 9.210029804 | -1.976819603 | 0.715275273 | -2.763718637 | 0.005714679 |
| GLI4 | 11.28755696 | -1.289839942 | 0.618143524 | -2.086635048 | 0.036921137 |
| LINC02492 | 10.16658594 | -1.525745989 | 0.669703776 | -2.278240088 | 0.022712277 |
| LOC391713 | 6.206812854 | -6.116482898 | 2.312069254 | -2.645458343 | 0.008158033 |
| YTHDF1P1 | 8.308368267 | -3.24259168 | 1.612963433 | -2.010331799 | 0.044396083 |
| LINC02160 | 7.935690312 | -1.879230825 | 0.684713146 | -2.744551989 | 0.006059356 |
| HNRNPKP3 | 7.350207942 | -1.811671632 | 0.792861584 | -2.284978448 | 0.022314092 |
| RNU6-897P | 10.52109007 | -1.40381468 | 0.541663857 | -2.591671312 | 0.009551098 |
| PCDHGA12 | 8.959699674 | -2.850478186 | 1.096911838 | -2.598639279 | 0.009359407 |
| RAD21-AS1 | 11.8171063 | -1.308342214 | 0.600782788 | -2.177729189 | 0.029426204 |
| MINCR | 50.53447284 | -1.172419218 | 0.301978658 | -3.882457204 | 0.000103406 |
| UQCRB-AS1 | 9.902276118 | -1.430678329 | 0.649908486 | -2.201353514 | 0.027711007 |
| PBOV1 | 18.98808076 | -2.287412164 | 0.81130839 | -2.81941145 | 0.00481118 |
| OR4D10 | 24.38959429 | -2.881196759 | 1.171801939 | -2.458774527 | 0.013941214 |
| FLJ20021 | 17.99381007 | -1.431446135 | 0.568275206 | -2.518931181 | 0.011771166 |
| LOC100420800 | 7.086221587 | -2.749924949 | 1.081452558 | -2.542806829 | 0.0109966 |
| MPV17L2 | 58.22373575 | -1.041122109 | 0.500193095 | -2.081440384 | 0.037393615 |
| SMIM35 | 7.424268796 | -1.218211045 | 0.571938485 | -2.129968654 | 0.033174201 |
| A2MP1 | 9.127252504 | -1.240815806 | 0.539611517 | -2.299461312 | 0.021478758 |
| URB1-AS1 | 174.7416321 | -1.114848077 | 0.333177177 | -3.346111781 | 0.000819534 |
| LNCOG | 87.46327521 | -1.001302928 | 0.389984176 | -2.56754758 | 0.010242073 |
| LINC02401 | 11.28700347 | -1.351549604 | 0.577183033 | -2.341630863 | 0.019199694 |
| MAP1LC3B2 | 9.293408227 | -1.250908033 | 0.563486777 | -2.219942125 | 0.026422696 |
| LOC105370854 | 17.89141876 | -1.089870769 | 0.431186286 | -2.527610005 | 0.011484184 |
| LOC102723670 | 12.88602341 | -1.454185802 | 0.526383617 | -2.762597002 | 0.005734352 |
| LOC101928417 | 13.589316 | -2.342697895 | 0.662220154 | -3.537642098 | 0.000403717 |
| LINC02516 | 11.27413362 | -1.279742938 | 0.585421852 | -2.186018395 | 0.02881426 |
| LINC02180 | 9.446804669 | -1.37094841 | 0.628202292 | -2.182335893 | 0.029084752 |
| MMP12 | 46.55729939 | -1.737751178 | 0.65859577 | -2.638570208 | 0.008325646 |
| MRPL12 | 206.720481 | -1.57528815 | 0.710071805 | -2.218491339 | 0.026521349 |
| DPEP2NB | 7.440269427 | -2.361141307 | 1.058386836 | -2.23088688 | 0.025688623 |
| LINC01919 | 7.864053443 | -1.536147305 | 0.666086371 | -2.306228398 | 0.021097874 |
| SCAT1 | 16.14433374 | -1.069422489 | 0.413477394 | -2.586411022 | 0.009698121 |
| DPY19L3-DT | 10.42842122 | -1.839964014 | 0.705562561 | -2.607797118 | 0.009112695 |
| S1PR2 | 17.534617 | -1.1419167 | 0.419603178 | -2.721420523 | 0.006500201 |
| LINC01028 | 6.167670779 | -3.302955675 | 1.033999771 | -3.194348557 | 0.001401468 |
| LINC01711 | 61.27595925 | -1.06066221 | 0.427062162 | -2.483624876 | 0.013005273 |
| CCNYL6 | 17.6235364 | -1.0717281 | 0.510116264 | -2.100948698 | 0.03564547 |
| NCBP2AS2 | 512.8245778 | -1.152964258 | 0.450150791 | -2.561284531 | 0.010428591 |
| HNRNPA3P9 | 15.16989028 | -3.460633444 | 0.671116421 | -5.156532213 | 2.52E-07 |
| TMEM14B-DT | 11.22814467 | -1.496984257 | 0.59639717 | -2.510045876 | 0.012071548 |
| H2BC8 | 7.016371965 | -1.89449867 | 0.633727562 | -2.989452856 | 0.002794776 |
| H3C8 | 17.89943436 | -2.604395771 | 0.480237261 | -5.423143899 | 5.86E-08 |
| ZNF2 | 111.4807107 | -1.239104794 | 0.313162895 | -3.956742039 | 7.60E-05 |
| CCL16 | 5.773029944 | -2.418856423 | 1.007992271 | -2.39967755 | 0.01640952 |
| LOC158434 | 10.72282982 | -1.963265892 | 0.68672244 | -2.858892876 | 0.004251223 |
| H3C1 | 9.749029246 | -1.414517794 | 0.55367864 | -2.554763163 | 0.010626007 |
| RAB7B | 9.403978234 | -1.106808533 | 0.481007578 | -2.301020992 | 0.021390445 |
| H4C5 | 54.15808755 | -1.009598019 | 0.369193649 | -2.73460289 | 0.006245557 |
| H2AC8 | 19.82756607 | -1.645281868 | 0.489166095 | -3.363442159 | 0.00076977 |
| CISD3 | 23.43708055 | -1.313476231 | 0.522376733 | -2.514423303 | 0.011922723 |
| H3C10 | 23.20937421 | -1.433945852 | 0.349510773 | -4.102722899 | 4.08E-05 |
| CEP83-DT | 7.247622281 | -1.453840718 | 0.622004501 | -2.337347586 | 0.019421118 |
| EEF1AKMT4 | 38.5756343 | -1.176194813 | 0.533060559 | -2.206493789 | 0.027349445 |
| H3C2 | 26.03019077 | -1.03486524 | 0.431807181 | -2.396591084 | 0.01654838 |
| H3C3 | 25.68424608 | -1.228955267 | 0.380689434 | -3.228235818 | 0.001245562 |
| LOC107985177 | 9.417289147 | -1.363167571 | 0.686263512 | -1.98636172 | 0.046993174 |
| LOC124903825 | 15.78256192 | -1.493494071 | 0.641647751 | -2.327591842 | 0.019933787 |
| LOC101928357 | 6.581203769 | -3.675504031 | 1.245551591 | -2.950904689 | 0.003168447 |
| LOC107986626 | 13.22640515 | -1.165510988 | 0.466853033 | -2.496526545 | 0.012541628 |
