# Supplementary material for: Extrachromosomal circular DNA expressing miRNA promotes ovarian cancer progression
Source: Clin Transl Med. 2025 Sep 23;15(9):e70445. doi: 10.1002/ctm2.70445 (PMC12455017; doi:10.1002/ctm2.70445)
Supplement: Supplementary file 8 — Supporting Information [file CTM2-15-e70445-s010.docx]

**Table S3 Differentially expressed genes after overexpression of eccMIR3661 (pvalue < 0.05)**

| SYMBOL | baseMean | log2FoldChange | lfcSE | stat | pvalue |
| --- | --- | --- | --- | --- | --- |
| TFPI | 1135.152527 | 0.304189088 | 0.155058012 | 1.961776007 | 0.049788568 |
| RBM5 | 117.1900047 | -0.850690366 | 0.302848795 | -2.808960707 | 0.004970171 |
| ARF5 | 23.12067853 | -3.111133921 | 0.828729653 | -3.754099915 | 0.000173965 |
| ITGA3 | 126.7514499 | -1.473217122 | 0.488606711 | -3.015138942 | 0.002568616 |
| TNFRSF12A | 2053.619525 | -3.456053538 | 1.026101258 | -3.368140826 | 0.000756769 |
| RALA | 982.4320695 | 0.243768478 | 0.115292446 | 2.114349082 | 0.034485463 |
| AGK | 88.27715774 | -0.439586816 | 0.220322732 | -1.99519501 | 0.046021615 |
| PROM1 | 10.84728847 | -1.946133522 | 0.546578198 | -3.560576562 | 0.000370041 |
| CCDC124 | 235.6123853 | -3.246277264 | 1.005387352 | -3.228882141 | 0.001242751 |
| TRAPPC6A | 21.13866508 | -1.701891338 | 0.694026338 | -2.45219993 | 0.014198575 |
| MATK | 3.760442555 | -1.825640386 | 0.859881193 | -2.123130964 | 0.033742879 |
| ST7L | 105.8010633 | -0.499566026 | 0.194643967 | -2.566563112 | 0.010271193 |
| PAX6 | 12.09632654 | 1.019556839 | 0.4652977 | 2.191192516 | 0.028437864 |
| SELE | 4.136452973 | -2.578754611 | 1.032409989 | -2.497800912 | 0.012496635 |
| DLEC1 | 7.271422481 | 1.281824603 | 0.637127469 | 2.011880927 | 0.04423249 |
| CYTH3 | 11.94931776 | -1.123728668 | 0.507964576 | -2.212218571 | 0.026951566 |
| MED24 | 65.65863453 | -1.034630887 | 0.425076297 | -2.433988663 | 0.014933463 |
| UQCRC1 | 79.10601987 | -2.376782301 | 0.872702972 | -2.723472221 | 0.006459965 |
| FMO1 | 6.155081049 | -1.872843191 | 0.784968981 | -2.385881782 | 0.017038227 |
| ELOA | 356.3646401 | 0.338602377 | 0.146721009 | 2.307797494 | 0.021010402 |
| RABGAP1 | 371.0638519 | 0.428375551 | 0.158411908 | 2.704187808 | 0.006847158 |
| HEBP1 | 392.9760323 | -0.411572372 | 0.155138728 | -2.652931201 | 0.007979613 |
| BID | 279.343976 | -0.938956956 | 0.404879704 | -2.319101075 | 0.020389556 |
| RALBP1 | 383.5512818 | 0.3561006 | 0.122353185 | 2.910431804 | 0.003609297 |
| GRAMD1B | 26.79872177 | 0.830496763 | 0.396491837 | 2.094612514 | 0.036205452 |
| RNH1 | 110.9696459 | -1.504646576 | 0.745038161 | -2.019556387 | 0.043429423 |
| ABCC2 | 13.42720718 | 1.156286952 | 0.550605754 | 2.100027003 | 0.035726466 |
| UBR2 | 577.5172852 | -0.322681174 | 0.141925004 | -2.273603421 | 0.022989842 |
| EHD2 | 115.8818432 | -1.605624548 | 0.768340999 | -2.089729105 | 0.036642141 |
| HSD17B6 | 14.97161674 | -1.504835262 | 0.553066092 | -2.720895899 | 0.006510526 |
| BTN3A1 | 11.85074163 | -1.103454342 | 0.523009981 | -2.109815073 | 0.034874288 |
| GRN | 61.99686139 | -1.915703331 | 0.642034702 | -2.983800291 | 0.002846925 |
| RANBP3 | 75.56022657 | -1.66146209 | 0.627772704 | -2.646598169 | 0.00813059 |
| ALG1 | 23.93637724 | -0.798806669 | 0.384279506 | -2.078712647 | 0.03764377 |
| MAP2K3 | 16.55910238 | -1.296947225 | 0.577986878 | -2.243904273 | 0.024838561 |
| TIMP2 | 3110.955046 | 0.337280352 | 0.161388712 | 2.089863333 | 0.036630078 |
| RFC1 | 1341.247466 | 0.386230775 | 0.113400418 | 3.405902554 | 0.000659457 |
| CYP46A1 | 33.78771425 | -1.994658435 | 0.573176387 | -3.480008042 | 0.000501399 |
| TUBG2 | 49.67829759 | -1.58726787 | 0.693534841 | -2.28866349 | 0.022098912 |
| METTL1 | 87.4852267 | -0.9877546 | 0.455113504 | -2.170347814 | 0.029980507 |
| THAP3 | 83.08050412 | -1.544837212 | 0.577000777 | -2.677357245 | 0.007420547 |
| CAPG | 180.8052014 | -2.137841769 | 0.814934361 | -2.623330015 | 0.008707488 |
| AP2S1 | 808.7783748 | -2.308370369 | 0.780544048 | -2.957386422 | 0.00310259 |
| USH2A | 19.72375029 | -0.921569891 | 0.470139239 | -1.960206283 | 0.049971684 |
| ADAM28 | 16.86807001 | 1.120760324 | 0.529615215 | 2.116178484 | 0.03432963 |
| HSPA5 | 41088.53105 | 0.578684585 | 0.235151534 | 2.460900739 | 0.01385887 |
| ARAP2 | 182.7543543 | 0.460434803 | 0.219298845 | 2.099576965 | 0.035766071 |
| LMO3 | 28.07533995 | -0.92203582 | 0.418724523 | -2.202010557 | 0.027664563 |
| RFC2 | 130.2690584 | -0.78256791 | 0.331256445 | -2.362423198 | 0.018155903 |
| PTGER3 | 13.74030357 | -2.001207337 | 0.721255788 | -2.774615289 | 0.005526704 |
| CYBA | 20.76196092 | -1.534587085 | 0.659390852 | -2.327279913 | 0.019950372 |
| THOC3 | 144.408741 | -0.483657585 | 0.184645328 | -2.619387066 | 0.008808793 |
| MCF2L2 | 32.43724394 | -1.02191987 | 0.480454836 | -2.126984253 | 0.033421392 |
| SZRD1 | 133.3003512 | -2.347446727 | 0.801008476 | -2.930614091 | 0.003382927 |
| TRAF1 | 9.921946401 | 1.445006101 | 0.573454638 | 2.519826336 | 0.011741275 |
| RPL18 | 221.0467611 | -0.892167436 | 0.43603907 | -2.046072237 | 0.040749264 |
| ISOC2 | 85.56883064 | -2.886389502 | 0.942884177 | -3.061234427 | 0.002204264 |
| RNF4 | 508.9140818 | 0.321170163 | 0.136352352 | 2.355442789 | 0.018500646 |
| HMG20B | 19.41767463 | -4.435005519 | 1.777310884 | -2.495345951 | 0.012583438 |
| ANKS1A | 35.98631305 | 0.760801143 | 0.3555078 | 2.140040648 | 0.032351482 |
| NTHL1 | 28.14962556 | -1.642839024 | 0.656722638 | -2.501572092 | 0.012364327 |
| WDR18 | 49.70689679 | -1.99625417 | 0.957678756 | -2.084471602 | 0.037117291 |
| ELOVL1 | 21.00225628 | -1.476030546 | 0.647451736 | -2.279753787 | 0.022622295 |
| MPPED2 | 18.25402033 | -0.905412238 | 0.392605502 | -2.306162888 | 0.021101532 |
| GOLGA5 | 422.1985658 | 0.312229588 | 0.143146254 | 2.181192859 | 0.029169155 |
| PKM | 891.3388071 | -0.830387726 | 0.414754525 | -2.002118546 | 0.045271983 |
| HYAL2 | 29.61710161 | -1.422039718 | 0.582232426 | -2.442391827 | 0.014590301 |
| IFI35 | 13.55934388 | -1.854204107 | 0.792465874 | -2.339790479 | 0.019294561 |
| TFE3 | 6.901127975 | -1.276690393 | 0.612698554 | -2.083717001 | 0.037185917 |
| FTSJ1 | 49.15529488 | -1.650736263 | 0.586259798 | -2.815707762 | 0.004866992 |
| IFT80 | 44.42872432 | -0.783112391 | 0.302185715 | -2.591493749 | 0.009556028 |
| SIRT2 | 27.2806177 | -1.825437436 | 0.75182353 | -2.428013177 | 0.015181792 |
| TRPC7 | 5.994005156 | -1.529776878 | 0.68990538 | -2.217372009 | 0.026597681 |
| TMEM260 | 112.9229334 | 0.550993191 | 0.20581967 | 2.677067697 | 0.007426963 |
| FSTL3 | 65.94679488 | -1.329757756 | 0.579804942 | -2.293457093 | 0.021821703 |
| RNF126 | 51.95299525 | -2.746245031 | 1.022301191 | -2.686336526 | 0.007224029 |
| CDC42 | 57.15091668 | -0.680850114 | 0.27375631 | -2.487066377 | 0.012880136 |
| ASIC4 | 5.268044735 | 1.396345535 | 0.649691085 | 2.149245337 | 0.031614957 |
| HSD17B10 | 116.9472219 | -1.353604703 | 0.45301196 | -2.988010966 | 0.002807995 |
| KDM5A | 327.3336638 | 0.399279648 | 0.193158204 | 2.067112036 | 0.038723598 |
| PTGS2 | 82.61083287 | 1.414562182 | 0.405333507 | 3.489872313 | 0.000483251 |
| TEAD2 | 5.567812513 | -1.63038199 | 0.752386259 | -2.16694812 | 0.03023881 |
| ENO1 | 935.7221866 | -0.899110002 | 0.449935414 | -1.998309034 | 0.045683167 |
| MYDGF | 1040.509468 | -1.377147602 | 0.520868378 | -2.643945497 | 0.008194585 |
| ZNF37A | 507.5843488 | 0.273075509 | 0.136661026 | 1.998195954 | 0.04569542 |
| RAB7A | 2432.950734 | -0.303557607 | 0.149110963 | -2.035783295 | 0.041772121 |
| EXOSC7 | 227.8894904 | -0.830991024 | 0.355725608 | -2.336044985 | 0.019488897 |
| MKRN2 | 35.57929918 | -1.044090583 | 0.508201251 | -2.054482512 | 0.039929014 |
| STXBP2 | 3.252657069 | -1.91627577 | 0.917705435 | -2.0881164 | 0.036787336 |
| SNRPA | 20.45713871 | -3.624010859 | 1.356192126 | -2.672195767 | 0.007535667 |
| EXOSC5 | 55.76419023 | -1.247636288 | 0.5605855 | -2.225595005 | 0.026041321 |
| CAPZB | 409.2361587 | -0.967796439 | 0.418971093 | -2.30993607 | 0.020891694 |
| ARAF | 29.19896367 | -1.139366129 | 0.531644527 | -2.143097635 | 0.032105259 |
| CDH17 | 4.964116971 | -2.052383995 | 0.902460487 | -2.274209259 | 0.022953408 |
| CDH7 | 10.46609599 | 1.292483639 | 0.594712187 | 2.173292675 | 0.029758296 |
| FYB1 | 28.47040988 | -1.163273755 | 0.442588298 | -2.628342771 | 0.008580201 |
| STRADB | 132.9922202 | -0.528186573 | 0.258780187 | -2.041062647 | 0.041244601 |
| ZNF324 | 8.956235456 | -1.467187959 | 0.618986219 | -2.370307957 | 0.017773275 |
| GSTP1 | 1328.894291 | -0.96718031 | 0.416952918 | -2.319639144 | 0.020360406 |
| CTTN | 1287.39662 | -1.297186429 | 0.484238121 | -2.678819311 | 0.007388225 |
| MTIF2 | 618.5133838 | 0.269337219 | 0.128304326 | 2.099206063 | 0.03579874 |
| SRRT | 24.46064574 | -2.159896453 | 0.653100759 | -3.30714124 | 0.000942534 |
| TMPRSS11E | 3.531717246 | 2.02507719 | 0.896040317 | 2.2600291 | 0.023819445 |
| PGS1 | 23.09471015 | -1.118228602 | 0.439558453 | -2.543981567 | 0.010959687 |
| MT3 | 3.371809601 | 2.250013154 | 1.039679159 | 2.164141826 | 0.030453466 |
| NID2 | 20.83531881 | 1.246051078 | 0.586148032 | 2.125830012 | 0.033517416 |
| PDRG1 | 176.3254485 | -1.039970468 | 0.465596674 | -2.233629502 | 0.025507461 |
| EPB41L1 | 63.18871246 | -0.806786195 | 0.335026515 | -2.408126399 | 0.016034628 |
| CRLS1 | 57.22077466 | -0.609881522 | 0.304410387 | -2.003484596 | 0.045125299 |
| KIZ | 112.7657407 | 0.522854745 | 0.214082668 | 2.442303014 | 0.014593892 |
| CFAP61 | 13.95595957 | -1.123992596 | 0.442627101 | -2.539366869 | 0.01110533 |
| PEBP1 | 557.9506487 | -0.78114502 | 0.369518814 | -2.113951954 | 0.034519371 |
| FXYD5 | 1652.125495 | -2.182981445 | 0.849998236 | -2.568218795 | 0.010222261 |
| GRAMD1A | 37.54976921 | -1.346602584 | 0.587715713 | -2.291248225 | 0.021949061 |
| MLF2 | 243.1686187 | -1.108568579 | 0.479301579 | -2.312883218 | 0.020729061 |
| STRN4 | 62.04324337 | -1.157303049 | 0.546128464 | -2.119104068 | 0.034081673 |
| PITPNM2 | 13.55707091 | 0.916714599 | 0.42017416 | 2.181749108 | 0.029128054 |
| POU4F3 | 6.3944427 | 1.741089392 | 0.617581119 | 2.819207611 | 0.004814237 |
| NLRC4 | 6.223093458 | -1.480133513 | 0.612710353 | -2.415714875 | 0.015704353 |
| COMT | 43.7064144 | -2.28247573 | 0.658132165 | -3.468111498 | 0.00052413 |
| OR1I1 | 4.682966621 | -2.821617952 | 0.959007067 | -2.942228529 | 0.003258594 |
| AAAS | 13.40401275 | -1.456638013 | 0.672710725 | -2.165325985 | 0.03036273 |
| SEMA4G | 5.101578991 | 1.783745037 | 0.675473681 | 2.640732108 | 0.00827271 |
| BAMBI | 109.9254494 | 0.685660807 | 0.275743661 | 2.486587745 | 0.012897476 |
| NUBP2 | 83.64055764 | -2.251057184 | 0.825346478 | -2.727408722 | 0.006383392 |
| KCNK16 | 4.480793367 | 1.733162248 | 0.680563244 | 2.546658613 | 0.010875977 |
| MRPS18A | 39.21026317 | -1.244182101 | 0.629802689 | -1.975510937 | 0.048210195 |
| EFHC1 | 319.8932741 | -0.61230988 | 0.169311061 | -3.616478913 | 0.000298638 |
| ACOT7 | 109.0998947 | -1.901420649 | 0.86562944 | -2.196575765 | 0.028050759 |
| PCSK5 | 124.7607439 | -0.663716108 | 0.224410894 | -2.95759308 | 0.003100511 |
| SCD | 528.6583675 | 0.29930201 | 0.143263556 | 2.089170612 | 0.036692368 |
| TSPAN15 | 8.007105545 | 1.184785422 | 0.527831697 | 2.24462727 | 0.024792071 |
| ATP5F1D | 102.7847671 | -2.725462833 | 0.864405631 | -3.152990606 | 0.00161607 |
| NDUFB7 | 474.4764473 | -1.249162496 | 0.516700082 | -2.417577506 | 0.015624206 |
| TECR | 161.5846281 | -2.083326635 | 0.859891751 | -2.42277779 | 0.015402345 |
| TIMM13 | 108.3744839 | -1.501454702 | 0.751603927 | -1.997667454 | 0.045752725 |
| POLR2E | 119.8990836 | -1.884328013 | 0.820269341 | -2.297206439 | 0.021606996 |
| RASSF7 | 15.89163425 | -2.918196096 | 1.234038118 | -2.364753611 | 0.01804207 |
| CABIN1 | 18.80783929 | -1.034836616 | 0.45049053 | -2.297132894 | 0.02161119 |
| RNF215 | 11.14037744 | -1.249826692 | 0.628990779 | -1.987034998 | 0.046918519 |
| SEC14L2 | 50.2741624 | -1.160964961 | 0.441565879 | -2.629199891 | 0.008558604 |
| SRRD | 44.08042759 | -0.872984561 | 0.316224827 | -2.760645233 | 0.00576873 |
| CENPM | 312.7725916 | -1.823862631 | 0.68735725 | -2.65344205 | 0.007967545 |
| LMF2 | 33.03042462 | -1.739103786 | 0.764960686 | -2.273455116 | 0.022998768 |
| TTLL1 | 5.256010416 | -2.53017176 | 0.805908273 | -3.139528213 | 0.001692201 |
| TSPO | 71.53013404 | -1.247948709 | 0.564290277 | -2.21153679 | 0.026998687 |
| TXN2 | 296.8541825 | -1.424357813 | 0.501811474 | -2.838432136 | 0.004533576 |
| FOXRED2 | 53.89906507 | -0.571144513 | 0.259172058 | -2.203727199 | 0.027543535 |
| IL2RB | 8.531405881 | 1.585385713 | 0.737048805 | 2.150991499 | 0.031476871 |
| TRMU | 155.484018 | -1.155822136 | 0.445247045 | -2.595911976 | 0.009434023 |
| PMM1 | 55.79444655 | -2.084255958 | 0.670122422 | -3.110261482 | 0.001869218 |
| GZMB | 2.792205923 | 2.519235263 | 1.27288126 | 1.979159677 | 0.047798031 |
| PSMB5 | 866.8417793 | -0.845470036 | 0.308927684 | -2.73678948 | 0.006204198 |
| ARHGAP5 | 784.6759027 | 0.392980065 | 0.12335442 | 3.185780168 | 0.001443642 |
| PRORP | 26.63855819 | -1.513577602 | 0.563680532 | -2.685169197 | 0.00724931 |
| PSMA6 | 105.8962561 | -0.551964763 | 0.273836588 | -2.015672074 | 0.043834278 |
| PROCR | 48.75351096 | -1.025714754 | 0.440419562 | -2.328949124 | 0.019861761 |
| MYBL2 | 229.5107034 | -0.924287946 | 0.446819713 | -2.06859259 | 0.038584334 |
| RIMS4 | 4.141057554 | 2.219432753 | 0.841440603 | 2.637658255 | 0.008348066 |
| PSMA7 | 4774.993761 | -0.549098018 | 0.252307757 | -2.176302561 | 0.029532644 |
| MRGBP | 88.93140882 | -0.860598836 | 0.430361351 | -1.999712181 | 0.045531352 |
| C20orf27 | 53.94302969 | -3.933885982 | 1.104816787 | -3.560668182 | 0.000369912 |
| RNF24 | 130.3391323 | -0.952742979 | 0.335705252 | -2.838034179 | 0.004539232 |
| ARFRP1 | 101.1983097 | -1.280532585 | 0.590042561 | -2.170237657 | 0.029988847 |
| SEL1L2 | 7.296957845 | -1.256731344 | 0.577714187 | -2.175351361 | 0.029603796 |
| PDYN | 3.574575319 | 2.155942986 | 0.832380816 | 2.590092112 | 0.009595025 |
| ADNP2 | 815.9335566 | 0.379222796 | 0.154650297 | 2.452131055 | 0.014201293 |
| RBFA | 8.232501032 | -1.599039515 | 0.610011859 | -2.621325293 | 0.008758864 |
| CSTF2 | 223.4520359 | -0.521506935 | 0.239153147 | -2.18064007 | 0.029210049 |
| VSIG1 | 3.793067973 | 2.299619872 | 0.972547692 | 2.364531725 | 0.018052881 |
| ATG4A | 127.5755413 | 0.649333463 | 0.323964531 | 2.004335042 | 0.045034183 |
| GPR143 | 6.145479935 | -1.849449478 | 0.815842886 | -2.266918678 | 0.023395194 |
| MID1 | 319.3204209 | 0.364990426 | 0.168708109 | 2.163443291 | 0.030507101 |
| ABCD1 | 7.752655544 | -1.731664253 | 0.626717458 | -2.763070072 | 0.005726047 |
| PLP2 | 101.0191979 | -1.263822543 | 0.564516825 | -2.238768602 | 0.025170976 |
| PCSK1N | 5.43440757 | -2.822007127 | 1.111829162 | -2.538166135 | 0.011143507 |
| HTATSF1 | 1832.777015 | 0.385215588 | 0.153886236 | 2.503249136 | 0.012305889 |
| TIMP1 | 2753.386354 | -1.724087198 | 0.629811313 | -2.737466226 | 0.006191448 |
| GABRE | 101.698456 | 0.646950587 | 0.272054089 | 2.378021918 | 0.017405791 |
| SRPX2 | 5.069304463 | 1.621602546 | 0.717222623 | 2.260947289 | 0.023762521 |
| RUBCNL | 15.724134 | 0.861917651 | 0.390231033 | 2.208736819 | 0.027192952 |
| DHRS12 | 6.63136785 | -1.772613395 | 0.692546184 | -2.559559833 | 0.010480481 |
| TSC22D1 | 712.6334894 | -0.639848023 | 0.300952441 | -2.126076869 | 0.03349686 |
| CENPT | 25.10444729 | -1.087985448 | 0.553472694 | -1.965743675 | 0.049328227 |
| ACD | 19.35844255 | -1.25589396 | 0.535782013 | -2.344039049 | 0.019076174 |
| NME3 | 15.96747368 | -3.113658504 | 0.908455947 | -3.427418264 | 0.00060935 |
| SLC38A7 | 44.40810752 | -1.068977853 | 0.504883712 | -2.117275379 | 0.034236483 |
| HCFC1R1 | 68.03303937 | -3.549496773 | 1.096978775 | -3.235702324 | 0.001213439 |
| MLYCD | 12.93831259 | 0.887631304 | 0.42327464 | 2.097057611 | 0.035988476 |
| STUB1 | 5.754119038 | -2.486012131 | 1.157981894 | -2.146848879 | 0.031805315 |
| MEFV | 6.628522317 | 1.181117762 | 0.517880659 | 2.280675561 | 0.022567652 |
| CAPN15 | 10.5899605 | -1.619975563 | 0.754686585 | -2.146554073 | 0.0318288 |
| SALL1 | 25.22770049 | -0.95821835 | 0.411111127 | -2.330801302 | 0.019763839 |
| QPRT | 282.833706 | -0.887309274 | 0.443891524 | -1.998932682 | 0.045615638 |
| SLC6A2 | 13.66147117 | 1.70131714 | 0.607205058 | 2.801882357 | 0.00508054 |
| FAH | 36.68872743 | -1.541431002 | 0.543293482 | -2.837197673 | 0.004551143 |
| RPAP1 | 26.64208624 | -1.000944762 | 0.362309811 | -2.762676397 | 0.005732957 |
| PLAT | 99.98716805 | -0.518945436 | 0.22766007 | -2.279474993 | 0.022638845 |
| JPH1 | 17.22122526 | -1.306941311 | 0.525027798 | -2.489280217 | 0.012800203 |
| CCN4 | 8.891407349 | 1.580928602 | 0.734283863 | 2.153021033 | 0.031317026 |
| PYCR3 | 27.46488298 | -1.174924148 | 0.508460038 | -2.310750229 | 0.020846655 |
| KCNN4 | 7.767608488 | -2.842422598 | 0.943272637 | -3.01336272 | 0.002583699 |
| NUCB1 | 17.02312068 | -2.673627165 | 0.768447804 | -3.479256692 | 0.000502807 |
| LIN7B | 20.81103015 | -1.638397508 | 0.604837115 | -2.708824353 | 0.006752208 |
| PPP1R37 | 6.44025116 | 1.376093235 | 0.642609373 | 2.141414819 | 0.032240601 |
| PLEKHJ1 | 58.91940878 | -2.345874202 | 0.857715385 | -2.735026378 | 0.006237528 |
| OAZ1 | 12899.18324 | -1.437029136 | 0.481783615 | -2.982727291 | 0.002856924 |
| YJU2B | 16.94606934 | -1.795224454 | 0.694878243 | -2.583509375 | 0.009780081 |
| TLE5 | 5.770317887 | -2.694018227 | 1.020309169 | -2.640394018 | 0.008280969 |
| SGTA | 64.8799716 | -1.295263963 | 0.622072342 | -2.082175777 | 0.037326417 |
| C19orf53 | 875.2342982 | -1.425519596 | 0.48767647 | -2.923084633 | 0.003465823 |
| TIMM44 | 53.30875477 | -1.189113385 | 0.59161255 | -2.009952942 | 0.04443617 |
| TIMM50 | 271.3412252 | -1.091491749 | 0.508581671 | -2.14614842 | 0.031861139 |
| YJU2 | 20.83620522 | -2.10253033 | 0.653215817 | -3.218737628 | 0.001287562 |
| TBCB | 417.4467786 | -1.120398451 | 0.428823653 | -2.612725402 | 0.008982344 |
| TJP3 | 4.661022896 | 1.479799785 | 0.730137469 | 2.026741331 | 0.042688873 |
| ZNF175 | 138.8928191 | 0.385945072 | 0.187695241 | 2.056232589 | 0.039760102 |
| BCAT2 | 31.44639678 | -1.601218888 | 0.592240215 | -2.703664573 | 0.006857948 |
| PPP2R1A | 381.0882586 | -1.347363423 | 0.53156186 | -2.53472554 | 0.011253546 |
| COPE | 82.70300996 | -3.323154403 | 1.077565888 | -3.083945436 | 0.002042751 |
| KXD1 | 123.3188257 | -2.006022032 | 0.728236775 | -2.754628851 | 0.005875875 |
| CFAP69 | 34.80975902 | -0.705136961 | 0.325286656 | -2.1677402 | 0.030178459 |
| HBP1 | 111.7142892 | 0.519227892 | 0.204277665 | 2.541775142 | 0.01102911 |
| LFNG | 5.869806437 | -1.421339589 | 0.672692323 | -2.112911864 | 0.034608312 |
| ABHD11 | 54.4640877 | -1.388738851 | 0.568069396 | -2.444664088 | 0.014498711 |
| CASP2 | 234.909386 | -1.076992254 | 0.498125833 | -2.162088737 | 0.030611337 |
| HSPB1 | 241.0933687 | -3.647005199 | 1.071217087 | -3.404543528 | 0.000662747 |
| NUDT1 | 54.04584218 | -1.113512124 | 0.361965954 | -3.076289668 | 0.002095941 |
| AIMP2 | 238.6068404 | -1.000290304 | 0.416768698 | -2.400109002 | 0.01639019 |
| IMPDH1 | 28.32719391 | -2.426746894 | 1.020532146 | -2.377923032 | 0.01741046 |
| AGFG2 | 3.807686511 | -3.593697502 | 1.019207525 | -3.525972301 | 0.000421931 |
| AP1S1 | 32.2982098 | -2.213966166 | 0.749715244 | -2.953076096 | 0.003146244 |
| ZNHIT1 | 66.2130962 | -1.342896249 | 0.499515187 | -2.688399239 | 0.00717955 |
| POLD2 | 149.5477493 | -1.359069016 | 0.525860645 | -2.584466113 | 0.009752989 |
| ECM2 | 157.7159887 | -0.709284774 | 0.329365889 | -2.153485825 | 0.031280517 |
| ELAVL2 | 2.895506451 | 2.612244261 | 1.031422198 | 2.53266244 | 0.011319991 |
| ABHD17B | 381.6950778 | 0.464209939 | 0.164604049 | 2.820161118 | 0.004799954 |
| CXCL12 | 12.28749468 | 1.84334351 | 0.546339394 | 3.373989744 | 0.000740871 |
| PALD1 | 10.85736841 | 1.003061034 | 0.493837945 | 2.031154238 | 0.042239351 |
| FBXL15 | 7.713686995 | -2.752853013 | 1.150783598 | -2.39215524 | 0.016749756 |
| CUEDC2 | 25.0633848 | -2.043231239 | 0.845338098 | -2.417058031 | 0.015646522 |
| PFN1 | 388.1903116 | -2.52503439 | 0.856407937 | -2.948401437 | 0.00319422 |
| C17orf75 | 248.6542214 | 0.3394499 | 0.166483589 | 2.038939109 | 0.041456105 |
| RAB5C | 71.40664928 | -1.451231179 | 0.546571324 | -2.655154261 | 0.007927215 |
| PPP1R9B | 14.10446533 | -1.730688293 | 0.692095616 | -2.500649119 | 0.012396593 |
| SGCA | 6.598798255 | -1.400003645 | 0.612941824 | -2.284072629 | 0.022367263 |
| ALOX12 | 3.966037628 | 2.004249354 | 0.953816642 | 2.101294176 | 0.035615151 |
| MYH3 | 7.277699083 | -1.767494518 | 0.720218028 | -2.454110352 | 0.014123363 |
| UNC119 | 55.50760805 | -1.700523896 | 0.539735755 | -3.150660078 | 0.00162902 |
| NFKB1 | 736.0018007 | 0.359924279 | 0.170696902 | 2.10855777 | 0.034982771 |
| AREG | 15.63344081 | -2.255201839 | 0.67935483 | -3.319622881 | 0.000901391 |
| ELP4 | 515.336707 | -0.47996977 | 0.20736769 | -2.314583193 | 0.020635754 |
| ATG2A | 10.74740739 | -1.482212761 | 0.66929702 | -2.214581444 | 0.026788807 |
| PUS3 | 33.07310718 | -0.64506377 | 0.301204919 | -2.141611008 | 0.032224797 |
| NDUFS8 | 54.9179946 | -1.638977096 | 0.831186176 | -1.97185317 | 0.048626371 |
| PSMD9 | 131.609986 | -0.708620574 | 0.345329334 | -2.052013843 | 0.040168317 |
| CAPRIN2 | 1517.579906 | -0.377055515 | 0.176574066 | -2.135395783 | 0.032728695 |
| CAMKK2 | 67.96548347 | -0.842126681 | 0.384209181 | -2.191844241 | 0.028390755 |
| IL23A | 9.473740204 | 1.208148802 | 0.563175789 | 2.145242791 | 0.03193344 |
| ACSS3 | 264.7996387 | 5.1202584 | 0.404389215 | 12.66170859 | 9.64E-37 |
| FOXM1 | 21.12551224 | -0.979404294 | 0.451765125 | -2.1679502 | 0.030162476 |
| SH2B3 | 341.0252426 | -0.250356234 | 0.126696539 | -1.976030574 | 0.048151315 |
| OAS2 | 14.65367276 | -1.561277382 | 0.614824227 | -2.53938819 | 0.011104653 |
| ENDOU | 3.913116025 | 1.912320105 | 0.965157231 | 1.981356035 | 0.04755136 |
| GAPDH | 3325.810976 | -2.215422433 | 0.918034139 | -2.413224454 | 0.015812079 |
| CDCA3 | 56.5253505 | -0.743360403 | 0.346056865 | -2.148087434 | 0.03170681 |
| TPI1 | 3980.731514 | -0.768220944 | 0.357027578 | -2.151713174 | 0.031419952 |
| SPSB2 | 7.260029421 | -1.654375004 | 0.636313595 | -2.599936597 | 0.009324099 |
| LOC105369632 | 7.260029421 | -1.654375004 | 0.636313595 | -2.599936597 | 0.009324099 |
| E2F3 | 163.6790669 | 0.723541414 | 0.200619104 | 3.606542942 | 0.000310304 |
| WASF1 | 248.8977355 | 0.412911969 | 0.176816591 | 2.335255794 | 0.019530062 |
| SLC17A2 | 5.236179963 | 2.382729829 | 0.785661466 | 3.032769117 | 0.002423209 |
| HECA | 1401.178768 | 0.456938642 | 0.223582839 | 2.043710707 | 0.040982135 |
| TBP | 126.1825249 | -0.551972831 | 0.183012574 | -3.016037749 | 0.002561014 |
| DNPH1 | 73.50098333 | -1.546955573 | 0.476593402 | -3.245860238 | 0.001170963 |
| SLC29A1 | 68.32298195 | -0.950046117 | 0.453948094 | -2.09285187 | 0.03636238 |
| PCDHB7 | 4.193535612 | -3.780738628 | 1.022897473 | -3.696107113 | 0.000218931 |
| STC2 | 66.96691384 | 0.518665412 | 0.228226876 | 2.272586914 | 0.023051085 |
| UNC5A | 4.040408774 | 2.048492871 | 0.868259023 | 2.359310776 | 0.018308916 |
| OGG1 | 16.00511687 | -1.194876686 | 0.564298755 | -2.117454054 | 0.034221331 |
| GRK7 | 14.79800949 | 1.09163795 | 0.484241668 | 2.254324696 | 0.024175749 |
| CYB561D2 | 27.40914576 | -2.072935193 | 0.611024744 | -3.392555233 | 0.00069244 |
| CBLB | 168.1002678 | -0.575149292 | 0.257163359 | -2.236513374 | 0.025318162 |
| PODXL2 | 4.518058261 | -2.413424647 | 1.108398443 | -2.177398086 | 0.029450878 |
| KLHL18 | 64.99528535 | -0.513792971 | 0.211582899 | -2.428329382 | 0.01516856 |
| GORASP1 | 43.31807854 | 0.840876584 | 0.245944051 | 3.418975091 | 0.000628575 |
| EIF1B | 459.2386895 | 0.324208139 | 0.164868842 | 1.966460939 | 0.04924539 |
| FAHD2A | 97.75492657 | -1.229871252 | 0.562356485 | -2.186995766 | 0.028742833 |
| MPV17 | 173.4506676 | -0.851409435 | 0.263008567 | -3.237192782 | 0.001207118 |
| ITGA4 | 21.2918004 | 0.768494268 | 0.370572319 | 2.07380376 | 0.03809754 |
| IFIH1 | 24.14531994 | 0.839061607 | 0.38424478 | 2.183664296 | 0.028986925 |
| NDUFS7 | 35.10634519 | -2.871768018 | 1.076288934 | -2.6682129 | 0.007625592 |
| PCGF1 | 58.96937828 | -0.799974815 | 0.331510071 | -2.413123714 | 0.01581645 |
| TACR1 | 17.48046862 | 1.030791584 | 0.469001854 | 2.19784117 | 0.027960426 |
| ACADL | 7.16914019 | -1.874346971 | 0.872965873 | -2.147102228 | 0.031785144 |
| FN1 | 4795.414456 | -0.452782332 | 0.191997767 | -2.358268739 | 0.018360396 |
| IL1RL1 | 183.5304119 | 0.536141689 | 0.246557696 | 2.17450803 | 0.029667003 |
| FHL2 | 4096.036134 | -0.34745319 | 0.144993134 | -2.396342367 | 0.016559615 |
| ODC1 | 5430.126926 | 0.479528253 | 0.190034688 | 2.523372228 | 0.011623529 |
| SPR | 47.00783372 | -0.955415666 | 0.426148409 | -2.241978721 | 0.024962748 |
| ANGPTL1 | 44.77149681 | -1.437791961 | 0.313065221 | -4.59262756 | 4.38E-06 |
| FAM20B | 403.8359401 | 0.384454925 | 0.172577956 | 2.227717448 | 0.025899363 |
| TCEANC2 | 62.9456988 | 0.633512666 | 0.208032564 | 3.04525722 | 0.002324814 |
| ERRFI1 | 1018.220341 | 0.386923479 | 0.128155247 | 3.01917782 | 0.002534617 |
| MECR | 60.04502001 | -1.395955417 | 0.484065385 | -2.883815821 | 0.003928885 |
| SRM | 48.22857948 | -2.275716829 | 0.681912849 | -3.337254656 | 0.000846104 |
| CTH | 180.029769 | 0.623132657 | 0.229177079 | 2.719000781 | 0.006547945 |
| OLFML3 | 14.46983644 | -1.652456591 | 0.523869736 | -3.154327263 | 0.001608686 |
| CD2 | 4.613031774 | -3.792619064 | 1.337779791 | -2.835009983 | 0.004582425 |
| TMEM9 | 249.0599213 | -1.194963973 | 0.470332313 | -2.540680154 | 0.011063708 |
| MRPS15 | 1010.655988 | -0.647375411 | 0.323181223 | -2.003134355 | 0.045162869 |
| RLF | 485.7056183 | 0.430811452 | 0.161063094 | 2.674799314 | 0.0074774 |
| AKT3 | 110.3370377 | 0.469605057 | 0.194243701 | 2.417607652 | 0.015622912 |
| SLAMF1 | 3.43143377 | 2.122629799 | 0.864389059 | 2.455641677 | 0.014063331 |
| HMGCL | 111.7222517 | -1.028850428 | 0.422661494 | -2.4342185 | 0.014923983 |
| ID3 | 394.6443433 | -2.407825862 | 0.745942715 | -3.227896476 | 0.001247041 |
| CDC20 | 380.1303163 | -0.934267288 | 0.461554864 | -2.024173855 | 0.042952267 |
| MPL | 6.049988533 | 1.355640969 | 0.637589578 | 2.126196878 | 0.03348687 |
| ATP6V0B | 3167.671157 | -1.548545668 | 0.650463145 | -2.380681642 | 0.017280639 |
| B4GALT2 | 30.57321118 | -1.766447414 | 0.669010745 | -2.640387209 | 0.008281135 |
| ERI3 | 57.78913901 | -3.332673874 | 1.114151032 | -2.991222715 | 0.002778628 |
| CNN3 | 480.2804918 | -0.751899191 | 0.37424794 | -2.009093735 | 0.044527194 |
| MUC5B | 15.19005987 | 1.237856391 | 0.444639353 | 2.783955992 | 0.005370032 |
| IRAG2 | 17.02706219 | 0.976674076 | 0.466892126 | 2.091862385 | 0.036450828 |
| UBE3D | 118.2801208 | -0.410036506 | 0.200379557 | -2.04629909 | 0.040726953 |
| PHF3 | 1468.335412 | 0.271767436 | 0.12103942 | 2.245280384 | 0.024750139 |
| EEF2KMT | 28.97202223 | -1.522597012 | 0.718523555 | -2.119063463 | 0.034085104 |
| PTPA | 41.75575463 | -1.408634426 | 0.497926677 | -2.828999711 | 0.004669374 |
| NR4A3 | 111.1229242 | 0.954524893 | 0.373336904 | 2.55673865 | 0.010565857 |
| DENND1A | 49.16668978 | -0.550690575 | 0.277369459 | -1.985404507 | 0.047099485 |
| C19orf25 | 27.68812902 | -1.303121165 | 0.601609188 | -2.166059281 | 0.030306657 |
| MLH3 | 380.8451255 | 0.419898314 | 0.170985715 | 2.455750841 | 0.01405906 |
| DNMT3A | 31.57359469 | 0.957233267 | 0.307113161 | 3.11687478 | 0.001827792 |
| EPCAM | 97.5658767 | 0.645906704 | 0.322487848 | 2.002886953 | 0.045189424 |
| DUSP1 | 355.7615393 | -0.558278094 | 0.251096217 | -2.223363223 | 0.026191318 |
| MSANTD2 | 89.98868132 | -0.826057164 | 0.278978107 | -2.961010713 | 0.003066313 |
| HSPH1 | 8143.413308 | 0.298394679 | 0.11083464 | 2.692251088 | 0.007097149 |
| PPP3CC | 460.211216 | 0.338036399 | 0.157061819 | 2.152250636 | 0.03137762 |
| AKAP1 | 82.06425343 | -0.504765669 | 0.188113504 | -2.68330375 | 0.007289875 |
| BCL2L14 | 4.116411041 | 2.195817254 | 0.78919542 | 2.782349211 | 0.005396694 |
| DPPA4 | 4.556056663 | -1.342110991 | 0.678886736 | -1.976929169 | 0.048049637 |
| TMEM39B | 29.14807086 | -1.632778245 | 0.57751864 | -2.82723038 | 0.004695254 |
| CXCR4 | 19.36135298 | 1.711386295 | 0.535101798 | 3.198244335 | 0.001382671 |
| RASL11A | 24.12905821 | -1.437633163 | 0.652592214 | -2.202957886 | 0.027597717 |
| OCRL | 50.70123968 | -0.482913144 | 0.24459838 | -1.974310479 | 0.04834645 |
| BBS9 | 36.83132434 | 0.804225791 | 0.311447848 | 2.582216561 | 0.009816796 |
| OCM | 2.82533185 | -3.63872961 | 1.295604743 | -2.808518284 | 0.004977006 |
| NUDT10 | 5.575570338 | 1.419931286 | 0.678455592 | 2.092887586 | 0.036359191 |
| ZWINT | 294.4473721 | -0.790061478 | 0.332437897 | -2.376568632 | 0.01747451 |
| ACADS | 4.821111998 | -1.820860888 | 0.887765111 | -2.051061555 | 0.040260953 |
| SSPN | 47.29573466 | 0.929156605 | 0.365749769 | 2.540416112 | 0.011072065 |
| OPTN | 2145.282787 | 0.332884705 | 0.143641602 | 2.317467228 | 0.020478294 |
| ATG101 | 18.67734915 | -2.700344009 | 0.72313329 | -3.734227212 | 0.000188292 |
| TUBA1B | 580.2158663 | -1.708555131 | 0.858227761 | -1.990794529 | 0.046503481 |
| B9D2 | 52.87100384 | -1.787183177 | 0.636873009 | -2.806184518 | 0.005013198 |
| GCNT7 | 3.05524286 | -2.734107292 | 0.969004937 | -2.821561776 | 0.004779043 |
| PI3 | 58.23665826 | 1.168424618 | 0.422613284 | 2.764760744 | 0.005696456 |
| KCNS1 | 6.267883168 | 1.217458451 | 0.593186096 | 2.052405576 | 0.040130263 |
| SDC4 | 456.3964083 | -0.961917467 | 0.46947941 | -2.048902349 | 0.040471665 |
| NCOA3 | 2043.083512 | 0.310403542 | 0.147373273 | 2.106240397 | 0.035183476 |
| PIGT | 103.2580382 | -0.841843001 | 0.356597402 | -2.360765939 | 0.018237237 |
| NAGK | 301.6143869 | -0.789498995 | 0.367017039 | -2.151123549 | 0.031466449 |
| USP22 | 68.73770668 | -1.898871948 | 0.66244248 | -2.866470681 | 0.004150766 |
| ZNF391 | 52.24195768 | -0.739371689 | 0.341878672 | -2.162672753 | 0.030566358 |
| H2BC11 | 36.84543947 | -1.113531821 | 0.268936813 | -4.140496076 | 3.47E-05 |
| TBCC | 184.4281738 | -0.782728348 | 0.359767873 | -2.17564826 | 0.029581572 |
| MEA1 | 315.4854678 | -1.001189363 | 0.357660542 | -2.799272623 | 0.005121787 |
| KLHL31 | 13.0152485 | -1.543604027 | 0.564838298 | -2.732824654 | 0.006279375 |
| EREG | 43.4646263 | 0.793428227 | 0.375827735 | 2.111148683 | 0.034759534 |
| BBS2 | 31.33566675 | -1.078122333 | 0.329741446 | -3.269599089 | 0.001077 |
| MIF4GD | 79.51665611 | -1.242634191 | 0.530487181 | -2.342439623 | 0.019158133 |
| CHCHD5 | 145.6841537 | -1.062711683 | 0.481366899 | -2.207695804 | 0.027265486 |
| SLC25A23 | 21.39585651 | -1.473439046 | 0.591769366 | -2.489887327 | 0.01277836 |
| ALKBH7 | 67.78702691 | -2.022592513 | 0.833443908 | -2.426789005 | 0.015233112 |
| CLPP | 87.21470108 | -1.162999105 | 0.521167163 | -2.231527976 | 0.025646177 |
| CD70 | 47.12426399 | -1.911821152 | 0.637758541 | -2.997719401 | 0.00272008 |
| TRIP10 | 25.40444456 | -1.337247643 | 0.59134964 | -2.261348538 | 0.023737683 |
| OPA3 | 22.20763191 | -0.885210477 | 0.410590365 | -2.155945565 | 0.031087916 |
| FOXA2 | 4.699780975 | 2.171690773 | 0.916062458 | 2.370679808 | 0.017755405 |
| RBCK1 | 343.4653901 | -1.09347739 | 0.510430013 | -2.142267035 | 0.032172 |
| SNRPB | 221.5670751 | -2.065351822 | 0.748216081 | -2.760368126 | 0.005773626 |
| MRPS26 | 274.5289983 | -2.29533417 | 0.792765667 | -2.895350123 | 0.003787358 |
| ID1 | 2177.857924 | -1.018148956 | 0.415815693 | -2.448558277 | 0.014342923 |
| PLAGL2 | 31.06664796 | -0.538066464 | 0.270214223 | -1.991258853 | 0.046452437 |
| UROD | 280.9634186 | -0.887302525 | 0.409822243 | -2.165091186 | 0.030380703 |
| CAPNS1 | 100.7352559 | -1.095007242 | 0.500076321 | -2.189680248 | 0.028547435 |
| PRDX5 | 382.870045 | -1.173523504 | 0.462733272 | -2.536068992 | 0.011210465 |
| IRF3 | 31.00995464 | -1.763950174 | 0.736349847 | -2.395532751 | 0.016596232 |
| ELK1 | 6.336653485 | 1.721541535 | 0.620208367 | 2.775747034 | 0.005507504 |
| TIMM17B | 37.59720733 | -4.451359992 | 1.497232869 | -2.973057888 | 0.002948488 |
| L3HYPDH | 67.89046919 | -0.867374155 | 0.275805513 | -3.144876066 | 0.001661572 |
| AIF1L | 10.55687474 | 1.1658533 | 0.552126189 | 2.111570368 | 0.034723316 |
| ZC4H2 | 17.36130922 | -0.935348363 | 0.413058216 | -2.264446818 | 0.023546647 |
| OMD | 88.64467425 | -1.090248897 | 0.429247613 | -2.539906719 | 0.011088204 |
| BEST3 | 23.09033282 | -0.893860867 | 0.392794825 | -2.275643187 | 0.022867374 |
| TAS2R4 | 11.22807105 | -1.140230765 | 0.548443664 | -2.079029881 | 0.037614604 |
| PIN1 | 158.4997309 | -3.473343873 | 0.934592362 | -3.716426555 | 0.00020206 |
| SIN3B | 36.01921516 | -0.981473702 | 0.28627136 | -3.428473263 | 0.000606986 |
| EPS15L1 | 44.76234906 | -1.071223153 | 0.317199011 | -3.377132701 | 0.000732457 |
| GFER | 13.38383243 | -1.86218343 | 0.944911053 | -1.970749971 | 0.048752482 |
| PKMYT1 | 10.65210871 | -1.635292625 | 0.72345304 | -2.260399134 | 0.02379649 |
| TUBA4A | 329.3869996 | -1.57387082 | 0.695484558 | -2.262984566 | 0.023636641 |
| DGCR6L | 293.5152413 | -1.840142231 | 0.82791981 | -2.222609253 | 0.02624216 |
| SDF2L1 | 132.6148793 | -1.918092491 | 0.719120962 | -2.667273787 | 0.007646935 |
| FLNC | 3.262948263 | -2.304658337 | 1.026526972 | -2.245102564 | 0.024761549 |
| HOXD3 | 6.020770665 | 1.391008348 | 0.653604874 | 2.128209876 | 0.033319686 |
| MYO5C | 40.82155719 | -0.904276369 | 0.307074858 | -2.944807578 | 0.003231556 |
| ISLR | 4.47131464 | 1.867222348 | 0.930112502 | 2.007523116 | 0.044693992 |
| TPH1 | 34.23200123 | -0.690131566 | 0.32031485 | -2.154541281 | 0.031197748 |
| MPDU1 | 44.55866111 | -1.39249105 | 0.679597753 | -2.048993018 | 0.040462798 |
| DTD2 | 8.177121843 | -1.798714098 | 0.600349743 | -2.996110384 | 0.002734474 |
| NXNL2 | 21.92523821 | -0.808984412 | 0.367152306 | -2.203402779 | 0.027566373 |
| FAM98C | 9.040370402 | -1.336870596 | 0.587188201 | -2.27673273 | 0.022802191 |
| BST2 | 55.40575124 | -1.73108168 | 0.578706812 | -2.991293079 | 0.002777987 |
| NSUN5 | 25.71228383 | -1.426540709 | 0.713818535 | -1.998464089 | 0.045666369 |
| PGLS | 157.25143 | -1.691341846 | 0.839280505 | -2.015228324 | 0.043880731 |
| LSM4 | 992.0452829 | -1.205587694 | 0.510339066 | -2.362326879 | 0.018160621 |
| SULT4A1 | 8.704238121 | -1.624695632 | 0.617202027 | -2.632356281 | 0.008479489 |
| UBE2M | 79.30088808 | -2.146671746 | 0.815392133 | -2.63268636 | 0.008471253 |
| METTL26 | 156.0994698 | -2.349474447 | 0.805491015 | -2.916822665 | 0.003536168 |
| YIPF2 | 7.048114048 | -2.149803228 | 1.072812334 | -2.003894959 | 0.045081314 |
| CCDC62 | 107.5551504 | -0.5691996 | 0.222533651 | -2.557813609 | 0.010533253 |
| EIF3G | 259.888998 | -1.692504009 | 0.804695212 | -2.103285796 | 0.035440794 |
| SLC35D2 | 37.2284075 | -0.702094735 | 0.307481395 | -2.283373068 | 0.022408402 |
| NR1H2 | 34.64640977 | -1.263268867 | 0.618470606 | -2.042568966 | 0.041095126 |
| TUBG1 | 307.494561 | -2.18363934 | 0.89433599 | -2.441631963 | 0.014621044 |
| VPS25 | 1000.539625 | -1.023239013 | 0.482638836 | -2.120092576 | 0.033998239 |
| NINJ1 | 110.7857632 | -2.02665454 | 0.939798489 | -2.15647776 | 0.031046379 |
| KRT33B | 3.034898295 | -2.248876568 | 1.087481346 | -2.067967948 | 0.038643037 |
| RHPN2 | 483.1783567 | 0.31818493 | 0.155335375 | 2.04837391 | 0.040523376 |
| LGALS3 | 643.4385049 | -1.253495039 | 0.44591153 | -2.811084612 | 0.00493748 |
| SLC6A11 | 5.148233224 | -2.738722984 | 0.894337041 | -3.062294034 | 0.002196476 |
| EIF5A | 165.3791276 | -1.566719385 | 0.745380824 | -2.101904602 | 0.035561633 |
| FLOT2 | 57.01867628 | -1.650638127 | 0.660289203 | -2.499871449 | 0.012423838 |
| PCED1A | 7.912109147 | -1.970066108 | 0.76133927 | -2.587632329 | 0.009663807 |
| NXT1 | 413.067201 | -0.801657021 | 0.32100037 | -2.497371019 | 0.012511797 |
| KHDC4 | 1086.958913 | -0.350843503 | 0.176637119 | -1.98623882 | 0.047006812 |
| FCRL2 | 6.387717962 | 1.369026013 | 0.638544327 | 2.143979603 | 0.032034521 |
| TESMIN | 14.48145671 | 1.472725387 | 0.380339572 | 3.872132943 | 0.000107887 |
| KANK4 | 9.120929437 | 1.454599872 | 0.472453114 | 3.07882376 | 0.002078196 |
| ANGPTL3 | 50.80454685 | -0.964533407 | 0.457541642 | -2.108077865 | 0.035024255 |
| SYT4 | 3.470213471 | 2.530657375 | 1.239484236 | 2.041701944 | 0.041181106 |
| MYH10 | 82.27684033 | -0.559032735 | 0.24656753 | -2.267260151 | 0.023374338 |
| PEMT | 17.53225163 | -1.557861183 | 0.766844774 | -2.031520897 | 0.042202182 |
| BTBD2 | 8.078486473 | -1.94216847 | 0.850638583 | -2.283188782 | 0.022419251 |
| WDR74 | 26.38701492 | -1.543088053 | 0.637306613 | -2.421264777 | 0.015466607 |
| TEX15 | 6.284907927 | -2.365829061 | 0.73810826 | -3.205260246 | 0.001349404 |
| LOXL2 | 251.8185235 | -0.923673667 | 0.407076258 | -2.269043327 | 0.023265691 |
| CD180 | 8.162119831 | 1.655789102 | 0.587169286 | 2.819951831 | 0.004803086 |
| CHL1 | 11.06879419 | 1.13600493 | 0.520947036 | 2.180653409 | 0.029209062 |
| SYT6 | 4.642506494 | 2.079663009 | 0.737152463 | 2.821211504 | 0.004784265 |
| NGF | 44.34556555 | -1.169776448 | 0.422800716 | -2.766732421 | 0.005662121 |
| RSAD2 | 49.67779064 | 1.154219211 | 0.35036808 | 3.294304698 | 0.000986655 |
| CFHR4 | 8.049726335 | 1.962652446 | 0.760745142 | 2.579907958 | 0.009882665 |
| KCTD1 | 167.7261356 | 0.374605826 | 0.189427989 | 1.97756323 | 0.047978001 |
| SOX5 | 9.463585288 | 1.509608999 | 0.61067363 | 2.472038951 | 0.013434488 |
| RTL8C | 134.5993609 | -1.591950893 | 0.508274909 | -3.132066651 | 0.001735805 |
| AGO4 | 159.8518485 | 0.383857232 | 0.175035053 | 2.193030628 | 0.028305172 |
| DTNA | 18.42524725 | -0.949396901 | 0.410311706 | -2.31384308 | 0.020676331 |
| SLC43A3 | 39.66763051 | 0.681720622 | 0.299808293 | 2.273855119 | 0.022974699 |
| GOLM1 | 660.691462 | 0.312095988 | 0.128444489 | 2.429812218 | 0.015106647 |
| DTX1 | 5.175276185 | -1.869680423 | 0.805162705 | -2.322115034 | 0.020226742 |
| CD36 | 5.855762302 | -2.385032461 | 0.891946294 | -2.673964204 | 0.007496045 |
| PRRG4 | 109.6236195 | 0.697850922 | 0.215671437 | 3.235713224 | 0.001213392 |
| LACRT | 3.013861472 | 2.804204025 | 1.364175003 | 2.055604317 | 0.039820671 |
| CDK4 | 41.57990292 | -2.255854166 | 1.013965211 | -2.22478458 | 0.026095703 |
| AFG1L | 92.7375004 | 0.542943718 | 0.222126439 | 2.444300287 | 0.014513341 |
| HEY2 | 5.900137975 | -1.923842924 | 0.847828265 | -2.269142235 | 0.023259678 |
| KCNK1 | 1751.279852 | 0.476771815 | 0.127164706 | 3.749246404 | 0.000177367 |
| NTPCR | 261.0867952 | -0.803356931 | 0.371167313 | -2.164406466 | 0.030433168 |
| TAF5L | 69.22524561 | 0.854248021 | 0.339615715 | 2.515337139 | 0.011891861 |
| DOCK10 | 230.4866635 | -0.514864054 | 0.194222024 | -2.650904594 | 0.008027651 |
| PLXNC1 | 42.97836122 | -0.804352214 | 0.267364278 | -3.008450568 | 0.002625835 |
| PHF11 | 481.5273277 | -0.500082451 | 0.158797273 | -3.149187901 | 0.001637249 |
| IGF2BP3 | 98.3096192 | 0.562723452 | 0.241739365 | 2.327810582 | 0.019922164 |
| IL6 | 93.49216811 | 0.588507467 | 0.238451519 | 2.468038237 | 0.013585581 |
| ZFHX2 | 5.725889639 | 1.51185976 | 0.737235799 | 2.050713982 | 0.040294809 |
| MYCBPAP | 9.658066259 | 1.810844502 | 0.644365805 | 2.810274052 | 0.004949933 |
| IMP4 | 366.9016641 | -0.557679494 | 0.278301235 | -2.003869995 | 0.045083988 |
| ECPAS | 1422.617804 | 0.357507297 | 0.10046341 | 3.558582145 | 0.000372862 |
| ANGPTL2 | 5.538994482 | 1.629391005 | 0.710077718 | 2.294665731 | 0.021752288 |
| DPM2 | 693.7457539 | -2.230786002 | 0.839898904 | -2.656017281 | 0.007906956 |
| IL33 | 69.67786928 | -1.633475849 | 0.375844566 | -4.346147308 | 1.39E-05 |
| GRHPR | 126.3298798 | -0.776038454 | 0.363251232 | -2.1363684 | 0.032649398 |
| CNPY3 | 77.05255615 | -1.758643936 | 0.784688136 | -2.241201129 | 0.02501305 |
| YIPF3 | 82.94482706 | -1.20427602 | 0.485702988 | -2.479449479 | 0.013158537 |
| MGARP | 10.7237478 | 1.041448575 | 0.484343023 | 2.150229332 | 0.031537079 |
| SYTL2 | 127.5506811 | -0.587385044 | 0.240673587 | -2.440587899 | 0.014663377 |
| FXYD6 | 4.773105678 | 2.082382317 | 0.705935428 | 2.949819821 | 0.003179593 |
| TMPRSS13 | 5.714669499 | 1.876765845 | 0.893271882 | 2.101001814 | 0.035640807 |
| MAP2K5 | 38.78502384 | -0.668274969 | 0.263047137 | -2.540514132 | 0.011068962 |
| IFI44L | 11.68673597 | -1.453862982 | 0.636935957 | -2.282588956 | 0.022454592 |
| MYPN | 44.24599501 | -0.66146367 | 0.298633043 | -2.214971467 | 0.026762023 |
| HCN4 | 4.281198116 | 1.849087649 | 0.803359641 | 2.301693482 | 0.021352464 |
| PDE5A | 107.8592277 | -0.742454996 | 0.235647044 | -3.150707863 | 0.001628753 |
| CCNG2 | 160.4894101 | 0.528310868 | 0.231960098 | 2.27759374 | 0.022750794 |
| PEX5 | 7.511900788 | 1.383814242 | 0.602320728 | 2.29747073 | 0.021591931 |
| PPFIA2 | 6.882519987 | -1.180190429 | 0.581108568 | -2.030929318 | 0.042262165 |
| LUM | 25.5942272 | 1.048571129 | 0.51573389 | 2.033163129 | 0.042036045 |
| SDSL | 7.271135219 | -2.162424213 | 0.756805798 | -2.857303972 | 0.004272565 |
| VPS33A | 164.7224936 | 0.597276959 | 0.183686034 | 3.251618777 | 0.001147498 |
| VPS37B | 11.48370608 | -2.005160521 | 0.881413081 | -2.274938464 | 0.022909622 |
| SRRM4 | 10.90863186 | 1.205024034 | 0.5646579 | 2.134078055 | 0.032836392 |
| SLC38A6 | 168.3859201 | -0.669228446 | 0.300276883 | -2.22870452 | 0.025833571 |
| SLC24A4 | 21.72175537 | 0.84179797 | 0.316697473 | 2.658050799 | 0.007859405 |
| SLC12A6 | 243.984316 | -0.600949921 | 0.283036839 | -2.123221567 | 0.03373529 |
| SLC27A2 | 25.1982047 | 1.308170449 | 0.394381388 | 3.317018727 | 0.000909835 |
| COMMD4 | 368.525314 | -0.861417396 | 0.363418794 | -2.370316039 | 0.017772886 |
| ABCC12 | 18.03879888 | 1.100095458 | 0.487926824 | 2.254632056 | 0.024156434 |
| MAP1LC3B | 2947.452851 | 0.282060261 | 0.117901601 | 2.392336131 | 0.016741502 |
| DEF8 | 392.3741075 | -0.671324161 | 0.301018633 | -2.230174774 | 0.025735842 |
| RANBP10 | 60.13531562 | -0.596132443 | 0.297301621 | -2.005143603 | 0.044947697 |
| LRRC46 | 12.30269113 | -1.495608595 | 0.692195773 | -2.160672824 | 0.030720622 |
| G6PC3 | 49.83121214 | -1.806614528 | 0.65004051 | -2.779233757 | 0.00544873 |
| ANAPC11 | 4174.721288 | -0.570970344 | 0.289332894 | -1.973402802 | 0.048449689 |
| NARF | 212.2405177 | -1.1673534 | 0.547797626 | -2.1309939 | 0.033089645 |
| RNF157 | 12.80110679 | -0.940706767 | 0.473952234 | -1.984813446 | 0.047165231 |
| CBX4 | 5.629911604 | -1.482762404 | 0.690475531 | -2.147451051 | 0.03175739 |
| CBLN2 | 8.884803692 | 1.771606931 | 0.512320284 | 3.458006616 | 0.000544188 |
| PFKL | 70.5783548 | -1.875240686 | 0.633017512 | -2.962383586 | 0.003052672 |
| SH3GL1 | 18.29744896 | -1.638423326 | 0.778767359 | -2.103867487 | 0.035390008 |
| DPP9 | 129.0451867 | -1.122834993 | 0.570832752 | -1.967012208 | 0.049181803 |
| IFITM3 | 640.9559827 | -1.383921629 | 0.451653644 | -3.064121472 | 0.002183102 |
| EMP3 | 229.7816309 | -1.567916126 | 0.513090775 | -3.055825993 | 0.002244415 |
| ERVK3-1 | 147.8342538 | -0.336921644 | 0.155546832 | -2.166046328 | 0.030307647 |
| TIMM29 | 17.08986668 | 0.782577552 | 0.360419769 | 2.171294752 | 0.029908899 |
| NOSIP | 613.9768686 | -2.10227725 | 0.852969638 | -2.464656602 | 0.013714462 |
| SH3BGRL3 | 1030.855046 | -2.793222254 | 0.907741255 | -3.07711282 | 0.002090162 |
| BCL10 | 393.3408334 | 0.490570191 | 0.239342372 | 2.049658766 | 0.040397741 |
| MRPL24 | 108.2477739 | -0.86793244 | 0.361561923 | -2.400508419 | 0.016372314 |
| RGS16 | 6.405925601 | 2.546251539 | 0.906603507 | 2.808561316 | 0.00497634 |
| HMCN1 | 37.33687357 | -0.698682331 | 0.318684026 | -2.192398347 | 0.028350756 |
| RGL1 | 49.68158232 | 0.78318727 | 0.262433776 | 2.984323436 | 0.002842062 |
| SF3B4 | 28.57911194 | -2.439878238 | 0.984225738 | -2.478982355 | 0.013175782 |
| PI4KB | 26.67386725 | -1.253031873 | 0.510192413 | -2.455998642 | 0.014049369 |
| ATP8B2 | 23.6199056 | -1.718344609 | 0.660050161 | -2.603354578 | 0.009231642 |
| ADAM15 | 6.829899295 | -1.332613617 | 0.596786188 | -2.23298334 | 0.025550043 |
| NUP210L | 12.5403709 | -1.572360797 | 0.536541357 | -2.930549114 | 0.003383635 |
| RIT1 | 276.9002611 | 0.504969762 | 0.219093206 | 2.304817079 | 0.02117682 |
| GUK1 | 774.3156526 | -2.106704251 | 0.795723099 | -2.647534366 | 0.008108112 |
| ABHD1 | 3.147294387 | -3.00379703 | 1.141867464 | -2.63060042 | 0.008523418 |
| RABL2A | 11.36409258 | -1.16088532 | 0.510174542 | -2.275466972 | 0.022877932 |
| AFF3 | 78.61954472 | -0.494711627 | 0.217286218 | -2.276774071 | 0.022799721 |
| ABCA12 | 17.4392907 | -1.385458609 | 0.626949827 | -2.209839686 | 0.02711629 |
| TRPM8 | 14.87088721 | 0.940439259 | 0.429417094 | 2.190036851 | 0.028521564 |
| GRIP2 | 8.780316466 | 1.069415984 | 0.46022346 | 2.323688552 | 0.020142192 |
| EAF1 | 560.9140959 | 0.322664157 | 0.15033991 | 2.14623088 | 0.031854563 |
| GOLGA4 | 5084.91336 | 0.298051029 | 0.120328043 | 2.476987258 | 0.013249663 |
| STXBP5L | 11.03329536 | -1.87975719 | 0.667869705 | -2.814556754 | 0.004884455 |
| SLC10A6 | 3.80786718 | 2.09992527 | 1.040648913 | 2.017899836 | 0.043601694 |
| SNCA | 19.80814303 | 1.441012888 | 0.427051401 | 3.374331253 | 0.000739953 |
| TBCK | 215.9100899 | -0.378248225 | 0.188539106 | -2.006205673 | 0.044834309 |
| ADAMTS16 | 9.940306419 | -1.360994333 | 0.624184995 | -2.180434236 | 0.029225289 |
| GZMA | 3.38310792 | 2.260405215 | 0.961039772 | 2.352041279 | 0.018670703 |
| LIX1 | 2.871651595 | -3.666756651 | 1.22104205 | -3.00297328 | 0.002673559 |
| PCYOX1L | 12.11233449 | 0.950278906 | 0.48243769 | 1.969744335 | 0.048867679 |
| NHP2 | 197.2166291 | -1.156683434 | 0.474293204 | -2.438751861 | 0.014738084 |
| TBC1D7 | 295.4501854 | 0.33074593 | 0.156520639 | 2.113113855 | 0.034591024 |
| TBC1D7-LOC100130357 | 295.4501854 | 0.33074593 | 0.156520639 | 2.113113855 | 0.034591024 |
| CYP39A1 | 30.79017146 | -0.970772097 | 0.361234362 | -2.687374731 | 0.007201611 |
| CLVS2 | 6.423971399 | 2.218764952 | 0.750552411 | 2.956175903 | 0.003114794 |
| SLC22A3 | 31.23082393 | -0.923216812 | 0.385825377 | -2.392835895 | 0.016718717 |
| C7orf50 | 94.65201568 | -3.045986987 | 1.024776397 | -2.972343035 | 0.002955363 |
| ASB15 | 7.485545716 | -2.795286808 | 0.822243517 | -3.399585097 | 0.000674882 |
| CLTRN | 9.98126939 | -1.087038273 | 0.529321171 | -2.053645939 | 0.040009972 |
| SYTL5 | 18.95638199 | 1.277100344 | 0.43135208 | 2.960691282 | 0.003069494 |
| GPC3 | 7.391967976 | 1.987112379 | 0.826563064 | 2.404066267 | 0.016213834 |
| MCPH1 | 454.7426369 | 0.360204554 | 0.130206165 | 2.766417049 | 0.0056676 |
| CSGALNACT1 | 16.81542997 | 0.920521173 | 0.42838356 | 2.148824695 | 0.031648299 |
| SLC25A37 | 267.4075648 | -0.677090912 | 0.300410278 | -2.253887307 | 0.024203258 |
| TERF1 | 1648.53271 | 0.369065242 | 0.16810949 | 2.195386123 | 0.028135912 |
| SLC39A4 | 45.87346338 | -1.422446524 | 0.574992177 | -2.473853699 | 0.013366442 |
| NAPRT | 17.11267399 | -1.744039266 | 0.798009805 | -2.185486013 | 0.02885323 |
| NTRK2 | 30.27721059 | -1.336137944 | 0.610640422 | -2.188092856 | 0.028662839 |
| PTGES2 | 18.91706159 | -1.390769339 | 0.487531917 | -2.852673415 | 0.004335315 |
| NTMT1 | 99.42014645 | -2.400545199 | 0.843061223 | -2.847415031 | 0.004407586 |
| SH3GLB2 | 33.1257911 | -1.340607334 | 0.573026801 | -2.339519429 | 0.019308567 |
| MIGA2 | 12.75165859 | -1.409091478 | 0.626578853 | -2.248865361 | 0.024521063 |
| PAXX | 46.65580108 | -2.157741933 | 0.878142014 | -2.457167404 | 0.014003741 |
| RGR | 9.136294722 | 1.769333182 | 0.605878426 | 2.920277577 | 0.003497197 |
| HABP2 | 4.262501072 | 1.735085862 | 0.852677141 | 2.034868507 | 0.041864106 |
| PLEKHS1 | 7.617919941 | 1.558704194 | 0.60614145 | 2.571518897 | 0.010125349 |
| ZNF214 | 27.18838566 | -1.086733561 | 0.402332967 | -2.701080079 | 0.00691147 |
| CSTPP1 | 75.75566949 | -0.701544356 | 0.325579569 | -2.154755469 | 0.031180975 |
| SESN3 | 9.475551889 | -2.443405588 | 0.589841301 | -4.142479652 | 3.44E-05 |
| TAGLN | 50.13894225 | -2.198299362 | 0.871632385 | -2.522048745 | 0.011667354 |
| TRPT1 | 4.340578727 | -2.727023636 | 1.106691954 | -2.464121679 | 0.013734947 |
| TM7SF2 | 3.611037897 | -2.919037736 | 1.252772522 | -2.330062071 | 0.019802871 |
| TMEM219 | 527.0487882 | -1.001460681 | 0.460730212 | -2.173637967 | 0.029732335 |
| MMP3 | 292.474526 | 1.744623925 | 0.37371368 | 4.668343757 | 3.04E-06 |
| MPP7 | 76.01520156 | 0.697928184 | 0.288401929 | 2.419984456 | 0.015521171 |
| PPIAP26 | 3.762822065 | 3.252101685 | 1.286925266 | 2.527032276 | 0.011503093 |
| TMEM218 | 117.2683321 | -0.64198791 | 0.293773527 | -2.185315734 | 0.028865705 |
| MIA2 | 357.1898711 | -0.343383779 | 0.173919607 | -1.97438222 | 0.048338298 |
| ATPSCKMT | 54.56948909 | -0.908595458 | 0.358800196 | -2.532315948 | 0.011331184 |
| PIP4K2A | 150.3170553 | -0.85086142 | 0.353691763 | -2.405658004 | 0.016143369 |
| SCLT1 | 888.0168574 | 0.314499462 | 0.14303739 | 2.198722043 | 0.027897693 |
| VTI1A | 265.60099 | 0.36002242 | 0.174303586 | 2.065490613 | 0.038876603 |
| NR3C2 | 21.85879364 | 0.987083151 | 0.449644402 | 2.195252841 | 0.028145466 |
| ASAP2 | 50.03465116 | 0.655233941 | 0.331156732 | 1.978621833 | 0.0478586 |
| FLI1 | 31.94816447 | 1.470138996 | 0.461828712 | 3.183299258 | 0.00145607 |
| SETBP1 | 32.59930374 | -0.586735072 | 0.273742516 | -2.143383067 | 0.032082352 |
| PDK1 | 903.234773 | -0.386535964 | 0.14695796 | -2.63024857 | 0.008532246 |
| CWF19L2 | 188.0154314 | -0.549146773 | 0.266759369 | -2.058584764 | 0.039534034 |
| PLEKHH2 | 25.14600375 | -0.856723536 | 0.31192971 | -2.746527533 | 0.006022983 |
| SPARCL1 | 18.94778072 | -0.880300002 | 0.388105324 | -2.268198727 | 0.023317097 |
| FARP1 | 170.0007656 | -0.4191667 | 0.202672769 | -2.068194467 | 0.03862174 |
| HHEX | 11.44309463 | -1.755042623 | 0.586672414 | -2.991520619 | 0.002775918 |
| MR1 | 18.45363503 | -0.892380199 | 0.40201424 | -2.219772608 | 0.026434207 |
| CENPH | 758.9957614 | 0.400396375 | 0.160915605 | 2.488238317 | 0.012837767 |
| TMEM87B | 190.5494149 | 0.621254418 | 0.298064637 | 2.084294278 | 0.037133408 |
| SCN3A | 54.99758709 | -0.766843424 | 0.350958268 | -2.184998883 | 0.028888928 |
| NMRAL1 | 62.0512882 | -2.014476478 | 0.884925865 | -2.276435302 | 0.022819969 |
| RMND5A | 1710.272101 | 0.273627063 | 0.106183685 | 2.576921906 | 0.009968447 |
| CEBPG | 1447.267813 | 0.226344785 | 0.114821316 | 1.971278437 | 0.048692036 |
| ZNF599 | 32.7858856 | -0.715580898 | 0.355640447 | -2.012090871 | 0.044210359 |
| C16orf74 | 40.97391995 | -1.265752262 | 0.618885731 | -2.045211576 | 0.040834004 |
| CYP4Z2P | 3.283316328 | 2.148883217 | 0.999776778 | 2.149363003 | 0.031605636 |
| C10orf90 | 37.82146296 | -0.751621441 | 0.370762336 | -2.027232455 | 0.042638646 |
| CNTNAP3B | 19.86540135 | 1.096139771 | 0.444291171 | 2.467165323 | 0.013618746 |
| RAB6B | 21.18542411 | 0.806032426 | 0.355009177 | 2.270455184 | 0.02317998 |
| SEPTIN14 | 3.425159844 | 2.099723685 | 0.910968548 | 2.304935434 | 0.02117019 |
| PTPRN2 | 3.186500325 | 1.851197917 | 0.899024881 | 2.059117558 | 0.039482978 |
| RHOC | 522.6006736 | -2.115943419 | 0.838920894 | -2.52222043 | 0.011661661 |
| PDZD9 | 6.41309105 | 1.788346707 | 0.658344841 | 2.716428527 | 0.006599044 |
| MICU3 | 152.5926996 | -0.668644131 | 0.310909768 | -2.150605092 | 0.031507383 |
| USP16 | 2324.323949 | 0.412363114 | 0.137289937 | 3.003593143 | 0.002668119 |
| SUPV3L1 | 858.3350613 | 0.25392985 | 0.115303903 | 2.202265864 | 0.027646534 |
| UBE2L6 | 103.2027334 | -1.223980502 | 0.566770636 | -2.159569365 | 0.030806022 |
| MED19 | 50.08044383 | -1.421322312 | 0.494379873 | -2.874959905 | 0.004040788 |
| FRRS1 | 41.13905774 | -0.684433417 | 0.278847257 | -2.454510127 | 0.014107669 |
| ADGRG4 | 4.904248664 | -1.846157691 | 0.799137421 | -2.310188015 | 0.020877747 |
| ZFYVE9 | 452.4879661 | 0.323686804 | 0.145396249 | 2.226239022 | 0.025998175 |
| TMED6 | 24.82113718 | 1.021016126 | 0.480300577 | 2.125785757 | 0.033521102 |
| DHRS4 | 60.4661543 | -1.138297124 | 0.561579377 | -2.026956777 | 0.042666833 |
| CLEC18C | 2.977526255 | 3.417356091 | 1.130388718 | 3.023168967 | 0.002501425 |
| KIT | 63.41399377 | -0.900782143 | 0.350202902 | -2.572172122 | 0.010106263 |
| C2CD2 | 215.2972383 | 0.359211077 | 0.182909164 | 1.963876868 | 0.049544373 |
| PSMG3 | 157.75357 | -0.989867069 | 0.457663679 | -2.162870062 | 0.030551175 |
| DPYSL5 | 27.33508694 | -1.121814335 | 0.561824585 | -1.996734149 | 0.04585407 |
| H2BC5 | 27.54645782 | 0.797817013 | 0.404967865 | 1.970074867 | 0.048829791 |
| H4C8 | 117.157775 | -0.925077317 | 0.217776439 | -4.247830117 | 2.16E-05 |
| TSPAN33 | 3.899447208 | -2.709060477 | 1.084068842 | -2.498974579 | 0.012455325 |
| ZSCAN12 | 95.66579185 | 0.5478955 | 0.195128404 | 2.807871584 | 0.004987011 |
| TAGLN2 | 1700.032562 | -1.275419449 | 0.633470914 | -2.013382811 | 0.044074372 |
| DUSP23 | 101.6438954 | -3.33705328 | 0.987513447 | -3.379248446 | 0.000726843 |
| CDA | 136.9264265 | -0.909752575 | 0.420956039 | -2.161158154 | 0.030683125 |
| FBXW5 | 23.22182 | -2.013866511 | 0.84582304 | -2.380954901 | 0.017267826 |
| CCDC24 | 14.7455841 | -2.968760849 | 0.951268266 | -3.12084504 | 0.001803329 |
| CBR1 | 377.7521426 | -0.818518991 | 0.400811001 | -2.042157002 | 0.04113596 |
| ATP13A2 | 9.737863719 | -1.805053644 | 0.811611126 | -2.224037581 | 0.026145916 |
| PSMB4 | 265.7890516 | -0.641811907 | 0.316502452 | -2.027826021 | 0.042578008 |
| HK2 | 317.2410533 | 0.555450927 | 0.218597399 | 2.540976833 | 0.011054324 |
| CHCHD6 | 68.65508544 | -1.066628005 | 0.52332464 | -2.038176543 | 0.04153228 |
| ZYX | 24.43735186 | -3.025974078 | 1.294941659 | -2.336764793 | 0.019451417 |
| ZNF235 | 39.00960554 | 0.628459508 | 0.268631138 | 2.339488687 | 0.019310156 |
| GNE | 76.27995385 | -0.706849822 | 0.266672107 | -2.650632756 | 0.008034115 |
| IQCC | 10.94772972 | -1.101821849 | 0.542868136 | -2.029630725 | 0.042394089 |
| TMEM234 | 30.06827938 | -1.056365605 | 0.50872801 | -2.076484063 | 0.037849204 |
| UBE2J2 | 102.3850717 | -1.915985153 | 0.768957032 | -2.49166738 | 0.012714504 |
| PDE9A | 43.78955567 | 0.711199245 | 0.350560608 | 2.028748319 | 0.042483932 |
| G6PD | 34.71686746 | -1.409579674 | 0.687284143 | -2.050941649 | 0.04027263 |
| AIRE | 5.423240125 | 1.348819675 | 0.672169665 | 2.006665496 | 0.044785292 |
| ZNF66 | 237.4256572 | -0.2904131 | 0.143289072 | -2.026763776 | 0.042686577 |
| SLX9 | 88.04677692 | -1.949918883 | 0.764640574 | -2.550111714 | 0.010768841 |
| C19orf47 | 6.200485411 | -1.922831217 | 0.90019879 | -2.136007334 | 0.032678816 |
| ST6GALNAC6 | 3.377952425 | 2.022445915 | 0.984859383 | 2.053537743 | 0.040020453 |
| SHKBP1 | 64.29947147 | -2.162204375 | 0.89293848 | -2.421448312 | 0.015458799 |
| ZER1 | 24.71747143 | -1.795477032 | 0.738260135 | -2.432038448 | 0.015014113 |
| ZDHHC12 | 22.51556957 | -1.58932095 | 0.698894218 | -2.27405079 | 0.022962933 |
| JAML | 11.16820011 | 1.298487201 | 0.45543372 | 2.851100266 | 0.004356823 |
| NEK8 | 3.000722499 | -2.326571213 | 1.001883411 | -2.322197561 | 0.0202223 |
| CD3G | 3.004093603 | -2.461260671 | 0.942604172 | -2.611128555 | 0.009024396 |
| FLAD1 | 70.9200301 | -0.941177658 | 0.435665737 | -2.160320581 | 0.030747861 |
| VPS11 | 69.84951339 | -0.553588653 | 0.279417391 | -1.981224755 | 0.047566074 |
| PMF1 | 96.6887039 | -2.065984851 | 0.798806501 | -2.586339555 | 0.009700132 |
| CCDC12 | 211.9325784 | -0.955717244 | 0.354670838 | -2.694659782 | 0.007046053 |
| UBQLN4 | 49.37303248 | 0.792830032 | 0.363648862 | 2.180207655 | 0.029242072 |
| PPP1R35 | 5.402933828 | -2.132605728 | 0.95909965 | -2.223549689 | 0.026178757 |
| CPSF4 | 13.88431766 | -1.457678354 | 0.601459766 | -2.423567521 | 0.015368896 |
| LY6E | 12.74358252 | -1.719646953 | 0.74549347 | -2.306723027 | 0.021070266 |
| VPS28 | 148.8845028 | -1.660346258 | 0.77918537 | -2.130874528 | 0.03309948 |
| RECQL4 | 3.70017241 | -2.508677913 | 0.971455324 | -2.58239144 | 0.009811823 |
| LRRC14 | 13.5192377 | 1.289342922 | 0.424267757 | 3.038983994 | 0.002373775 |
| MRNIP | 23.84653216 | -1.669528842 | 0.564333362 | -2.958408902 | 0.003092316 |
| SQSTM1 | 156.3052677 | -1.517678319 | 0.77198921 | -1.965932035 | 0.049306462 |
| RPL8 | 150.5463584 | -1.243727204 | 0.549478932 | -2.263466588 | 0.023606942 |
| AP2M1 | 553.6709456 | -2.367833358 | 0.839378179 | -2.820937471 | 0.004788354 |
| U2AF1L4 | 7.802777977 | -2.086797849 | 0.881187475 | -2.368165581 | 0.017876533 |
| THAP8 | 6.728615508 | -2.973514685 | 1.240162751 | -2.397681015 | 0.016499226 |
| IP6K3 | 3.512343672 | -3.216706806 | 1.42014907 | -2.2650487 | 0.023509691 |
| SNRNP25 | 91.7083737 | -0.750116157 | 0.367865341 | -2.039105277 | 0.041439522 |
| TEDC2 | 24.49275206 | -1.862703756 | 0.546064499 | -3.411142383 | 0.000646913 |
| FLYWCH2 | 101.1760917 | -2.506469289 | 0.878866356 | -2.851934509 | 0.004345405 |
| TAF6L | 4.988501018 | 2.522788758 | 1.086339068 | 2.322284849 | 0.020217603 |
| SLC25A45 | 14.35633544 | -1.280253671 | 0.581699883 | -2.200883495 | 0.027744273 |
| RPL29 | 823.809133 | -2.430533906 | 0.78066886 | -3.113399332 | 0.001849456 |
| FGF19 | 5.813890255 | 2.797774754 | 1.166034913 | 2.399391924 | 0.016422327 |
| DHRS3 | 8.196916434 | -2.080016521 | 0.924203945 | -2.250603378 | 0.024410669 |
| WNT4 | 3.789422667 | 2.062461882 | 0.948932028 | 2.173455864 | 0.029746024 |
| ADGRL4 | 6.424605269 | 1.726508805 | 0.771095349 | 2.239034131 | 0.025153695 |
| AKNAD1 | 15.32063019 | 0.884481606 | 0.415922605 | 2.126553344 | 0.033457213 |
| GBP2 | 5.653045337 | -1.691518278 | 0.723018857 | -2.339521662 | 0.019308452 |
| ZNF496 | 53.83183603 | 0.676688702 | 0.261079598 | 2.591886565 | 0.009545124 |
| KCNJ9 | 3.639585771 | 1.81326922 | 0.877982504 | 2.065268057 | 0.038897644 |
| NCSTN | 70.09054267 | -0.869196261 | 0.423001299 | -2.054831185 | 0.039895313 |
| KLHDC9 | 26.01176185 | -1.316029946 | 0.635296392 | -2.071521201 | 0.038310115 |
| DENND2D | 10.99665213 | 1.221119928 | 0.554084626 | 2.203850948 | 0.027534828 |
| CNST | 208.7653713 | 0.360244216 | 0.164507461 | 2.189835124 | 0.028536196 |
| IL24 | 4.021640445 | -2.019995703 | 0.993916088 | -2.032360404 | 0.042117184 |
| MRPL55 | 37.98886174 | -1.3522278 | 0.573869667 | -2.356332591 | 0.018456385 |
| LINC01931 | 7.48270724 | 1.426959813 | 0.632510095 | 2.256026938 | 0.024068944 |
| NEUROD1 | 5.297229919 | 1.605560932 | 0.737744018 | 2.176311693 | 0.029531962 |
| NEURL3 | 3.658542245 | -2.216483708 | 0.945292281 | -2.34476019 | 0.019039321 |
| MSX1 | 52.21095803 | 0.741815588 | 0.30626733 | 2.422117917 | 0.015430343 |
| VPS72 | 37.41923641 | -0.91916194 | 0.422228629 | -2.176929456 | 0.029485831 |
| S100A11 | 10298.89021 | -1.036022995 | 0.351439625 | -2.947940192 | 0.003198989 |
| KBTBD8 | 58.55496768 | -0.769504277 | 0.280522154 | -2.743114105 | 0.006085953 |
| NAXE | 350.8742025 | -1.495887723 | 0.511522392 | -2.924383658 | 0.003451391 |
| RNF25 | 9.419170583 | -2.570743271 | 0.909990314 | -2.825022675 | 0.004727727 |
| CD200R1 | 9.51087634 | -1.369268124 | 0.524482333 | -2.610703996 | 0.009035606 |
| HESX1 | 17.41275475 | -1.02103835 | 0.466856132 | -2.187051384 | 0.028738773 |
| DTX3L | 101.0368777 | -0.433658513 | 0.208241254 | -2.082481282 | 0.037298531 |
| GRIK3 | 8.081254395 | 1.232269846 | 0.603850633 | 2.040686517 | 0.041281996 |
| ZNF691 | 28.75089312 | -1.366482479 | 0.549442777 | -2.487033295 | 0.012881334 |
| SPRY1 | 43.67069991 | -0.815556632 | 0.3573221 | -2.282413072 | 0.022464964 |
| TEX264 | 115.8162397 | -1.305951872 | 0.542593886 | -2.406868019 | 0.016089983 |
| DUSP7 | 70.91802508 | -1.116751875 | 0.531303141 | -2.101910921 | 0.03556108 |
| HPGD | 27.72251466 | 1.442350941 | 0.432887775 | 3.331928098 | 0.000862465 |
| CREBRF | 498.1693744 | 0.469522088 | 0.180865673 | 2.595971256 | 0.009432396 |
| PI16 | 7.042802963 | -2.216139439 | 0.85465686 | -2.593016618 | 0.009513817 |
| SLU7 | 1063.773036 | 0.263660506 | 0.120203931 | 2.193443289 | 0.028275456 |
| DEFB1 | 30.01485744 | 1.751673686 | 0.460355652 | 3.805044382 | 0.000141779 |
| TMEM74 | 36.05959234 | 0.852381493 | 0.300535489 | 2.836209115 | 0.004565254 |
| CDK5 | 19.00872605 | -1.786479626 | 0.684765433 | -2.608892828 | 0.009083569 |
| FASTK | 19.13617225 | -2.055548013 | 0.752402265 | -2.731980098 | 0.006295494 |
| TP53INP1 | 19.15135047 | -1.138969193 | 0.567144831 | -2.008251035 | 0.044616622 |
| RPP25L | 70.21010022 | -1.901196502 | 0.594068602 | -3.200297904 | 0.001372856 |
| SYK | 14.12006062 | -1.134876578 | 0.533407257 | -2.127598683 | 0.033370371 |
| CYBB | 6.526923128 | 1.471291855 | 0.716731192 | 2.052780556 | 0.040093865 |
| CARD19 | 27.02439721 | -1.89855538 | 0.665644989 | -2.85220412 | 0.004341721 |
| STOML2 | 1295.222085 | -1.153546456 | 0.494882015 | -2.330952471 | 0.019755866 |
| PGM2L1 | 970.358082 | 0.279336467 | 0.135167875 | 2.066589183 | 0.038772881 |
| EML5 | 23.67732976 | -0.991403524 | 0.401976062 | -2.466324782 | 0.01365075 |
| TMEM63C | 6.482575605 | 1.402381519 | 0.535070114 | 2.620930384 | 0.008769017 |
| NOXRED1 | 5.596361116 | -1.739526038 | 0.675646526 | -2.574609611 | 0.010035328 |
| BEND7 | 256.8416078 | 0.48437422 | 0.215031044 | 2.252578095 | 0.024285764 |
| COMTD1 | 8.672865612 | -1.9291025 | 0.702185135 | -2.74728473 | 0.006009094 |
| NSMF | 11.21944711 | -1.460260677 | 0.625458922 | -2.33470277 | 0.019558953 |
| TC2N | 24.90734633 | 0.780221213 | 0.391621009 | 1.992286409 | 0.046339642 |
| C11orf65 | 15.78670698 | -0.833836663 | 0.424002582 | -1.966583925 | 0.049231198 |
| CYB5A | 93.65149944 | 0.383585596 | 0.184297522 | 2.081338871 | 0.0374029 |
| USP54 | 49.12459416 | -0.808045055 | 0.343805355 | -2.350298047 | 0.018758384 |
| MOGAT2 | 4.456352088 | 1.715633121 | 0.864045757 | 1.985581326 | 0.047079832 |
| TUB | 376.2578896 | -0.632032149 | 0.288000197 | -2.194554573 | 0.028195565 |
| SEC11C | 646.7483347 | 0.509295076 | 0.164514439 | 3.095746972 | 0.001963178 |
| HSP90B1 | 31173.1111 | 0.392105951 | 0.185021368 | 2.119246848 | 0.034069611 |
| MMP10 | 593.0045897 | 0.32875329 | 0.115025631 | 2.858087255 | 0.004262032 |
| SLFN5 | 589.2183701 | 0.264740189 | 0.129963285 | 2.037038293 | 0.041646205 |
| ANPEP | 69.55340264 | -1.070668924 | 0.526745619 | -2.032610969 | 0.042091842 |
| PLK1 | 12.02565186 | -2.135226056 | 0.740007029 | -2.885413208 | 0.003909002 |
| PHB1 | 456.7281616 | -1.022534853 | 0.442340758 | -2.311645118 | 0.020797248 |
| SLC27A4 | 47.78621109 | -1.569493314 | 0.698150726 | -2.248072307 | 0.02457158 |
| ANKRD40CL | 4.17546192 | -1.932073223 | 0.928981193 | -2.079776468 | 0.037546041 |
| SP2 | 8.366877205 | -1.670523261 | 0.655817427 | -2.547238291 | 0.010857926 |
| RAB8A | 179.7437109 | -1.067723601 | 0.506085158 | -2.109770628 | 0.034878118 |
| GPX4 | 1090.145546 | -3.008009271 | 0.942848093 | -3.190343487 | 0.001421038 |
| YIF1B | 35.32334273 | -2.214677046 | 0.704682872 | -3.142799594 | 0.001673404 |
| EEF2 | 237.4862455 | -1.221152678 | 0.562305435 | -2.171689266 | 0.029879109 |
| MFSD3 | 3.302777263 | -2.492889901 | 1.220570948 | -2.042396557 | 0.041112211 |
| C19orf48 | 32.73008241 | -1.505931419 | 0.733965952 | -2.051772857 | 0.040191742 |
| KLK11 | 5.196921384 | 2.392388328 | 0.801369166 | 2.985376066 | 0.002832299 |
| OTUB1 | 244.4617764 | -1.883542823 | 0.553556434 | -3.402621134 | 0.000667428 |
| CD320 | 63.26326604 | -3.251698692 | 1.072943611 | -3.030633352 | 0.002440414 |
| IGFBP6 | 328.0697652 | -2.629146766 | 0.828184638 | -3.174590116 | 0.001500483 |
| CDK2AP2 | 633.3268756 | -2.506326205 | 0.946370924 | -2.648355038 | 0.008088453 |
| NUDT8 | 8.613758942 | -3.914247886 | 1.11776346 | -3.501857079 | 0.000462027 |
| PRDX2 | 72.48728732 | -2.371169687 | 0.890649171 | -2.662293712 | 0.007761012 |
| CD300A | 9.618046742 | 1.312238648 | 0.625756897 | 2.097042246 | 0.035989836 |
| TK1 | 296.116991 | -1.472736979 | 0.683860111 | -2.153564677 | 0.031274327 |
| GHDC | 11.77759098 | -1.803234894 | 0.754398779 | -2.390294024 | 0.016834889 |
| MLST8 | 21.7926922 | -1.711168965 | 0.828079448 | -2.066430906 | 0.03878781 |
| ECI1 | 33.03087408 | -0.949278503 | 0.474932637 | -1.998764516 | 0.045633839 |
| FTH1 | 8203.966345 | -3.120779645 | 1.051524892 | -2.967860931 | 0.0029988 |
| COPS6 | 192.7256382 | -1.264036123 | 0.47107694 | -2.683290171 | 0.007290171 |
| NUDT16L1 | 7.08188819 | -1.456692374 | 0.740873409 | -1.966182557 | 0.049277527 |
| HOOK3 | 1122.402674 | 0.298995512 | 0.146343514 | 2.043107372 | 0.04104181 |
| TTC39C | 390.8131073 | -0.894296468 | 0.436121379 | -2.050567827 | 0.040309052 |
| H1-4 | 43.54675842 | -1.617077857 | 0.615558518 | -2.627009145 | 0.008613902 |
| MPLKIP | 448.2411038 | -0.514818382 | 0.186371953 | -2.762316834 | 0.005739275 |
| MFSD2A | 8.432899089 | 1.218051143 | 0.519936712 | 2.342691167 | 0.019145223 |
| DTYMK | 950.555887 | -1.606073438 | 0.673237082 | -2.385598598 | 0.017051351 |
| ATG4B | 148.6836075 | -0.900470866 | 0.440042998 | -2.046324726 | 0.040724433 |
| BMP1 | 17.69380803 | -1.145033866 | 0.553415849 | -2.069029767 | 0.038543293 |
| MTCL1 | 58.26362758 | -0.486118686 | 0.239749568 | -2.027610271 | 0.04260004 |
| SNRNP48 | 519.0879455 | 0.544624052 | 0.149888467 | 3.633528728 | 0.000279571 |
| TMEM223 | 280.9743211 | -0.818628727 | 0.346022382 | -2.365825941 | 0.0179899 |
| PKIG | 77.06507225 | -1.371101311 | 0.586852366 | -2.336364972 | 0.019472228 |
| ZBTB5 | 156.7806942 | 0.71363201 | 0.248372323 | 2.873234836 | 0.00406292 |
| C2orf68 | 108.2658524 | 0.677148322 | 0.340330099 | 1.989680973 | 0.04662609 |
| BTNL3 | 3.444467822 | 4.982106739 | 1.208593618 | 4.122234857 | 3.75E-05 |
| LETM1 | 310.4902795 | -1.159133784 | 0.549942376 | -2.107736802 | 0.035053762 |
| DHRSX | 163.3129938 | -1.33736875 | 0.492885484 | -2.713345783 | 0.006660756 |
| XPO6 | 92.16374667 | -0.944329392 | 0.40013861 | -2.360005679 | 0.018274655 |
| RGS14 | 3.116103851 | -2.757704445 | 1.204559412 | -2.289388483 | 0.022056791 |
| TBC1D10B | 19.30547091 | -2.640492906 | 0.576454727 | -4.580572909 | 4.64E-06 |
| LMAN2 | 334.6256017 | -1.026568748 | 0.498277077 | -2.060236754 | 0.039375913 |
| PRELID1 | 7.824932907 | -3.364728134 | 1.274508452 | -2.64002026 | 0.008290107 |
| UMOD | 4.48846004 | 1.671752319 | 0.781978926 | 2.137848302 | 0.032529057 |
| KCNK9 | 8.197795417 | 1.72723016 | 0.676076203 | 2.554786212 | 0.010625304 |
| GPR183 | 21.79447039 | -1.074980373 | 0.383356057 | -2.804130402 | 0.00504525 |
| CENPX | 191.9131985 | -1.040679717 | 0.393676449 | -2.643489903 | 0.008205621 |
| AGPAT2 | 30.26110745 | -1.560895627 | 0.781206288 | -1.998058196 | 0.045710351 |
| GPS1 | 147.6451633 | -1.57513942 | 0.74593381 | -2.111634303 | 0.034717828 |
| DCXR | 117.7099112 | -1.916025513 | 0.757217322 | -2.530350875 | 0.011394851 |
| NRG4 | 6.699053142 | -1.639925289 | 0.759215152 | -2.160027083 | 0.030770573 |
| MUC17 | 3.937036375 | -2.488017887 | 1.071319206 | -2.322387085 | 0.020212102 |
| OTUD3 | 133.3746104 | -0.542242237 | 0.215722335 | -2.513611944 | 0.011950185 |
| TMEM42 | 35.33075015 | -1.491308058 | 0.601869219 | -2.477794195 | 0.013219737 |
| TIGD4 | 7.3192359 | -1.694642242 | 0.780890402 | -2.170140955 | 0.029996169 |
| TRAPPC1 | 34.95390391 | -1.955330793 | 0.744610896 | -2.625976605 | 0.008640076 |
| CHRNB1 | 19.13138428 | 0.887433016 | 0.410198236 | 2.163424752 | 0.030508526 |
| UBB | 355.287416 | -0.708530648 | 0.339733735 | -2.085546929 | 0.037019683 |
| MGMT | 255.9948605 | -1.080518262 | 0.431796079 | -2.502380901 | 0.012336112 |
| HSPA4 | 1726.705641 | 0.303109578 | 0.15214588 | 1.992229939 | 0.046345835 |
| COMMD5 | 61.48526562 | -1.669323164 | 0.636481234 | -2.622737445 | 0.008722646 |
| DPY19L2P2 | 14.32552983 | -1.829915072 | 0.770546052 | -2.374828952 | 0.017557084 |
| HOXB9 | 20.7905071 | -1.265858543 | 0.616718374 | -2.052571474 | 0.040114156 |
| OR10A2 | 6.240220259 | -1.903360887 | 0.827740618 | -2.299465372 | 0.021478528 |
| TSEN34 | 198.1512388 | -0.717296171 | 0.358803447 | -1.999134001 | 0.045593857 |
| CAVIN3 | 65.24860382 | -2.438652775 | 0.84204401 | -2.896110827 | 0.003778189 |
| PDGFD | 27.52492607 | -1.241259335 | 0.49660966 | -2.499466751 | 0.012438037 |
| C11orf24 | 160.5873643 | -0.869276208 | 0.352142459 | -2.468535633 | 0.013566714 |
| TRMT61B | 46.49407801 | -0.912906997 | 0.357288427 | -2.555098146 | 0.010615786 |
| ATP6V0E2 | 4.226426435 | -1.795130991 | 0.866862115 | -2.070837981 | 0.03837394 |
| BBLN | 71.45246701 | -4.060076065 | 1.12048893 | -3.623486102 | 0.000290659 |
| GRIK1 | 47.19416526 | -0.66812963 | 0.324022958 | -2.06198238 | 0.039209414 |
| SCAND1 | 263.9706309 | -3.246880981 | 0.933495984 | -3.47819491 | 0.000504803 |
| XAGE5 | 3.580443128 | -3.394136425 | 1.444114976 | -2.35032285 | 0.018757134 |
| RGS19 | 17.86840499 | -1.941289692 | 0.894729381 | -2.169694807 | 0.030029974 |
| ADRA1D | 2.955705699 | -2.140842916 | 1.062541828 | -2.014831662 | 0.04392229 |
| MOB3A | 23.2763779 | -1.849965165 | 0.771710688 | -2.397226311 | 0.016519717 |
| CALB2 | 8.796011829 | -1.115733621 | 0.541121115 | -2.061892598 | 0.039217962 |
| CLEC7A | 2.972871071 | -2.444042493 | 1.068087689 | -2.288241422 | 0.022123466 |
| POP7 | 125.9027497 | -1.758117241 | 0.728352943 | -2.413825958 | 0.015786001 |
| GNB2 | 96.46446827 | -1.665599198 | 0.773777987 | -2.152554383 | 0.031353717 |
| FIBP | 469.3632467 | -0.990680583 | 0.478032748 | -2.072411537 | 0.038227078 |
| PPP1CA | 178.9881172 | -3.23775282 | 1.171826736 | -2.762996201 | 0.005727343 |
| TMEM134 | 63.06152 | -2.353149976 | 0.823058409 | -2.859031571 | 0.004249365 |
| CFL1 | 5575.840951 | -5.057956156 | 1.131022213 | -4.472021946 | 7.75E-06 |
| LVRN | 121.1391181 | 0.755967777 | 0.242912392 | 3.112100506 | 0.001857612 |
| OXSR1 | 182.9091434 | 0.458057699 | 0.183052074 | 2.502335473 | 0.012337696 |
| GXYLT2 | 170.5488268 | 0.504348537 | 0.198661938 | 2.538727562 | 0.011125642 |
| RHOD | 22.85139758 | -2.091076212 | 0.856744468 | -2.440723332 | 0.01465788 |
| PARP15 | 7.506080549 | 1.394438584 | 0.684626095 | 2.036788539 | 0.041671237 |
| ZNF483 | 172.8094728 | -0.596153161 | 0.240547295 | -2.478319952 | 0.013200271 |
| ZNRD2 | 45.63783343 | -1.998277398 | 0.648373847 | -3.081983345 | 0.002056263 |
| NUDT4 | 771.4720784 | 0.44051689 | 0.174362307 | 2.526445639 | 0.011522322 |
| NMNAT1 | 41.83545286 | -0.959889939 | 0.353689082 | -2.713937149 | 0.006648878 |
| RCE1 | 33.85384467 | -1.387536798 | 0.643515902 | -2.156181056 | 0.03106953 |
| SUSD5 | 104.2376812 | -0.770361569 | 0.308859738 | -2.494211692 | 0.012623723 |
| PHOSPHO1 | 2.838904515 | 2.084037803 | 1.062577922 | 1.961303505 | 0.049843629 |
| RBM4B | 85.41892604 | -0.444147277 | 0.225796991 | -1.967020356 | 0.049180864 |
| UBXN2A | 868.2443827 | 0.257198562 | 0.112014854 | 2.296111217 | 0.021669523 |
| C16orf91 | 347.1378459 | -1.008543313 | 0.390375677 | -2.58351986 | 0.009779784 |
| PHLDA3 | 14.02429627 | -1.698568982 | 0.721119864 | -2.355459983 | 0.018499789 |
| SNHG11 | 54.08085468 | -1.304537441 | 0.487278638 | -2.677189884 | 0.007424255 |
| ABRA | 14.43265558 | -1.71691628 | 0.644292873 | -2.66480719 | 0.007703247 |
| IL20RB | 21.42340029 | 1.012987722 | 0.436518364 | 2.320607344 | 0.020308045 |
| YIF1A | 33.33146416 | -2.544779477 | 0.791347687 | -3.215753982 | 0.001301023 |
| MICOS13 | 10.20383927 | -1.721766228 | 0.703936738 | -2.445910456 | 0.014448688 |
| P2RY14 | 7.914957533 | -3.603233984 | 0.905159196 | -3.98077377 | 6.87E-05 |
| ZDHHC14 | 36.6243753 | 0.777693345 | 0.361381339 | 2.152001949 | 0.031397201 |
| CTDSP2 | 475.8790937 | 0.330883107 | 0.144244009 | 2.293912306 | 0.021795536 |
| ARHGAP1 | 67.28999726 | -1.064791108 | 0.469383619 | -2.268487999 | 0.02329948 |
| MED16 | 27.15148269 | -1.824810619 | 0.76836287 | -2.374933367 | 0.017552118 |
| CATSPER1 | 25.67271408 | -0.934753315 | 0.44249284 | -2.11247105 | 0.034646066 |
| DRAP1 | 178.9848904 | -4.152436005 | 1.08938494 | -3.811725178 | 0.000138 |
| C11orf68 | 38.17923631 | -1.309810102 | 0.605863518 | -2.161889705 | 0.030626679 |
| PAAF1 | 36.36754371 | -0.942496776 | 0.448047275 | -2.103565466 | 0.035416369 |
| CCDC85B | 44.21086111 | -2.073789727 | 0.874035218 | -2.372661518 | 0.01766044 |
| AURKAIP1 | 113.5047884 | -2.414849027 | 0.857891999 | -2.814863678 | 0.004879793 |
| SFN | 8.800018711 | -1.87647017 | 0.899563287 | -2.085979048 | 0.036980521 |
| CCDC168 | 58.63268966 | 0.761155923 | 0.360939882 | 2.108816348 | 0.034960437 |
| TMEM270 | 2.853427409 | 3.035379037 | 1.486750772 | 2.041619277 | 0.041189312 |
| TSPEAR | 4.449376463 | 1.738535572 | 0.864276496 | 2.011550215 | 0.044267372 |
| LINC02880 | 3.820638392 | -2.119357367 | 1.051591236 | -2.015381352 | 0.043864707 |
| NUPR1 | 42.50738017 | -1.772959694 | 0.877214415 | -2.02112467 | 0.043266861 |
| RTTN | 382.0620962 | -0.410676176 | 0.171793957 | -2.390515839 | 0.016824723 |
| KCMF1 | 1696.419768 | 0.39194416 | 0.136344622 | 2.87465801 | 0.004044653 |
| SGF29 | 80.68799735 | -2.757086229 | 0.954231379 | -2.889326727 | 0.003860677 |
| OR5AN1 | 7.842598473 | 1.438053269 | 0.592760118 | 2.426029055 | 0.015265048 |
| HORMAD2 | 4.846555692 | 3.333749466 | 0.951176028 | 3.5048712 | 0.000456828 |
| BOK | 37.81803265 | -1.525445506 | 0.555650445 | -2.745333006 | 0.006044952 |
| EFCAB5 | 25.561916 | -0.797090198 | 0.359754879 | -2.215648058 | 0.026715615 |
| TRIM60 | 4.304054454 | 2.037705669 | 0.924887736 | 2.203192441 | 0.027581188 |
| TMEM80 | 48.48554081 | -1.143093522 | 0.385895911 | -2.962180963 | 0.003054682 |
| RHOG | 177.4103371 | -1.708016722 | 0.721532095 | -2.367208241 | 0.017922845 |
| MAN1B1 | 14.30595421 | -1.211546676 | 0.593166922 | -2.042505458 | 0.041101419 |
| KCNA3 | 7.685811835 | 1.402944373 | 0.683884199 | 2.051435572 | 0.040224548 |
| KCNA2 | 18.52708252 | 1.316273429 | 0.534768503 | 2.461389222 | 0.013840013 |
| ZBED2 | 6.455521784 | -1.260056546 | 0.629437531 | -2.001877047 | 0.045297957 |
| NHLH2 | 4.049621976 | -1.966497523 | 0.821567549 | -2.393592014 | 0.016684296 |
| PNPLA2 | 11.79000676 | -2.929331154 | 0.878744454 | -3.333541557 | 0.000857479 |
| SUMO4 | 22.20683267 | 0.921568444 | 0.417327361 | 2.208262699 | 0.027225966 |
| CD151 | 56.42827513 | -1.717948337 | 0.554665072 | -3.097271534 | 0.001953109 |
| CHID1 | 43.45413198 | -1.460909718 | 0.69802685 | -2.092913358 | 0.03635689 |
| ZNF620 | 24.36219306 | 1.37674199 | 0.611578767 | 2.251127842 | 0.024377441 |
| TMEM187 | 18.37598847 | -1.383328263 | 0.585901865 | -2.361023825 | 0.01822456 |
| DPY19L2 | 16.08348714 | 1.454290696 | 0.577553972 | 2.51801696 | 0.011801764 |
| NDUFAF3 | 212.8496589 | -1.004862945 | 0.424948711 | -2.364668768 | 0.018046203 |
| BOLA1 | 69.66082208 | -2.206035265 | 0.645049629 | -3.419946571 | 0.000626334 |
| PLEC | 128.5510825 | -1.93703263 | 0.819522209 | -2.363612126 | 0.018097749 |
| RNF212 | 44.56460011 | -1.004506017 | 0.31797899 | -3.159032672 | 0.001582937 |
| TMPRSS9 | 4.422311752 | -1.822976092 | 0.893140909 | -2.041084531 | 0.041242426 |
| GLDC | 21.469109 | -1.039288027 | 0.395598766 | -2.627126564 | 0.00861093 |
| KLHL11 | 54.11665992 | 0.775010253 | 0.310057964 | 2.499565704 | 0.012434564 |
| LMO7DN | 4.092722164 | -2.018898691 | 0.851701161 | -2.37043083 | 0.017767368 |
| FAM219B | 16.07882018 | -1.728336739 | 0.64004261 | -2.700346372 | 0.006926732 |
| CD300LB | 39.40043186 | -1.345653889 | 0.527509992 | -2.550954312 | 0.010742841 |
| EXOSC4 | 51.94759375 | -2.145137678 | 0.664044169 | -3.230414147 | 0.00123611 |
| TRIML2 | 18.99197846 | -1.386462471 | 0.599746395 | -2.311747903 | 0.02079158 |
| CYC1 | 1151.756047 | -1.810632357 | 0.723601652 | -2.502250169 | 0.012340669 |
| GVQW3 | 65.29374271 | -0.567926674 | 0.262501522 | -2.16351764 | 0.030501388 |
| LDLRAD3 | 317.9718825 | 0.704774552 | 0.209986385 | 3.356286902 | 0.000789966 |
| RAD23A | 117.5345932 | -1.595501985 | 0.770250546 | -2.071406497 | 0.038320824 |
| GADD45GIP1 | 316.1709756 | -1.196935726 | 0.522883084 | -2.289107764 | 0.022073092 |
| SHARPIN | 46.65232158 | -2.340699344 | 0.715628164 | -3.270831782 | 0.001072317 |
| GCC1 | 251.1752635 | 0.571363188 | 0.167921828 | 3.402554592 | 0.00066759 |
| LINC00305 | 3.670543292 | 2.207179495 | 0.703138834 | 3.139037966 | 0.001695035 |
| SERTAD2 | 1038.40916 | 0.263904908 | 0.123662857 | 2.134067698 | 0.03283724 |
| TIGD5 | 5.386950775 | -1.749366137 | 0.723158209 | -2.419064204 | 0.015560493 |
| FIZ1 | 11.15061436 | -1.173469786 | 0.54568571 | -2.150449909 | 0.031519644 |
| RCC1 | 25.34982206 | -1.470276407 | 0.427697034 | -3.43765865 | 0.000586767 |
| PRKRA | 253.955458 | -0.536639458 | 0.141321072 | -3.797306743 | 0.000146277 |
| OAZ2 | 76.46316015 | -1.120742617 | 0.426900678 | -2.625300625 | 0.008657249 |
| MEIOC | 97.67486552 | -0.627621364 | 0.200234361 | -3.134433878 | 0.001721861 |
| FZD2 | 89.70938014 | 0.651930792 | 0.2523219 | 2.58372655 | 0.009773925 |
| TIGD2 | 128.4196697 | 0.558850037 | 0.263103445 | 2.12406963 | 0.033664322 |
| H2AC6 | 306.9566534 | -1.010013552 | 0.221947754 | -4.550681559 | 5.35E-06 |
| H2BC4 | 13.08695892 | -1.53443746 | 0.524234603 | -2.9270053 | 0.00342243 |
| LRRC57 | 85.96146294 | -0.468232757 | 0.231261491 | -2.024689696 | 0.042899237 |
| MRPL14 | 691.9484741 | -0.556712537 | 0.270028638 | -2.061679611 | 0.039238249 |
| C1orf105 | 4.642938995 | 3.188274713 | 1.124944142 | 2.834162688 | 0.004594593 |
| OR52N5 | 42.24041944 | -1.350751183 | 0.490257755 | -2.755185753 | 0.005865882 |
| POLR2A | 23.11093448 | -0.870983523 | 0.442334625 | -1.969060238 | 0.048946174 |
| P2RY13 | 3.182026673 | 2.523932483 | 1.198593073 | 2.105745928 | 0.035226428 |
| PHLDA2 | 181.546249 | -2.260602275 | 0.905480683 | -2.49657703 | 0.012539843 |
| CELF2-AS1 | 3.408122809 | 2.248651036 | 0.940167304 | 2.391756261 | 0.016767974 |
| LSM10 | 532.7524636 | -1.222421045 | 0.442328343 | -2.763605506 | 0.005716661 |
| COA4 | 488.4624054 | -0.630637363 | 0.318930113 | -1.977352836 | 0.048001761 |
| MRPS11 | 267.2280178 | -0.951181954 | 0.424738018 | -2.239455651 | 0.025126284 |
| IDH2 | 44.21745086 | -1.161235206 | 0.492657811 | -2.357082704 | 0.018419144 |
| ZNF716 | 25.88653927 | -2.769997954 | 0.483239519 | -5.732142853 | 9.92E-09 |
| TDRKH | 54.61087361 | 1.131342693 | 0.306313057 | 3.693419747 | 0.000221258 |
| ZNF708 | 182.6090654 | -0.649926599 | 0.203151271 | -3.199224868 | 0.001377976 |
| MRPL41 | 135.5783658 | -0.99154135 | 0.461519831 | -2.148426314 | 0.031679904 |
| LDOC1 | 70.64857735 | -1.367284078 | 0.561180051 | -2.436444553 | 0.014832443 |
| DCAF4L1 | 81.32785957 | 0.766469973 | 0.356323676 | 2.151049805 | 0.031472269 |
| HOXB4 | 6.488601952 | -1.755948674 | 0.751993358 | -2.335058755 | 0.019540352 |
| CRIP2 | 246.7024647 | -2.106350669 | 0.875560193 | -2.405717718 | 0.016140731 |
| PLCXD3 | 4.202663841 | 1.668228981 | 0.796174974 | 2.095304467 | 0.036143936 |
| LCK | 3.586608186 | 1.677465979 | 0.708379896 | 2.368031599 | 0.017883008 |
| CEP63 | 317.0455411 | -0.382108574 | 0.169975537 | -2.248020975 | 0.024574852 |
| WBP2NL | 19.4706947 | -1.095392821 | 0.416249097 | -2.631580053 | 0.008498884 |
| GABRR3 | 7.337143267 | 1.419873148 | 0.644694805 | 2.202395828 | 0.02763736 |
| RUVBL2 | 188.2500971 | -2.610259388 | 0.878924386 | -2.969833845 | 0.002979609 |
| WT1-AS | 6.491066432 | 1.315256013 | 0.512924951 | 2.564227009 | 0.010340589 |
| COL18A1-AS1 | 5.478742357 | 1.812045551 | 0.835113519 | 2.169819444 | 0.030020527 |
| FBXL7 | 6.02657232 | 2.870364821 | 0.788018792 | 3.642508087 | 0.000269994 |
| FAM167B | 4.26441523 | -3.831422534 | 1.153948464 | -3.320271792 | 0.000899298 |
| CMKLR2 | 46.07344047 | 1.108791871 | 0.344262114 | 3.220778083 | 0.001278431 |
| CBX6 | 268.8790106 | -2.292179785 | 0.813778613 | -2.816711756 | 0.004851805 |
| BTBD9 | 47.26899689 | -0.537816716 | 0.255118156 | -2.108108354 | 0.035021618 |
| NUDT14 | 25.44329544 | -2.374171858 | 0.766657972 | -3.096781023 | 0.001956343 |
| ANKRD45 | 5.579864857 | -1.714836835 | 0.804822768 | -2.130701198 | 0.033113766 |
| SCN5A | 9.229721273 | 1.099042396 | 0.538123562 | 2.042360666 | 0.041115768 |
| PRKX | 123.0191533 | -0.448619136 | 0.201393289 | -2.227577383 | 0.02590871 |
| BRD7P2 | 10.04773171 | 1.675055778 | 0.72834997 | 2.299795219 | 0.021459824 |
| KCNQ3 | 31.81148909 | -1.00984946 | 0.359151518 | -2.811764419 | 0.004927057 |
| NR2C2AP | 150.7366812 | -0.839324742 | 0.413138365 | -2.031582667 | 0.042195923 |
| TSSC4 | 25.53491132 | -1.729177678 | 0.697728166 | -2.478297082 | 0.013201118 |
| TXNRD2 | 55.98677426 | -1.063244795 | 0.506605132 | -2.09876436 | 0.035837678 |
| PDE4B | 37.0511567 | 1.056088949 | 0.345294841 | 3.05851355 | 0.00222438 |
| RALYL | 7.691130347 | 2.081983334 | 0.700356697 | 2.972747092 | 0.002951475 |
| AMER1 | 7.15647995 | 1.392745423 | 0.584522495 | 2.382706285 | 0.0171859 |
| DYNLT2 | 214.1556545 | 0.549750334 | 0.240570111 | 2.28519799 | 0.022301221 |
| JAG2 | 10.08623208 | -1.190624462 | 0.515271189 | -2.310675404 | 0.020850791 |
| SIVA1 | 157.5723367 | -1.9751863 | 0.725898404 | -2.721023063 | 0.006508022 |
| IFNE | 16.24720724 | -1.425870465 | 0.676074409 | -2.10904369 | 0.034940811 |
| SLC22A10 | 7.603895665 | 2.561757256 | 0.708556169 | 3.615461087 | 0.000299814 |
| ROBO2 | 11.77815695 | -1.560057015 | 0.739405572 | -2.109879981 | 0.034868695 |
| CIB1 | 1388.559574 | -0.961956226 | 0.375725879 | -2.560260761 | 0.010459365 |
| HSF1 | 28.98972175 | -1.351865153 | 0.64537547 | -2.094695595 | 0.036198061 |
| IFITM2 | 84.94215894 | -1.322969359 | 0.490187766 | -2.69890326 | 0.00695684 |
| RAB11B | 5.968871654 | -2.212129032 | 1.113362696 | -1.986889842 | 0.046934606 |
| KIAA0825 | 42.87255308 | -0.555951319 | 0.264335233 | -2.103205512 | 0.035447809 |
| UBALD2 | 55.15038167 | 0.665823604 | 0.315663231 | 2.109284639 | 0.03492002 |
| LINC00158 | 35.99691594 | -0.777870798 | 0.359737045 | -2.162331647 | 0.030592622 |
| FAM174B | 32.78550858 | -1.55096582 | 0.510019274 | -3.040994525 | 0.002357981 |
| ZSWIM9 | 7.495654704 | 1.423006873 | 0.639298353 | 2.225888534 | 0.026021649 |
| TMEM179B | 426.9338314 | -1.372342918 | 0.492883154 | -2.784316944 | 0.005364059 |
| PSMD13 | 845.2047201 | -1.206140555 | 0.486093353 | -2.481294069 | 0.013090633 |
| CXorf38 | 183.9794011 | 0.318787121 | 0.158292735 | 2.013908735 | 0.044019116 |
| KCNQ5 | 14.82701449 | -1.448805959 | 0.652121806 | -2.221679978 | 0.026304941 |
| NAT8L | 3.530254085 | 1.682595164 | 0.84879035 | 1.982344833 | 0.047440659 |
| CCDC190 | 5.696434533 | 1.45073123 | 0.642950554 | 2.256365162 | 0.024047772 |
| NPIPB4 | 8.589146565 | -1.41853269 | 0.595803884 | -2.380871841 | 0.01727172 |
| MATN1-AS1 | 4.038723823 | -2.365004019 | 1.1155822 | -2.119972889 | 0.034008332 |
| GSAP | 7.42282514 | 1.433434028 | 0.58899375 | 2.433699893 | 0.014945381 |
| SOWAHB | 5.219299658 | 1.424740136 | 0.68081301 | 2.092704041 | 0.036375582 |
| LINC02610 | 3.295984993 | 2.197416222 | 0.960979294 | 2.286642633 | 0.022216691 |
| KDM4D | 5.361550161 | -1.443522924 | 0.704335657 | -2.049481534 | 0.040415052 |
| PRELID2 | 116.9149107 | -0.456312722 | 0.1983627 | -2.300395802 | 0.021425806 |
| BACE1 | 44.6303569 | 0.696549807 | 0.257547908 | 2.704544613 | 0.006839808 |
| RXRA | 17.45410654 | -1.080042571 | 0.539975321 | -2.000170246 | 0.045481884 |
| GLDN | 8.883483222 | 1.87363414 | 0.735692068 | 2.546764089 | 0.010872691 |
| SPATA12 | 16.06501891 | -1.390179811 | 0.641047952 | -2.168605026 | 0.030112683 |
| SMIM29 | 21.47980466 | -2.803785203 | 0.719323215 | -3.897809979 | 9.71E-05 |
| UBE2H | 416.5931641 | 0.412527976 | 0.14246927 | 2.895557583 | 0.003784856 |
| ZNF732 | 14.21865206 | -3.766266213 | 0.826514682 | -4.556804974 | 5.19E-06 |
| HYAL3 | 23.06319891 | -1.576137233 | 0.630669424 | -2.499149592 | 0.012449175 |
| TPCN1 | 11.59015329 | -1.209134391 | 0.499056611 | -2.422840142 | 0.015399702 |
| C1QL4 | 3.260300775 | 2.877694437 | 1.228925251 | 2.341635046 | 0.019199479 |
| ZNF395 | 370.8835953 | 0.399924023 | 0.201485961 | 1.984872895 | 0.047158615 |
| KRTAP21-2 | 96.94139294 | 1.551116994 | 0.412771973 | 3.757805996 | 0.00017141 |
| HEATR4 | 14.18943859 | -1.10244918 | 0.49386707 | -2.232279186 | 0.025596517 |
| AGMO | 6.587718481 | -1.617562026 | 0.820215016 | -1.972119498 | 0.048595967 |
| TLR5 | 10.53157149 | -1.164910125 | 0.529970956 | -2.198064084 | 0.02794454 |
| ISG15 | 40.77593624 | -1.34937259 | 0.589417769 | -2.289331373 | 0.022060106 |
| ZFP69B | 25.54104069 | -0.823110916 | 0.331934307 | -2.479740416 | 0.013147806 |
| EIF4EBP1 | 720.2769809 | -3.250946987 | 0.982923829 | -3.307425146 | 0.000941579 |
| GFRAL | 3.159774145 | -3.1585235 | 1.151069935 | -2.743989226 | 0.006069753 |
| ZFTRAF1 | 28.15556274 | -1.142343254 | 0.55955796 | -2.041510149 | 0.041200146 |
| TMEM276 | 28.15556274 | -1.142343254 | 0.55955796 | -2.041510149 | 0.041200146 |
| LOC84773-CYHR1 | 28.15556274 | -1.142343254 | 0.55955796 | -2.041510149 | 0.041200146 |
| KLHL17 | 8.044372693 | -1.425615103 | 0.720915516 | -1.97750648 | 0.047984409 |
| NWD1 | 20.32619353 | -1.05584353 | 0.472948585 | -2.232470006 | 0.025583915 |
| OR2AG2 | 6.397941243 | 1.449687028 | 0.727935244 | 1.99150548 | 0.046425343 |
| AGAP4 | 14.18258195 | -1.262244201 | 0.582780661 | -2.165899258 | 0.030318886 |
| NKAIN2 | 5.53778805 | 1.621997916 | 0.723122058 | 2.243048596 | 0.024893681 |
| S100A16 | 2227.745034 | -0.682037626 | 0.330775907 | -2.061932601 | 0.039214153 |
| UROS | 336.9726506 | -1.029947625 | 0.432436595 | -2.381730955 | 0.017231482 |
| QRFP | 3.074280836 | -2.612318886 | 1.060762298 | -2.462680744 | 0.013790265 |
| NOXA1 | 4.324751203 | 1.972370845 | 0.674448359 | 2.924420852 | 0.003450978 |
| RPSAP47 | 3.029575576 | 2.387026775 | 1.172098182 | 2.036541659 | 0.041695994 |
| TMC3 | 3.879297213 | 1.748339814 | 0.885311596 | 1.974829905 | 0.048287454 |
| UTS2B | 11.39981329 | -1.270887478 | 0.58304099 | -2.179756654 | 0.029275505 |
| CLDN4 | 4.405807051 | -1.976600121 | 0.899805621 | -2.196696791 | 0.028042108 |
| C15orf61 | 19.2782845 | -0.932449029 | 0.362396554 | -2.573007435 | 0.010081904 |
| RRP7A | 116.3575272 | -1.965637399 | 0.78657593 | -2.498979849 | 0.012455139 |
| TOGARAM2 | 11.65020217 | 1.159853432 | 0.587292873 | 1.974914878 | 0.048277809 |
| NEMP2 | 174.2920401 | -0.534256107 | 0.245026103 | -2.180404866 | 0.029227464 |
| RASSF10 | 5.467106633 | 1.688935177 | 0.852621375 | 1.980873604 | 0.04760545 |
| GJB4 | 4.006725369 | 1.88875483 | 0.849578474 | 2.223167003 | 0.026204541 |
| ZNF676 | 110.0749511 | -0.694894975 | 0.220867494 | -3.146207546 | 0.001654026 |
| ADAM5 | 6.561731307 | -1.806539964 | 0.65016156 | -2.778601622 | 0.005459343 |
| TDRD7 | 151.3197538 | 0.470055897 | 0.215497379 | 2.181260396 | 0.029164162 |
| FAM217B | 192.963334 | 0.440857028 | 0.183163342 | 2.406906443 | 0.016088291 |
| CGB7 | 6.079443983 | -3.886956174 | 1.23163718 | -3.155926305 | 0.001599893 |
| SLC35F1 | 6.464125173 | -2.47032245 | 0.889427009 | -2.77743134 | 0.005479041 |
| ZNF799 | 92.65805992 | -0.706615013 | 0.322431849 | -2.19151742 | 0.028414371 |
| MME | 21.48340806 | 1.345858767 | 0.47452368 | 2.836230989 | 0.004564942 |
| TCF4 | 94.52051164 | 0.420440032 | 0.197045056 | 2.133725353 | 0.032865269 |
| ZNF700 | 254.9215903 | -0.43301558 | 0.189572164 | -2.284172792 | 0.022361378 |
| SCN8A | 58.46908527 | -0.614282178 | 0.303062152 | -2.026918152 | 0.042670784 |
| PDLIM7 | 114.9264376 | -1.292058193 | 0.571676152 | -2.260122603 | 0.023813643 |
| SERTAD1 | 60.69894931 | -2.483736308 | 0.68034362 | -3.650708607 | 0.000261518 |
| ZNF441 | 124.4856382 | 0.424790267 | 0.203865124 | 2.083682868 | 0.037189024 |
| SLC6A17 | 9.283272734 | 1.430783424 | 0.583797094 | 2.450823133 | 0.014252997 |
| H3C12 | 3.031497881 | -2.439447717 | 1.048906712 | -2.325705125 | 0.020034287 |
| DCHS2 | 18.15187327 | 0.923788652 | 0.415478232 | 2.22343454 | 0.026186513 |
| GSTK1 | 217.188156 | -0.954069081 | 0.400903834 | -2.37979535 | 0.017322255 |
| ZNF695 | 41.98444239 | -0.702131632 | 0.297409692 | -2.360822968 | 0.018234433 |
| HHLA3 | 38.34955498 | -1.96460914 | 0.646319496 | -3.039687264 | 0.002368239 |
| ZNF460 | 41.43588303 | -0.652347422 | 0.330971616 | -1.971007148 | 0.048723058 |
| CFAP43 | 37.59642541 | -0.582465751 | 0.287158593 | -2.028376529 | 0.042521834 |
| FAM118B | 105.6093688 | -0.53167658 | 0.249603195 | -2.130087233 | 0.033164412 |
| GPAA1 | 74.66701354 | -1.147283121 | 0.556067283 | -2.063209175 | 0.039092759 |
| DNM3 | 65.60317482 | -0.713122687 | 0.290425817 | -2.455438348 | 0.014071289 |
| ZNF121 | 507.6352464 | -0.251488296 | 0.11802923 | -2.130728938 | 0.03311148 |
| VPS13A | 1268.539647 | -0.420352862 | 0.209380055 | -2.007606994 | 0.044685071 |
| TMPRSS11F | 9.561745272 | 2.226603816 | 0.631247918 | 3.527304809 | 0.000419813 |
| CACNA1E | 18.19157377 | 0.781302927 | 0.385757119 | 2.025375267 | 0.042828845 |
| ZKSCAN8 | 407.6869067 | -0.50992867 | 0.253887841 | -2.008480077 | 0.044592301 |
| GET3 | 80.4557749 | -3.594699783 | 1.147478582 | -3.132694449 | 0.001732097 |
| UVRAG | 223.8557996 | 0.40323898 | 0.168487647 | 2.393285127 | 0.016698259 |
| ZNF43 | 371.6661965 | -0.273012746 | 0.134241776 | -2.033739073 | 0.04197791 |
| ZNF627 | 89.15982957 | -0.451692661 | 0.222503745 | -2.030045207 | 0.042351944 |
| ZNF789 | 63.56312252 | -0.999886153 | 0.27430031 | -3.645224293 | 0.000267159 |
| ND6 | 730.2324224 | -1.042675744 | 0.429226868 | -2.429194959 | 0.015132393 |
| CYTB | 30024.5509 | -0.755242384 | 0.306697024 | -2.462503141 | 0.013797096 |
| MSRB1 | 86.02382109 | -1.707991518 | 0.810037808 | -2.108533084 | 0.034984904 |
| COLGALT2 | 9.066968339 | 1.458161292 | 0.718824276 | 2.028536514 | 0.042505521 |
| ND2 | 11070.75764 | -0.685401224 | 0.306274251 | -2.237867607 | 0.02522969 |
| RCSD1 | 8.754792432 | -1.688110708 | 0.729353616 | -2.314529839 | 0.020638676 |
| RASSF9 | 13.52001454 | 1.494288645 | 0.542101343 | 2.756474716 | 0.005842813 |
| ND5 | 3960.045412 | -1.207063441 | 0.43620083 | -2.767219498 | 0.005653668 |
| TMEM184B | 41.71523565 | -0.919604657 | 0.451240999 | -2.037945708 | 0.041555363 |
| PAX9 | 9.158950437 | 1.094634281 | 0.555081972 | 1.972022757 | 0.048607009 |
| SUCNR1 | 4.171645682 | 3.279056015 | 1.270506691 | 2.580904169 | 0.009854193 |
| ND4 | 48802.40743 | -0.657056014 | 0.253867695 | -2.588182847 | 0.009648375 |
| ND1 | 19418.44809 | -0.754045708 | 0.293136852 | -2.572333377 | 0.010101557 |
| ATP6 | 6400.117983 | -0.746862742 | 0.215328229 | -3.46848505 | 0.000523402 |
| APRT | 59.72186012 | -1.504840216 | 0.62916925 | -2.391789198 | 0.016766469 |
| COX3 | 20078.43925 | -0.747249036 | 0.306932276 | -2.434573015 | 0.014909371 |
| SMG5 | 51.49253487 | -1.044541742 | 0.518962344 | -2.012750546 | 0.044140879 |
| LOC124904381 | 3.005526862 | -2.019173075 | 0.879620549 | -2.295504666 | 0.021704219 |
| RNU6-1188P | 6.351313255 | -1.871734072 | 0.721114265 | -2.595613708 | 0.009442215 |
| LOC124900226 | 3.269109071 | -1.969297214 | 0.922959002 | -2.133677889 | 0.032869157 |
| LOC100422622 | 2.998600813 | -3.063285232 | 1.223906928 | -2.502874329 | 0.012318928 |
| H2BC18 | 3.441902921 | -2.268309941 | 1.029305034 | -2.203729571 | 0.027543368 |
| HSD3B1 | 4.681593038 | -2.736132306 | 1.034830955 | -2.644037941 | 0.008192347 |
| RTL8A | 250.2904793 | -1.813057827 | 0.503357747 | -3.601926936 | 0.000315867 |
| ARRDC1-AS1 | 14.5654023 | -1.683370534 | 0.693191182 | -2.428436161 | 0.015164094 |
| LOC100287792 | 3.759547333 | 1.815254712 | 0.851897118 | 2.130837956 | 0.033102494 |
| TMEM273 | 5.570016231 | 2.251974603 | 0.855337229 | 2.632849976 | 0.008467174 |
| HSD17B8 | 35.89607597 | -1.222716846 | 0.521324665 | -2.345403793 | 0.019006483 |
| OXLD1 | 21.34353212 | -1.911289706 | 0.580990865 | -3.289706986 | 0.001002918 |
| LINC02731 | 8.490851469 | 1.353666119 | 0.596536603 | 2.269208815 | 0.02325563 |
| PSMB8 | 8.287164872 | -1.764443131 | 0.742510751 | -2.376319978 | 0.017486292 |
| AGPAT1 | 12.54322234 | -2.991366365 | 0.950368825 | -3.147584693 | 0.001646254 |
| PJVK | 21.99148286 | -0.874481495 | 0.396282409 | -2.206712879 | 0.027334125 |
| NELFE | 343.1413346 | -1.822491861 | 0.794194151 | -2.294768677 | 0.021746385 |
| VARS1 | 44.75220717 | -1.247591253 | 0.572304854 | -2.179941766 | 0.029261778 |
| PRRC2A | 40.94605618 | -1.998173328 | 0.643086996 | -3.107158658 | 0.00188895 |
| ZNF551 | 151.9278438 | 0.57798274 | 0.245973412 | 2.349777305 | 0.018784646 |
| ZNF155 | 17.39861417 | 0.910316011 | 0.441098607 | 2.063747191 | 0.039041692 |
| FAM221B | 7.05738912 | 1.364375285 | 0.56737033 | 2.404734988 | 0.016184197 |
| PSG5 | 115.0464944 | -0.905219377 | 0.35998422 | -2.514608492 | 0.011916463 |
| ZNF783 | 28.19394313 | 0.738450535 | 0.348980102 | 2.116024757 | 0.034342702 |
| PCDHA4 | 17.43197717 | 1.168976046 | 0.59539017 | 1.963378143 | 0.049602252 |
| LINC02138 | 2.850904549 | 2.295320893 | 1.151067343 | 1.994080457 | 0.046143263 |
| C4orf47 | 136.9000504 | -0.463827985 | 0.206430191 | -2.246899947 | 0.024646422 |
| PSENEN | 84.35008026 | -0.583060238 | 0.297151215 | -1.962166763 | 0.049743072 |
| PSMB10 | 4.7874097 | -2.225392406 | 1.073974832 | -2.072108525 | 0.038255321 |
| SARNP | 5.645510574 | -4.194793068 | 1.213891033 | -3.455658665 | 0.00054895 |
| OR6C72P | 2.936910364 | 3.49174805 | 1.256874156 | 2.778120652 | 0.005467431 |
| SLCO6A1 | 2.963987609 | -3.711429114 | 1.138053528 | -3.261207864 | 0.001109387 |
| KRT6A | 4.136502472 | -1.94568208 | 0.855290315 | -2.274879122 | 0.022913182 |
| KRT81 | 44.23606074 | -1.439475913 | 0.597455937 | -2.409342388 | 0.015981297 |
| LINC00898 | 2.876912411 | 2.253383363 | 0.960883344 | 2.345116478 | 0.019021136 |
| LOC344967 | 2.867203811 | 3.075510455 | 1.336690778 | 2.300839135 | 0.021400725 |
| CYS1 | 3.985420278 | -2.02545224 | 0.877750285 | -2.30754951 | 0.021024205 |
| RNPS1 | 348.4210832 | -0.762991046 | 0.322586575 | -2.365228761 | 0.018018937 |
| CD200R1L | 9.822836034 | -1.40771877 | 0.633040773 | -2.223741075 | 0.02616587 |
| MIR618 | 4744711.264 | 23.30045487 | 0.634373599 | 36.7298622 | 2.44E-295 |
| SLC48A1 | 55.80332958 | -1.608765288 | 0.588665301 | -2.732903204 | 0.006277878 |
| TAS2R15P | 22.59649141 | -1.113675575 | 0.470186941 | -2.368580406 | 0.017856499 |
| KRTAP2-3 | 27.83739895 | -1.603391535 | 0.705794868 | -2.271752895 | 0.023101439 |
| MAP10 | 10.58576379 | 2.081280542 | 0.806831924 | 2.579571384 | 0.009892301 |
| ZNF580 | 22.59837556 | -2.186956433 | 0.717483663 | -3.048092305 | 0.002302992 |
| CGB8 | 6.100998045 | -3.67674125 | 1.345586318 | -2.732445479 | 0.006286608 |
| ZNF254 | 189.4290227 | -0.506058697 | 0.160991648 | -3.143384783 | 0.001670061 |
| ANKRD39 | 50.02363388 | -1.598628917 | 0.398027124 | -4.016381848 | 5.91E-05 |
| QTRT1 | 8.404364469 | -1.99664362 | 0.749052126 | -2.665560313 | 0.007686014 |
| ARL2 | 165.1380169 | -2.535285521 | 0.871624166 | -2.908691176 | 0.003629452 |
| SRA1 | 901.5914098 | -1.096166608 | 0.416802921 | -2.629939842 | 0.008539998 |
| RPLP0P6 | 79.30739932 | -1.219531698 | 0.511432461 | -2.384541052 | 0.01710044 |
| NDUFS3 | 1132.865975 | -0.476123836 | 0.211340292 | -2.25287772 | 0.02426686 |
| SLC35F6 | 104.607547 | -1.155678972 | 0.513602403 | -2.250143233 | 0.024439854 |
| DDAH2 | 10.71051669 | -2.476862598 | 0.814622196 | -3.040504679 | 0.00236182 |
| RPS3AP20 | 6.252234078 | -1.689058402 | 0.730822327 | -2.311175153 | 0.020823182 |
| DDX47 | 5.675226282 | 1.212859331 | 0.586924985 | 2.066463963 | 0.038784692 |
| NUDT19 | 6.208858641 | -1.399273168 | 0.697855989 | -2.005103046 | 0.044952032 |
| ARL16 | 816.9869716 | -0.860395534 | 0.402549311 | -2.137366806 | 0.032568169 |
| ALG3 | 186.4970493 | -1.839614519 | 0.726461037 | -2.532296193 | 0.011331822 |
| FOXI3 | 24.02475311 | 1.227686142 | 0.412522442 | 2.976046921 | 0.002919902 |
| LINC01347 | 3.575286562 | -2.134819132 | 1.048115235 | -2.036817194 | 0.041668365 |
| PHB2 | 295.4321899 | -0.77989895 | 0.327571649 | -2.380849965 | 0.017272745 |
| LOC652276 | 15.00934559 | -1.595656392 | 0.722411953 | -2.208790131 | 0.027189242 |
| LINC02649 | 8.1722996 | -1.892024403 | 0.673016046 | -2.81126195 | 0.004934759 |
| TMED11P | 4.995944668 | 1.35170599 | 0.616530596 | 2.192439432 | 0.028347792 |
| CROCCP2 | 139.9454449 | -0.527592543 | 0.259044735 | -2.036685068 | 0.041681612 |
| HSPD1P10 | 4.675590124 | 2.87322683 | 1.166771715 | 2.462544123 | 0.01379552 |
| TENM3 | 192.0559284 | -0.400116508 | 0.200246416 | -1.998120698 | 0.045703576 |
| C2orf16 | 33.12768377 | 0.893524738 | 0.424950687 | 2.102655118 | 0.035495928 |
| NPTXR | 17.4372449 | -1.254101429 | 0.579576788 | -2.163822734 | 0.030477957 |
| RNU6-101P | 3.106987948 | -3.044394267 | 1.172484338 | -2.596532992 | 0.009416986 |
| RNY1P15 | 13.86819027 | -1.269332395 | 0.435591205 | -2.914045049 | 0.003567784 |
| TVP23CP1 | 8.607951521 | -1.662963166 | 0.629630182 | -2.641174477 | 0.008261916 |
| CACNA2D1-AS1 | 4.18754425 | -2.061846894 | 0.989762715 | -2.08317293 | 0.037235464 |
| CPTP | 8.512822636 | -1.953913739 | 0.984437075 | -1.98480308 | 0.047166385 |
| LINC01708 | 3.478435459 | 2.409355852 | 1.026374267 | 2.347443744 | 0.018902727 |
| TMLHE-AS1 | 3.417391992 | -2.838893055 | 1.301408109 | -2.18140108 | 0.029153764 |
| LOC101927830 | 3.417391992 | -2.838893055 | 1.301408109 | -2.18140108 | 0.029153764 |
| RNPC3-DT | 7.712593464 | 2.171829244 | 1.05291317 | 2.06268599 | 0.039142472 |
| LOC339685 | 5.571953979 | 1.390397242 | 0.645169018 | 2.155089911 | 0.031154799 |
| OPA1-AS1 | 4.250204301 | -1.565669865 | 0.752408787 | -2.080876636 | 0.037445199 |
| LINC01266 | 4.480320471 | -2.374665842 | 0.826689089 | -2.872501734 | 0.004072358 |
| PPP1R8P1 | 2.938919145 | -2.094777891 | 0.986186109 | -2.124120257 | 0.033660089 |
| LOC124900513 | 81.20646883 | -0.983842805 | 0.28288923 | -3.477837618 | 0.000505476 |
| LINC02470 | 7.183042691 | -3.438832214 | 1.027503411 | -3.346784232 | 0.000817548 |
| MTND2P28 | 174.3572718 | -0.857526226 | 0.283002197 | -3.030104482 | 0.002444692 |
| MCRIP1 | 113.3043165 | -1.930108245 | 0.900470086 | -2.143445157 | 0.03207737 |
| NEK4P1 | 3.244216802 | 3.707746444 | 1.230540343 | 3.013104337 | 0.0025859 |
| HCG23 | 9.724497799 | 2.085396726 | 0.52835511 | 3.946960458 | 7.91E-05 |
| TSBP1-AS1 | 9.724497799 | 2.085396726 | 0.52835511 | 3.946960458 | 7.91E-05 |
| LINC02097 | 4.129488282 | 2.925985571 | 0.916804163 | 3.191505545 | 0.001415334 |
| FTCDNL1 | 34.24550978 | 1.060461565 | 0.3373142 | 3.143839087 | 0.001667471 |
| LINC01907 | 8.071105577 | 1.574867568 | 0.753859968 | 2.089071756 | 0.036701264 |
| UBE2V2P1 | 3.248801581 | 2.057301496 | 0.854164005 | 2.408555598 | 0.016015787 |
| TMEM191A | 15.24494212 | -1.383705035 | 0.575752564 | -2.403298086 | 0.016247937 |
| ZKSCAN8P1 | 33.5679161 | -1.407414706 | 0.474713511 | -2.964766482 | 0.003029128 |
| RPS3AP38 | 10.417451 | 2.144482404 | 0.885461754 | 2.42188033 | 0.015440434 |
| ANKRD18DP | 8.760719776 | -1.024054076 | 0.518286204 | -1.97584668 | 0.048172145 |
| ENTPD1-AS1 | 22.5291027 | -0.814919816 | 0.396206614 | -2.056805176 | 0.03970497 |
| NCKAP5-AS2 | 4.013031898 | 2.406318403 | 0.98094718 | 2.453056038 | 0.014164827 |
| ZNF717 | 68.30265867 | 0.524807546 | 0.260134866 | 2.017444081 | 0.043649191 |
| LINC01068 | 5.075302617 | -4.023112049 | 1.022349005 | -3.935165027 | 8.31E-05 |
| LYPLAL1-DT | 9.517754998 | -1.550518301 | 0.734431007 | -2.111183062 | 0.03475658 |
| LINC01649 | 5.210010919 | 2.690804932 | 0.869596957 | 3.094312728 | 0.001972694 |
| ATP8 | 46.6390269 | -0.842782838 | 0.315734985 | -2.669272895 | 0.007601566 |
| PCAT6 | 2.978197498 | -3.240676657 | 1.453984333 | -2.228825018 | 0.02582555 |
| FAM174C | 95.01174337 | -2.836667409 | 1.049143336 | -2.703793954 | 0.006855278 |
| REL-DT | 3.823332518 | 1.772011492 | 0.8966272 | 1.976307982 | 0.048119906 |
| TTC3-AS1 | 3.107706298 | -2.569896245 | 1.204579431 | -2.133438592 | 0.032888764 |
| RPS3AP30 | 5.16366855 | -2.916806441 | 1.096764819 | -2.659463898 | 0.007826512 |
| EPHA1-AS1 | 9.674898184 | 1.118555808 | 0.552236043 | 2.025503084 | 0.042815732 |
| LINC02829 | 3.36764816 | 2.472198354 | 1.216998078 | 2.031390516 | 0.042215396 |
| RFPL4AL1 | 3.810585358 | 2.547505879 | 1.200831527 | 2.12145153 | 0.033883823 |
| LOC100419859 | 3.691157121 | 3.620810627 | 1.012851774 | 3.574867243 | 0.000350406 |
| MIR5689HG | 3.210766622 | -2.430190194 | 1.075065287 | -2.260504757 | 0.023789942 |
| LINC02264 | 3.193477299 | -4.370379771 | 1.461721837 | -2.989884709 | 0.002790828 |
| ZNF688 | 5.41650933 | -2.985355436 | 1.032776447 | -2.890611463 | 0.003844932 |
| LOC107985211 | 4.200647658 | -1.739979064 | 0.759594461 | -2.290668446 | 0.021982597 |
| RPS3AP7 | 4.12016887 | -4.730331791 | 2.292198308 | -2.063666033 | 0.039049392 |
| LINC01036 | 7.29298072 | -1.281021604 | 0.653093344 | -1.961467859 | 0.049824471 |
| LINC00309 | 6.499845759 | -1.931882976 | 0.879137043 | -2.197476482 | 0.027986434 |
| CFAP58-DT | 6.504885775 | 2.442276211 | 0.976270977 | 2.501637629 | 0.012362038 |
| EHBP1-AS1 | 6.279006153 | -2.039351448 | 0.717909846 | -2.840679035 | 0.00450176 |
| TSPAN19 | 6.225755537 | -1.712132702 | 0.808309727 | -2.118164171 | 0.034161166 |
| PSG1 | 320.5871538 | -0.523641959 | 0.256778824 | -2.039272359 | 0.041422853 |
| RPS15AP12 | 12.99260391 | 1.933099654 | 0.564575121 | 3.423990149 | 0.000617089 |
| LINC00867 | 10.62129211 | -1.227974894 | 0.520186169 | -2.360645031 | 0.018243183 |
| LINC02518 | 2.902679249 | -2.933846856 | 1.329981867 | -2.205929967 | 0.027388904 |
| LINC00665 | 252.9732159 | -0.382242243 | 0.179670576 | -2.127461558 | 0.033381752 |
| RGS5-AS1 | 4.243305663 | -2.954760706 | 1.060138028 | -2.787147171 | 0.005317432 |
| SNHG15 | 214.4255702 | -0.543867055 | 0.257569269 | -2.111537056 | 0.034726176 |
| RPS15AP6 | 5.332581178 | -2.261281374 | 0.82512193 | -2.740542086 | 0.006133793 |
| LOC339260 | 10.18866007 | 1.215018063 | 0.560026235 | 2.169573474 | 0.030039173 |
| CCDC144NL-AS1 | 10.18866007 | 1.215018063 | 0.560026235 | 2.169573474 | 0.030039173 |
| MROH3P | 4.256886073 | 2.413429617 | 0.89626531 | 2.692762499 | 0.007086273 |
| LOC101928401 | 2.993782538 | 3.059167073 | 1.270692404 | 2.407480413 | 0.016063024 |
| ATP6V1G1P4 | 4.453685341 | -2.611847091 | 0.996904521 | -2.619957113 | 0.008794083 |
| MAP3K5-AS1 | 4.959300711 | -1.653713494 | 0.840694659 | -1.967079815 | 0.04917401 |
| FTH1P2 | 149.3291007 | -1.557672914 | 0.769447473 | -2.024404484 | 0.042928551 |
| RPS3AP28 | 3.223658123 | -3.200388215 | 1.515064878 | -2.112377008 | 0.034654125 |
| DAAM2-AS1 | 3.308657363 | 1.979402533 | 0.951180088 | 2.080996605 | 0.037434217 |
| LOC101927020 | 3.711057942 | -3.633698779 | 1.178608711 | -3.083040831 | 0.00204897 |
| PPP1R3E | 23.1665907 | -0.78393557 | 0.385606032 | -2.032996127 | 0.042052914 |
| KRT18P63 | 3.881188662 | -2.635623805 | 1.086792762 | -2.425139269 | 0.015302515 |
| LINC01278 | 114.0152097 | -0.956859454 | 0.370020778 | -2.585961412 | 0.00971078 |
| LURAP1L-AS1 | 48.54157682 | -0.981937235 | 0.482795161 | -2.0338589 | 0.041965824 |
| EPM2A-DT | 116.7305504 | -0.735152029 | 0.330374304 | -2.225209467 | 0.02606718 |
| LOC105373346 | 4.364072338 | 2.288535991 | 1.002676693 | 2.282426635 | 0.022464164 |
| RPL13AP12 | 9.153234958 | -2.33246087 | 0.951678884 | -2.45089064 | 0.014250324 |
| LINC01920 | 4.29059104 | -2.29591386 | 0.859509584 | -2.67119053 | 0.007558273 |
| LINC01447 | 4.829785112 | -3.846565382 | 1.109324789 | -3.467483482 | 0.000525356 |
| LINC01376 | 12.60209214 | -1.11285214 | 0.476324269 | -2.336333067 | 0.019473889 |
| LINC01695 | 4.225650679 | -2.112849547 | 0.970694602 | -2.176636753 | 0.029507681 |
| ELOA-AS1 | 15.83301458 | -1.195469065 | 0.431019747 | -2.773583049 | 0.005544269 |
| CLCA4-AS1 | 6.289884133 | -1.514083062 | 0.73204073 | -2.068304398 | 0.038611408 |
| DDX10P1 | 3.091021449 | -2.753196931 | 0.994086538 | -2.769574706 | 0.005612953 |
| LINC00851 | 2.937210951 | -3.701470349 | 1.41943369 | -2.60770924 | 0.009115034 |
| ZNF737 | 64.91311824 | -0.446511049 | 0.221464203 | -2.016177072 | 0.043781463 |
| OR7E24 | 3.349937622 | -2.532692887 | 1.227032365 | -2.064079938 | 0.039010138 |
| MTCO2P19 | 5.071238711 | -1.810985777 | 0.830176926 | -2.18144557 | 0.029150476 |
| SCIRT | 3.015262958 | -1.825187609 | 0.858262258 | -2.126608263 | 0.033452645 |
| LINC01143 | 3.515173698 | 2.449310457 | 1.208740715 | 2.026332385 | 0.042730735 |
| LOC101927609 | 13.62421241 | -1.342436517 | 0.554014056 | -2.423109127 | 0.015388303 |
| RPL37P12 | 4.273772462 | -2.463428612 | 1.149132641 | -2.14372869 | 0.032054632 |
| MTATP6P20 | 3.503381248 | -2.790779317 | 1.402312788 | -1.990126126 | 0.046577043 |
| PAXBP1-AS1 | 26.13704894 | -0.863511988 | 0.302382597 | -2.855693399 | 0.004294296 |
| LINC01119 | 10.76249702 | -1.02192359 | 0.516067381 | -1.980213489 | 0.047679545 |
| RN7SL221P | 3.041714477 | 2.470777107 | 1.117502238 | 2.210981798 | 0.027037098 |
| STPG4 | 11.31809211 | -1.063877496 | 0.527761745 | -2.015829125 | 0.043817847 |
| RPL34P28 | 6.815121328 | -1.834781787 | 0.685067703 | -2.678248848 | 0.007400821 |
| TCAM1P | 9.425966632 | -1.267494903 | 0.612209507 | -2.070361353 | 0.038418518 |
| RPS2P5 | 97.90950576 | -1.308303294 | 0.564410314 | -2.318000328 | 0.020449303 |
| SAMMSON | 23.40337633 | -0.683693015 | 0.344938948 | -1.982069637 | 0.047471447 |
| RPL17P38 | 5.137629068 | 1.949839542 | 0.787987095 | 2.474456186 | 0.013343919 |
| LINC02614 | 10.5784467 | -1.294758867 | 0.616739031 | -2.099362617 | 0.035784947 |
| LINC00635 | 10.86477952 | 1.209019766 | 0.557733633 | 2.167736881 | 0.030178712 |
| ARPC4 | 438.2546897 | -0.985212533 | 0.477317192 | -2.064062533 | 0.039011788 |
| SNHG3 | 791.8391058 | 0.306657391 | 0.151759812 | 2.020675876 | 0.043313329 |
| EIF6 | 183.5241786 | -1.43238147 | 0.537036452 | -2.667195986 | 0.007648705 |
| RPL7AP11 | 5.771899048 | 2.023030993 | 0.918966831 | 2.201418946 | 0.027706379 |
| DECR2 | 7.112125203 | -1.77396685 | 0.795903982 | -2.22887043 | 0.025822527 |
| CNTF | 7.691089755 | 1.446748776 | 0.699044425 | 2.069609205 | 0.038488955 |
| RPL23AP55 | 4.176781866 | -4.234631616 | 1.184814105 | -3.574089469 | 0.000351449 |
| KCTD7 | 8.963922135 | -1.420910972 | 0.577870523 | -2.458874291 | 0.013937341 |
| C4orf48 | 52.59003563 | -2.270539753 | 0.928194268 | -2.446190233 | 0.01443748 |
| NME2 | 5.156993516 | -1.98225019 | 0.794303354 | -2.495583305 | 0.012575022 |
| CFAP44-AS1 | 4.331998907 | -4.302079321 | 1.016701938 | -4.231406628 | 2.32E-05 |
| MRPS6 | 659.3240447 | -0.400666353 | 0.193523407 | -2.070376702 | 0.038417082 |
| PPIEL | 16.01025272 | -1.125203369 | 0.54477239 | -2.065455938 | 0.03887988 |
| FAM3D-AS1 | 3.603637578 | 2.186521433 | 0.992223676 | 2.203657789 | 0.02754842 |
| APOBEC3C | 28.08816957 | -1.281456551 | 0.597635022 | -2.14421261 | 0.032015855 |
| PTCHD4 | 19.17801127 | 1.0093332 | 0.476162868 | 2.119722617 | 0.034029445 |
| RPSAP29 | 7.207908618 | 2.930376128 | 0.822815995 | 3.561399083 | 0.000368884 |
| CYP2U1-AS1 | 18.40784815 | -1.313588386 | 0.439190775 | -2.99092891 | 0.002781302 |
| LOC107986298 | 18.40784815 | -1.313588386 | 0.439190775 | -2.99092891 | 0.002781302 |
| FRG1-DT | 17.59204869 | 0.929271055 | 0.362059099 | 2.566628092 | 0.010269269 |
| GS1-24F4.2 | 17.27082651 | 1.15125153 | 0.423966482 | 2.715430532 | 0.006618966 |
| LINC02325 | 5.225444716 | 2.075422385 | 0.772983781 | 2.684949461 | 0.007254077 |
| MCPH1-DT | 43.62044272 | 0.635301999 | 0.256534312 | 2.47647963 | 0.01326852 |
| SBF2-AS1 | 35.93480502 | -0.689247664 | 0.339950862 | -2.027492029 | 0.042612119 |
| LOC101929290 | 14.85559363 | -1.467834693 | 0.455118403 | -3.225171037 | 0.001258974 |
| LINC02288 | 4.201491803 | 1.971048603 | 0.813919719 | 2.421674469 | 0.015449183 |
| LINC01252 | 5.537715947 | 1.611595342 | 0.728063089 | 2.213538039 | 0.026860574 |
| TWF2 | 38.27628849 | -1.486184648 | 0.633510983 | -2.345949301 | 0.018978689 |
| PCED1B-AS1 | 23.18990832 | -1.122036915 | 0.471273953 | -2.380859176 | 0.017272314 |
| NRAV | 14.21697519 | -1.190125296 | 0.50760359 | -2.344595899 | 0.019047711 |
| FAM13A-AS1 | 41.33843919 | -0.721794453 | 0.338509175 | -2.132274412 | 0.032984294 |
| ARHGAP42-AS1 | 4.32692195 | -2.261511947 | 1.001693954 | -2.257687528 | 0.023965147 |
| FGF10-AS1 | 3.367304542 | 2.99716104 | 1.06250138 | 2.820853786 | 0.004789603 |
| MTATP6P1 | 2638.666314 | -0.713502431 | 0.212650439 | -3.355283136 | 0.000792838 |
| LINC02432 | 247.3020835 | -0.717338883 | 0.209745497 | -3.420044263 | 0.000626109 |
| PCDHA11 | 12.53251862 | 1.514974453 | 0.476685442 | 3.178142903 | 0.001482217 |
| CASC11 | 9.860439991 | -2.590977896 | 0.909963607 | -2.847342327 | 0.004408593 |
| LINC00942 | 5.354721987 | 1.31134621 | 0.626101655 | 2.094462138 | 0.036218832 |
| LINC01265 | 44.17629046 | 0.568163567 | 0.23674248 | 2.399922341 | 0.01639855 |
| RNF103-CHMP3 | 14.73304752 | -1.087034453 | 0.480867501 | -2.260569594 | 0.023785922 |
| LOC100289037 | 3.773960186 | -4.095115989 | 1.618326217 | -2.530463849 | 0.011391182 |
| ZNF718 | 74.07848239 | -0.68102954 | 0.318049543 | -2.141268728 | 0.032252373 |
| PURPL | 51.53756026 | -0.984214207 | 0.356554936 | -2.760343797 | 0.005774056 |
| LINC02200 | 5.522708281 | 2.261725661 | 0.701785459 | 3.222816364 | 0.001269369 |
| LINC02105 | 6.944179675 | 1.810037411 | 0.753505461 | 2.402155664 | 0.016298771 |
| PPM1AP1 | 10.78854088 | -1.573081071 | 0.747517554 | -2.104406862 | 0.035342971 |
| FAM218A | 21.63615815 | 0.913271645 | 0.402591122 | 2.268484312 | 0.023299704 |
| LINC02701 | 5.549577708 | -1.664180454 | 0.793363157 | -2.097627598 | 0.035938055 |
| LINC02492 | 5.520098831 | -2.105555794 | 0.910511883 | -2.312496775 | 0.020750323 |
| LOC105377267 | 9.889868088 | -1.299441126 | 0.646095406 | -2.011221741 | 0.04430204 |
| LINC02493 | 4.111998678 | 1.943084694 | 0.831294938 | 2.337419135 | 0.019417401 |
| LOC105374438 | 4.795558234 | 2.113217978 | 0.93153423 | 2.26853497 | 0.02329662 |
| F11-AS1 | 4.771452196 | 1.575522984 | 0.791532632 | 1.990471296 | 0.046539043 |
| LINC02728 | 7.601701134 | -1.327946275 | 0.611641707 | -2.171117926 | 0.029922259 |
| YTHDF1P1 | 4.506817483 | -3.864279459 | 1.507497651 | -2.563373453 | 0.010366048 |
| RASGRF2-AS1 | 4.746259433 | 1.520767676 | 0.695731641 | 2.185853836 | 0.028826301 |
| RNU6-531P | 4.075099048 | 1.937471393 | 0.914980314 | 2.117500632 | 0.034217382 |
| RPSAP74 | 10.41361149 | -1.248020083 | 0.571786712 | -2.182667169 | 0.029060329 |
| MINCR | 31.34749035 | -0.901095095 | 0.352025815 | -2.559741522 | 0.010475004 |
| LOC105377730 | 5.59615969 | -1.746139252 | 0.765252035 | -2.28178322 | 0.022502142 |
| PCDHGB2 | 7.436618123 | -1.401246175 | 0.71047559 | -1.972265048 | 0.048579357 |
| LINC01591 | 3.291056167 | 2.370417611 | 0.872447309 | 2.716975096 | 0.006588156 |
| NPIPB11 | 5.441982552 | -1.713158815 | 0.805047094 | -2.128023103 | 0.033335168 |
| UQCRB-AS1 | 4.660564646 | -2.63346568 | 1.001614767 | -2.629220101 | 0.008558095 |
| MIR2052HG | 6.359509507 | -1.648593623 | 0.717865038 | -2.296523074 | 0.021645991 |
| LOC101927141 | 5.103897187 | 1.89046134 | 0.916759477 | 2.062112679 | 0.03919701 |
| PBOV1 | 11.41897168 | -1.95135728 | 0.97750919 | -1.996254663 | 0.04590621 |
| NAV2-AS3 | 5.295342939 | 3.074474537 | 0.989387854 | 3.107451265 | 0.001887081 |
| LOC100420800 | 3.938931452 | -2.804417623 | 1.398295377 | -2.005597436 | 0.044899216 |
| MPV17L2 | 32.07195469 | -1.330037512 | 0.569393257 | -2.335885603 | 0.019497204 |
| LOC107984322 | 3.519552031 | 1.64931186 | 0.818305424 | 2.015521115 | 0.043850076 |
| URB1-AS1 | 96.55834846 | -1.326369075 | 0.370837758 | -3.576682919 | 0.000347982 |
| FAM133GP | 2.822403152 | 2.680108774 | 1.155501141 | 2.319434121 | 0.020371509 |
| TAS2R64P | 13.56091767 | -1.660742489 | 0.572048212 | -2.903151264 | 0.003694282 |
| CLEC12B | 4.305774422 | -4.796032711 | 1.069327683 | -4.485091694 | 7.29E-06 |
| LINC02293 | 5.542268378 | 2.593623188 | 0.848680299 | 3.056066216 | 0.002242617 |
| ZNF878 | 15.54802884 | 0.828050411 | 0.379770889 | 2.180394643 | 0.029228221 |
| AQP5-AS1 | 3.159114866 | 2.53424339 | 0.990350747 | 2.558935205 | 0.010499331 |
| LOC124902964 | 4.533160674 | 1.622820743 | 0.765286134 | 2.120541156 | 0.033960435 |
| MAP1LC3B2 | 5.011630416 | -1.613740897 | 0.765087755 | -2.109223272 | 0.034925314 |
| BMS1P16 | 3.815214678 | 2.61038334 | 0.888071377 | 2.939384611 | 0.003288647 |
| LOC105371022 | 5.384319481 | 2.142879233 | 0.875470706 | 2.447688106 | 0.014377605 |
| HNRNPMP1 | 7.324889959 | -2.50365527 | 1.042676241 | -2.401181855 | 0.016342213 |
| LOC105370854 | 9.521867495 | -1.588708419 | 0.542868945 | -2.926504515 | 0.003427945 |
| CPEB1-AS1 | 4.900145545 | 2.056623525 | 0.819746227 | 2.508853906 | 0.012112357 |
| ZFHX3-AS1 | 45.22324359 | 0.516428111 | 0.253377307 | 2.03817823 | 0.041532112 |
| LOC102723670 | 7.599944869 | -1.407449841 | 0.619039745 | -2.27360174 | 0.022989943 |
| CASC17 | 4.907035135 | -1.748446486 | 0.807208803 | -2.166039914 | 0.030308137 |
| CAPN10-DT | 13.79917531 | -1.753992641 | 0.678417607 | -2.585417335 | 0.009726119 |
| WFDC21P | 28.77109808 | 1.06663372 | 0.390159198 | 2.733842299 | 0.006260002 |
| LINC02133 | 5.10029947 | 1.919498666 | 0.760312123 | 2.524619308 | 0.011582368 |
| ATP2C2-AS1 | 4.092470434 | 2.262173169 | 0.910220507 | 2.485302356 | 0.012944144 |
| TBILA | 25.22149592 | -1.010065045 | 0.477355786 | -2.115958524 | 0.034348335 |
| LINC02180 | 4.499161292 | -2.671321023 | 0.970789392 | -2.751699849 | 0.005928683 |
| LINC01979 | 5.093194249 | 1.442696731 | 0.628891927 | 2.294029658 | 0.021788795 |
| MTND1P8 | 9.431237679 | 2.379385435 | 0.866773582 | 2.745106084 | 0.006049134 |
| DPEP2NB | 4.117761087 | -3.501774737 | 1.441041516 | -2.430030432 | 0.015097555 |
| LINC01919 | 3.996753568 | -2.229482085 | 0.981044408 | -2.272559801 | 0.02305272 |
| GDF2 | 2.908226477 | -3.68268849 | 1.104967529 | -3.332847701 | 0.00085962 |
| VSTM2B-DT | 3.92908486 | 1.616179109 | 0.80920393 | 1.997245749 | 0.045798494 |
| TIMM23 | 463.1285189 | -0.321512864 | 0.120857393 | -2.660266419 | 0.007807886 |
| ZNF415P1 | 6.65734562 | 2.230081177 | 1.031913329 | 2.161112872 | 0.030686622 |
| NBPF15 | 75.87827342 | -0.811551159 | 0.255503751 | -3.176278844 | 0.001491775 |
| C17orf113 | 4.51958465 | -1.622842707 | 0.803995299 | -2.018472882 | 0.043542036 |
| LOC105372390 | 2.867014688 | -2.912839947 | 1.109146254 | -2.62620005 | 0.008634405 |
| HEATR6-DT | 3.897941665 | 1.592163309 | 0.809459184 | 1.966946994 | 0.049189322 |
| LINC01864 | 3.832271146 | -2.197421814 | 0.921248021 | -2.385266251 | 0.017066764 |
| S1PR2 | 9.381442055 | -1.563458989 | 0.605249745 | -2.583163398 | 0.009789894 |
| GNA15-DT | 3.263676135 | -2.050442284 | 0.99470771 | -2.061351554 | 0.039269513 |
| LINC01028 | 3.213683869 | -4.377865531 | 1.418149974 | -3.087025782 | 0.002021701 |
| LINC01482 | 5.532832392 | 1.594927226 | 0.66691985 | 2.391482614 | 0.016780479 |
| LINC02078 | 3.355125017 | 1.681645924 | 0.825958249 | 2.035993861 | 0.041750972 |
| CARD8-AS1 | 46.03028665 | 0.869651028 | 0.238466812 | 3.646843012 | 0.000265482 |
| LOC100129935 | 3.006247304 | 3.283739682 | 1.053959975 | 3.11562086 | 0.001835581 |
| HAVCR1P1 | 11.02237909 | 1.798976788 | 0.680094769 | 2.645185451 | 0.008164616 |
| LINC01711 | 35.15777654 | -1.202577036 | 0.479188879 | -2.509609655 | 0.012086468 |
| LINC00528 | 3.903165361 | 2.970732502 | 0.971170008 | 3.058921174 | 0.002221356 |
| RAB11B-AS1 | 11.79337454 | -1.103497005 | 0.505839131 | -2.181517674 | 0.029145149 |
| CCNYL6 | 8.329757565 | -2.054172387 | 0.668728809 | -3.071756982 | 0.002128029 |
| SNHG8 | 659.2857443 | 0.572443924 | 0.200047606 | 2.861538483 | 0.004215903 |
| NCBP2AS2 | 292.9209091 | -1.230337852 | 0.468761267 | -2.624657663 | 0.008673613 |
| HMGN3-AS1 | 50.42815003 | 0.906521613 | 0.358518576 | 2.528520622 | 0.011454436 |
| TAF15 | 273.8764274 | -0.617864724 | 0.266452156 | -2.318858042 | 0.020402735 |
| LOC100130664 | 3.691257949 | 2.508332834 | 1.021913353 | 2.454545511 | 0.014106281 |
| HNRNPA3P9 | 10.708056 | -1.898368526 | 0.958467387 | -1.980629233 | 0.047632868 |
| BNIP3P11 | 9.584588381 | 1.122163454 | 0.551713854 | 2.033959173 | 0.041955712 |
| LINC01607 | 6.37843707 | 1.371014209 | 0.673641346 | 2.035228712 | 0.041827865 |
| NBPF25P | 12.96801568 | -0.998582203 | 0.501327522 | -1.991875888 | 0.046384677 |
| SNORA28 | 13.09285485 | -1.261438754 | 0.601417259 | -2.097443554 | 0.035954329 |
| NBPF26 | 20.76789169 | -1.028113993 | 0.41706139 | -2.46513827 | 0.013696039 |
| LOC105375519 | 12.43035546 | 1.158068458 | 0.499256833 | 2.319584594 | 0.02036336 |
| LOC100419515 | 4.533080073 | -1.958757759 | 0.908319153 | -2.156464225 | 0.031047434 |
| LOC158434 | 6.328658008 | -2.108484165 | 0.915810036 | -2.302316072 | 0.021317354 |
| SEPTIN7P12 | 12.35984211 | -1.666786189 | 0.646840063 | -2.576813472 | 0.009971574 |
| H3C1 | 5.266246307 | -2.019561122 | 0.767894399 | -2.629998506 | 0.008538524 |
| LOC101929494 | 102.1924075 | -0.699543143 | 0.340807789 | -2.052603157 | 0.040111081 |
| PADI6 | 4.53363629 | 2.079412633 | 0.944230957 | 2.202228827 | 0.027649149 |
| CISD3 | 12.98936365 | -1.66539155 | 0.639229203 | -2.605312058 | 0.009179062 |
| GGNBP2 | 1397.808118 | 0.327013067 | 0.160904622 | 2.032341034 | 0.042119143 |
| LINC00624 | 326.0295789 | -0.467934876 | 0.190019354 | -2.462564296 | 0.013794744 |
| H3C10 | 14.64650722 | -1.2218502 | 0.541658129 | -2.255759 | 0.024085728 |
| OR2AG1 | 6.049370326 | -2.109132926 | 0.830710339 | -2.538951097 | 0.011118536 |
| MAPT-IT1 | 4.005696649 | 2.326415987 | 1.027184189 | 2.264847934 | 0.023522013 |
| LINC00628 | 3.283944135 | 2.746689592 | 1.097662863 | 2.502307114 | 0.012338684 |
| HYMAI | 13.74456367 | 1.076963097 | 0.488672816 | 2.203853094 | 0.027534677 |
| C13orf46 | 4.498767379 | 1.873897822 | 0.703465608 | 2.663808722 | 0.007726148 |
| RELA-DT | 37.32061064 | -0.684460342 | 0.334919031 | -2.043659146 | 0.040987232 |
| LOC105376121 | 7.68093692 | 2.184470979 | 0.786659298 | 2.776895899 | 0.005488075 |
| LOC124905143 | 5.103776072 | -2.315792313 | 1.03038421 | -2.247503688 | 0.024607855 |
| CPLANE1-AS1 | 16.13477165 | 1.145841836 | 0.505611583 | 2.266249181 | 0.023436131 |
| LOC124909397 | 7.505189477 | -1.972546965 | 0.876211349 | -2.251222799 | 0.02437143 |
| LOC105374174 | 9.919089476 | -1.807433687 | 0.641117313 | -2.819193384 | 0.00481445 |
| LOC100289495 | 9.982594796 | 1.673184551 | 0.722848071 | 2.314711234 | 0.020628741 |
| LOC124903585 | 4.682000994 | 2.261409424 | 1.008717108 | 2.241866828 | 0.02496998 |
| H3C3 | 16.17442943 | -0.939009032 | 0.441085476 | -2.128859556 | 0.033265882 |
| LOC124907726 | 9.478113971 | 1.596322554 | 0.552170845 | 2.890993919 | 0.003840256 |
| LOC124902947 | 3.361501521 | 2.068723439 | 0.905007879 | 2.285862351 | 0.022262313 |
| LOC107984016 | 2.778876966 | 2.313622036 | 1.002684337 | 2.307428121 | 0.021030965 |
| LOC100996756 | 7.045157247 | -2.077925551 | 0.947678449 | -2.192648311 | 0.028332728 |
| ZCCHC14-DT | 4.704811162 | -1.765715133 | 0.829496593 | -2.128658691 | 0.033282509 |
| LOC105375713 | 3.246675903 | -2.271025212 | 0.930528997 | -2.440574361 | 0.014663927 |
| LOC105373854 | 4.436696163 | 1.389161411 | 0.658161467 | 2.110669616 | 0.034800719 |
| LOC124901321 | 38.17329052 | -1.271288606 | 0.400475086 | -3.174451172 | 0.001501201 |
