# Supplementary material for: Extrachromosomal circular DNA expressing miRNA promotes ovarian cancer progression
Source: Clin Transl Med. 2025 Sep 23;15(9):e70445. doi: 10.1002/ctm2.70445 (PMC12455017; doi:10.1002/ctm2.70445)
Supplement: Supplementary file 9 — Supporting Information [file CTM2-15-e70445-s004.docx]

**Table S4 Sequence information**

| **COX5B Primer** | **5' - 3'** |
| --- | --- |
| COX5B-F | GGGCACCATTTTCCTTGATCAT |
| COX5B-R | AGTCGCCTGCTCTTCATCAG |
| **Reverse PCR Primers for eccDNA validation** | **5' - 3'** |
| eccMIR3661-PCR-F | TCTCGTCCATGATGCCACC |
| eccMIR3661-PCR-R | AGGTGTTCACCAAGGAGC |
| eccMIR618-PCR-F | GCCAATAACCCATCATCTCTTGG |
| eccMIR618-PCR-R | CTCAGTGTCTGCTATAGTCCCTGAC |
| eccMIR2277-PCR-F | GCCTGCTTGCATCAATAGGTG |
| eccMIR2277-PCR-R | CCACCTGGTAACCGAATGTG |
| **LAMA Synthetic Primers** | **5' - 3'** |
| eccMIR3661-LA-linear A-F | TCTCGTCCATGATGCCACC |
| eccMIR3661-LA-linear A-R | AGGTGTTCACCAAGGAGC |
| eccMIR3661-LA-linear B-F | TCTGGGATACGGCCTAACAAG |
| eccMIR3661-LA-linear B-R | ACATACCTAAAGGTCACACAGC |
| eccMIR618-LA-linear A-F | TAAGGGCTAAGGTAAAACCCAAC |
| eccMIR618-LA-linear A-R | GATGCCTCAGTCGGTTGATGAG |
| eccMIR618-LA-linear B-F | CTGGGAGAGAGGTGATCTGG |
| eccMIR618-LA-linear B-R | GCTGCTTTGGTACCCTTTGATG |
| eccMIR2277-LA-linear A-F | GCCTGCTTGCATCAATAGGTG |
| eccMIR2277-LA-linear A-R | CCACCTGGTAACCGAATGTG |
| eccMIR2277-LA-linear B-F | ACATTTGGCGGCAATTAGAGC |
| eccMIR2277-LA-linear B-R | GGCTGTAAGCTTGGGAAATGG |
| **LAMA Synthetic Sequence** | **5' - 3'** |
| random-linearA | GCTGATCGGTAGGGAGACCGAGAATCTATCGGGCTATGTCACTAAAACTTTCCAAACACCCCGTGTCGATACTGAACGAATCGATGCACACTCCCTTCCTTGAAAACGCACAATCATACAAGTGGGCACATGATGCGTACGCCCATCTAATACATCCAACTCTCTACGCCCTCTTCAAGAGCTGGAAGGGCACCCTGCACTTGGATAAGTGATAACCTCGTAAGGCAAGCTCGTACCGTCATTCATGCGGAAGAGTTAAGACCATTGGAAGTAGGGATAGTTTCGAACCTCGGTTACTAGTCCCAATAAGGGAACCCTGTCTGAAGGATGAGTGTCAGCCAGTGTAACCCGATGAGGTACCCAGAAGTCGAACTGGGCCAGACAACCCGGCGCTAACGCACTCAAACCCGGGGCCCGACGCGACATATCAGCTAAGAGTAGGCCGGGAGTGTAGACCTTTGGGGTTGAATAAATCTATTGTACTAATCGGCTTCAACGAGCCGTACAGGTGGCACCTCAGGAGGGGCCCGCAGGGAGGAAGTAAACTGCTATTCGTCGCCGTTGGTGGTAACTAATTGTGTTCCTTGCCACTACAATTGTATCTAAGCCGTGTAATGAGAACAACCACACCTTAGCGAATTGATGCGCCGCTTCGGAATACCGTTTTGGCTACCCGTTACTAAGCCCATCGCGATTTTCA |
| random-linearB | CAGCCAGTGTAACCCGATGAGGTACCCAGAAGTCGAACTGGGCCAGACAACCCGGCGCTAACGCACTCAAACCCGGGGCCCGACGCGACATATCAGCTAAGAGTAGGCCGGGAGTGTAGACCTTTGGGGTTGAATAAATCTATTGTACTAATCGGCTTCAACGAGCCGTACAGGTGGCACCTCAGGAGGGGCCCGCAGGGAGGAAGTAAACTGCTATTCGTCGCCGTTGGTGGTAACTAATTGTGTTCCTTGCCACTACAATTGTATCTAAGCCGTGTAATGAGAACAACCACACCTTAGCGAATTGATGCGCCGCTTCGGAATACCGTTTTGGCTACCCGTTACTAAGCCCATCGCGATTTTCAGCTGATCGGTAGGGAGACCGAGAATCTATCGGGCTATGTCACTAAAACTTTCCAAACACCCCGTGTCGATACTGAACGAATCGATGCACACTCCCTTCCTTGAAAACGCACAATCATACAAGTGGGCACATGATGCGTACGCCCATCTAATACATCCAACTCTCTACGCCCTCTTCAAGAGCTGGAAGGGCACCCTGCACTTGGATAAGTGATAACCTCGTAAGGCAAGCTCGTACCGTCATTCATGCGGAAGAGTTAAGACCATTGGAAGTAGGGATAGTTTCGAACCTCGGTTACTAGTCCCAATAAGGGAACCCTGTCTGAAGGATGAGTGT |
| eccMIR618-linearA | TAAGGGCTAAGGTAAAACCCAACATTGAAATATTTGTATTATTAGTAGTAGTGGTAGTATATAAAATGACCCAGGTATGCAATCTTCTTGGAGTTTTTGCTCCTAACACCTTCTTCCCTTCCTCTCCTTTGCCCACCTGCCACAATTCCTCTTGGTTAAACATCACAGTTTTAGTGAGCTATTGTTACTGACGGCAATCCATCAAGTGTATAGGGGAAAGCTAGGTTGAGAATCTCTGGCCTATGTTATCACTATCATCCTTTCCATGGTTAAAATACTGTGAATCTATGAAATGCACCTCAGTTCACTAGGAAATCACTATATTTTAACATTGCTTAAGTTACTGCTATCCTGAGTTTACTTACACCAACTTAACATTATTTTTTTATTGTACTGTGAGTCAGGGACTATAGCAGACACTGAGAATTCAAAGATGAATTTAGAAATGGTCTCTTCCCTCACGGAGTTCTATCCAATAGGGATAGACAAATGCAACAGAGTGTGATAAATGCTTTAATGGGGCCAAAGCAGCAAGTCAATTTAGAGCGCATCAAAGGGTACCAAAGCAGCCTGGGAGAGAGGTGATCTGGAAAATCTCCCAGTAGGTGACCAAAAACTGAGTCCTAACTCTAATTAATTGAATTATAGACTGCTAAAGCTGCACAGTGTTAGAATCATCTAATTCAAACTCCTGATGTGGCACAGAGAGAATCTGATGTCCAGAGAGGCTGATGTATGCAGAGTGAGTATCAGAGTTGCAGCTAGGACCCAGATATGTAATACTGGGTTGCCTCTTTAAAAATGCACCTTTTTTGAAGGGAAATTTTTAGAACCTTTTCCTTCATCAGTTTAATAATGAAAGTAATTAATCATATTTGAATAAGATACGGAGAACAATATTTATATATATTGACTTTATGCTCAGTGGTGATGCCAATAACCCATCATCTCTTGGTTATAGATTTTCCATGAGCTGCTGATGAATTTTTTCAGACTCATCCACAGGGTAACCCTGCTTGTCTCCTGAGCTACTGCATGTACGTAATTACACTCAGAAGGACAAGTAGAGTTTGGCTGTGAACAAGAGCAAACTTTAAAAATAAATCTTAGTCCGATTAGCACCTCCTCTCATCAACCGACTGAGGCATC |
| eccMIR618-linearB | CTGGGAGAGAGGTGATCTGGAAAATCTCCCAGTAGGTGACCAAAAACTGAGTCCTAACTCTAATTAATTGAATTATAGACTGCTAAAGCTGCACAGTGTTAGAATCATCTAATTCAAACTCCTGATGTGGCACAGAGAGAATCTGATGTCCAGAGAGGCTGATGTATGCAGAGTGAGTATCAGAGTTGCAGCTAGGACCCAGATATGTAATACTGGGTTGCCTCTTTAAAAATGCACCTTTTTTGAAGGGAAATTTTTAGAACCTTTTCCTTCATCAGTTTAATAATGAAAGTAATTAATCATATTTGAATAAGATACGGAGAACAATATTTATATATATTGACTTTATGCTCAGTGGTGATGCCAATAACCCATCATCTCTTGGTTATAGATTTTCCATGAGCTGCTGATGAATTTTTTCAGACTCATCCACAGGGTAACCCTGCTTGTCTCCTGAGCTACTGCATGTACGTAATTACACTCAGAAGGACAAGTAGAGTTTGGCTGTGAACAAGAGCAAACTTTAAAAATAAATCTTAGTCCGATTAGCACCTCCTCTCATCAACCGACTGAGGCATCTAAGGGCTAAGGTAAAACCCAACATTGAAATATTTGTATTATTAGTAGTAGTGGTAGTATATAAAATGACCCAGGTATGCAATCTTCTTGGAGTTTTTGCTCCTAACACCTTCTTCCCTTCCTCTCCTTTGCCCACCTGCCACAATTCCTCTTGGTTAAACATCACAGTTTTAGTGAGCTATTGTTACTGACGGCAATCCATCAAGTGTATAGGGGAAAGCTAGGTTGAGAATCTCTGGCCTATGTTATCACTATCATCCTTTCCATGGTTAAAATACTGTGAATCTATGAAATGCACCTCAGTTCACTAGGAAATCACTATATTTTAACATTGCTTAAGTTACTGCTATCCTGAGTTTACTTACACCAACTTAACATTATTTTTTTATTGTACTGTGAGTCAGGGACTATAGCAGACACTGAGAATTCAAAGATGAATTTAGAAATGGTCTCTTCCCTCACGGAGTTCTATCCAATAGGGATAGACAAATGCAACAGAGTGTGATAAATGCTTTAATGGGGCCAAAGCAGCAAGTCAATTTAGAGCGCATCAAAGGGTACCAAAGCAGC |
| eccMIR3661-linearA | AGCCAGCTCACCCTCAAAACCGTCCCTGACGATGACCCACAGGGTCTCCGCTCCCCCTGCCACCTCGTCTGGCGCCAAGGCCGGCTGACTCAAAAGCCCAGGAGTCGAGTGAACGGCGCCATTTTAGGCGGCGGCGGCAACCAATCCCTGCGCGGGAGGCAGGGGGCGCCGGGTCGTTAGGAAGCCGCCGCCGGCCAGGATGGGCGCCGGTCTCGGAGACTCGGGGGGCCCGGACCGGCGGCCTCCGCCATGTTGCACCCTCCTCACGGCCGCCTTTTCCTCGGCGGGCTCGGCCCCGCGGCGGCTGTCCGCAGCCGTACTACAGCTCACCTTCTCGCAGAGGCTCTTGACCTGGGACTCGGACAGCTGCTTGCACTCGTTCAGCTGCTCGATCCACTGGTCCAGCTCCTTGGTGAACACCTTCTCGTCCATGATGCCACCCGCCCCAGCCGGCTGCCCCAACTATGAAAAATAAGTTACACTCCTCCCTTATAGCTACCACCATGACCTTCGTAAAAATTAGGTTAAGAGAATAATTTAAAAATTCTTAAAGACACCCCTTACCTTGATTTCTGGAGAAGATCGCTTGGTGAGTTTAATACAAACCACCAAGGGGTACTAAAGCTGTGTGACCTTTAGGTATGTTCTGGGATACGGCCTAACAAGACTGCCTTTGGGGGATCCTCTGGGCAGCTCTGGACCTAACTAGGCCTGGGTACCAGCGGCAAGACTCTTACTCAGCCCACCGGAGAGGCCCAGGCTCCATTCCCAAGCCGCAGAATCCA |
| eccMIR3661-linearB | TCTGGGATACGGCCTAACAAGACTGCCTTTGGGGGATCCTCTGGGCAGCTCTGGACCTAACTAGGCCTGGGTACCAGCGGCAAGACTCTTACTCAGCCCACCGGAGAGGCCCAGGCTCCATTCCCAAGCCGCAGAATCCAAGCCAGCTCACCCTCAAAACCGTCCCTGACGATGACCCACAGGGTCTCCGCTCCCCCTGCCACCTCGTCTGGCGCCAAGGCCGGCTGACTCAAAAGCCCAGGAGTCGAGTGAACGGCGCCATTTTAGGCGGCGGCGGCAACCAATCCCTGCGCGGGAGGCAGGGGGCGCCGGGTCGTTAGGAAGCCGCCGCCGGCCAGGATGGGCGCCGGTCTCGGAGACTCGGGGGGCCCGGACCGGCGGCCTCCGCCATGTTGCACCCTCCTCACGGCCGCCTTTTCCTCGGCGGGCTCGGCCCCGCGGCGGCTGTCCGCAGCCGTACTACAGCTCACCTTCTCGCAGAGGCTCTTGACCTGGGACTCGGACAGCTGCTTGCACTCGTTCAGCTGCTCGATCCACTGGTCCAGCTCCTTGGTGAACACCTTCTCGTCCATGATGCCACCCGCCCCAGCCGGCTGCCCCAACTATGAAAAATAAGTTACACTCCTCCCTTATAGCTACCACCATGACCTTCGTAAAAATTAGGTTAAGAGAATAATTTAAAAATTCTTAAAGACACCCCTTACCTTGATTTCTGGAGAAGATCGCTTGGTGAGTTTAATACAAACCACCAAGGGGTACTAAAGCTGTGTGACCTTTAGGTATGT |
| eccMIR2277-linearA | GCCTGCTTGCATCAATAGGTGTTATAATACATAACATCTAGATATTATTATATTTTTACTGATTTATTCAAATGACAATCTTAAAGGATTCTTTTGAATTTGGAGGCATTTATATTTTCCAAACCTAACTGAGCAGTTCTACATATGCTCAGACATACCTGCTTTAAATGATATTTGCGGGTAGATCTAGGAGGACCAGTTTCTGGATAGGGTTATCAAATGTGTGTCTCTATTCTATAGTGTTTGCAACCCTAGTTTCACTTCAGTAATAACTACACAGTTGCACTTATAATGGTAGGACATTTTAAAATCAATTGTAATTCATTTTGAAAGACCATTCTCAGATGCATACTACGTGGCAACACCCACCCCCAAATACAATAAGCCAAAAAAAAAAAAAAAGGTGTTAATTGTCTCTTTTCGTAACCATTTCCCAAGCTTACAGCCACATTTGGCGGCAATTAGAGCTTCGCCGGCCGAGCCAGGCAGGGCGCTGTCAGCCTTAATGTGAGCGCTGACTGGCAGCGCTCAGCCCGCGCTCAGCCCGCAGGAAGCACGACCAGTGAACCCATATTGCTTTGACAGTTGCACTCATCTAGAAATAATGCAAAACGCTATTTAGATGTATATATCACGACCCTGGGCTCTGGGAAGAAAACAATCTAATGAGGGGCTGGGAGTCCGCTCGACACAAGCAGTGAGGAGGCGTGCGGGTGGGTGCGCCAGGGCGGCCTCCCCCGTCGCGCTCGCTCCTCTTACAGCTCTTCGTGCTTGATGCGGTGGGAGCGGCGCGTCACAGCCGGCTTCAGGGAGCTGGTCTTGGCCTCTGAGGCTTCGGTAAAGAATTTGAAAATAGACGGAAAGCTCTTCCAGGAAGTTAGCTCGTGACGGTCGGTGCCTGCAAAGAACAACACATTCGGTTACCAGGTGGGCCTGCTTGCATCAATAGGTGTTATAATACATAACATCTAGATATTATTATATTTTTACTGATTTATTCAAATGACAATCTTAAAGGATTCTTTTGAATTTGGAGGCATTTATATTTTCCAAACCTAACTGAGCAGTTCTACATATGCTCAGACATACCTGCTTTAAATGATATTTGCGGGTAGATCTAGGAGGACCAGTTTCTGGATAGGGTTATCAAATGTGTGTCTCTATTCTATAGTGTTTGCAACCCTAGTTTCACTTCAGTAATAACTACACAGTTGCACTTATAATGGTAGGACATTTTAAAATCAATTGTAATTCATTTTGAAAGACCATTCTCAGATGCATACTACGTGGCAACACCCACCCCCAAATACAATAAGCCAAAAAAAAAAAAAAAGGTGTTAATTGTCTCTTTTCGTAACCATTTCCCAAGCTTACAGCCACATTTGGCGGCAATTAGAGCTTCGCCGGCCGAGCCAGGCAGGGCGCTGTCAGCCTTAATGTGAGCGCTGACTGGCAGCGCTCAGCCCGCGCTCAGCCCGCAGGAAGCACGACCAGTGAACCCATATTGCTTTGACAGTTGCACTCATCTAGAAATAATGCAAAACGCTATTTAGATGTATATATCACGACCCTGGGCTCTGGGAAGAAAACAATCTAATGAGGGGCTGGGAGTCCGCTCGACACAAGCAGTGAGGAGGCGTGCGGGTGGGTGCGCCAGGGCGGCCTCCCCCGTCGCGCTCGCTCCTCTTACAGCTCTTCGTGCTTGATGCGGTGGGAGCGGCGCGTCACAGCCGGCTTCAGGGAGCTGGTCTTGGCCTCTGAGGCTTCGGTAAAGAATTTGAAAATAGACGGAAAGCTCTTCCAGGAAGTTAGCTCGTGACGGTCGGTGCCTGCAAAGAACAACACATTCGGTTACCAGGTGG |
| eccMIR2277-linearB | ACATTTGGCGGCAATTAGAGCTTCGCCGGCCGAGCCAGGCAGGGCGCTGTCAGCCTTAATGTGAGCGCTGACTGGCAGCGCTCAGCCCGCGCTCAGCCCGCAGGAAGCACGACCAGTGAACCCATATTGCTTTGACAGTTGCACTCATCTAGAAATAATGCAAAACGCTATTTAGATGTATATATCACGACCCTGGGCTCTGGGAAGAAAACAATCTAATGAGGGGCTGGGAGTCCGCTCGACACAAGCAGTGAGGAGGCGTGCGGGTGGGTGCGCCAGGGCGGCCTCCCCCGTCGCGCTCGCTCCTCTTACAGCTCTTCGTGCTTGATGCGGTGGGAGCGGCGCGTCACAGCCGGCTTCAGGGAGCTGGTCTTGGCCTCTGAGGCTTCGGTAAAGAATTTGAAAATAGACGGAAAGCTCTTCCAGGAAGTTAGCTCGTGACGGTCGGTGCCTGCAAAGAACAACACATTCGGTTACCAGGTGGGCCTGCTTGCATCAATAGGTGTTATAATACATAACATCTAGATATTATTATATTTTTACTGATTTATTCAAATGACAATCTTAAAGGATTCTTTTGAATTTGGAGGCATTTATATTTTCCAAACCTAACTGAGCAGTTCTACATATGCTCAGACATACCTGCTTTAAATGATATTTGCGGGTAGATCTAGGAGGACCAGTTTCTGGATAGGGTTATCAAATGTGTGTCTCTATTCTATAGTGTTTGCAACCCTAGTTTCACTTCAGTAATAACTACACAGTTGCACTTATAATGGTAGGACATTTTAAAATCAATTGTAATTCATTTTGAAAGACCATTCTCAGATGCATACTACGTGGCAACACCCACCCCCAAATACAATAAGCCAAAAAAAAAAAAAAAGGTGTTAATTGTCTCTTTTCGTAACCATTTCCCAAGCTTACAGCC |
|  |  |
| **Reverse transcription PCR Primers for miRNAs** | **5' - 3'** |
| MIR3661-RT | GTCGTATCCAGTGCAGGGTCCGAGGTATTCGCACTGGATACGACCAGCTG |
| MIR618-RT | GTCGTATCCAGTGCAGGGTCCGAGGTATTCGCACTGGATACGACACTCAG |
| MIR2277-RT | GTCGTATCCAGTGCAGGGTCCGAGGTATTCGCACTGGATACGACGACTGG |
| snRNA U6 RT/q-R | AACGCTTCACGAATTTGCGT |
| **miRNA qPCR Primers** | **5' - 3'** |
| MIR3661-q-F | CGTGACCTGGGACTCGGA |
| MIR3661-q-F | AGTGCAGGGTCCGAGGTATT |
| MIR618-q-F | GCGCGAAACTCTACTTGTCCTT |
| MIR618-q-F | AGTGCAGGGTCCGAGGTATT |
| MIR2277-q-F | CGCGGGCTGAGCGCTG |
| MIR2277-q-F | AGTGCAGGGTCCGAGGTATT |
| **siRNA qPCR Primers** | **5' - 3'** |
| Rad51-q-F1 | CAACCCATTTCACGGTTAGAGC |
| Rad51-q-R1 | TTCTTTGGCGCATAGGCAACA |
| Xrcc5-q-F1 | GTGCGGTCGGGGAATAAGG |
| Xrcc5-q-R1 | GGGGATTCTATACCAGGAATGGA |
| Lig3-q-F1 | TCACTGGCGTGATGTAAGACA |
| Lig3-q-R1 | CCTGGAATGATAGAACAGGCTTT |
| POLQ-q-F | GTGAAGACCCGTTTACCTTAGA |
| POLQ-q-R | AGATCCTGTGACAATATGCTCC |
| KU80-q-F | CGTGGCTTTTCCTCATATCAAG |
| KU80-q-R | GAAACAAGTCTTCAAGGGTGTC |
| DNA-PKCS-q-F | TTCTCAAGGAAGAAGGTGTCTC |
| DNA-PKCS-q-R | CAATGAACGTGTTGTAGCACTC |
| **siRNA Sequence** | **5' - 3'** |
| H3980-siLIG3-1 |  |
| sense | CAAGUACGAUGGAGAGCGA(dT)(dT) |
| antisense | UCGCUCUCCAUCGUACUUG(dT)(dT) |
| H3980-siLIG3-2 |  |
| sense | GCGGCAUGAUGUCAAUCUU(dT)(dT) |
| antisense | AAGAUUGACAUCAUGCCGC(dT)(dT) |
| H3980-siLIG3-3 |  |
| sense | CACGGUGAUGUGUACCUAA(dT)(dT) |
| antisense | UUAGGUACACAUCACCGUG(dT)(dT) |
| H10721-siPOLQ-1 |  |
| sense | AUAGUAGCUUGUCUCUUUC(dT)(dT) |
| antisense | GAAAGAGACAAGCUACUAU(dT)(dT) |
| H10721-siPOLQ-2 |  |
| sense | GAUUUGUGAUAACCAUUCA(dT)(dT) |
| antisense | UGAAUGGUUAUCACAAAUC(dT)(dT) |
| H10721-siPOLQ-3 |  |
| sense | GGCAGCACCUCUCCAUCAA(dT)(dT) |
| antisense | UUGAUGGAGAGGUGCUGCC(dT)(dT) |
| H7520-siKu80-1 |  |
| sense | GCGAGUAACCAGCUCAUAA(dT)(dT) |
| antisense | UUAUGAGCUGGUUACUCGC(dT)(dT) |
| H7520-siKu80-2 |  |
| sense | GCAUGGAUGUGAUUCAACA(dT)(dT) |
| antisense | UGUUGAAUCACAUCCAUGC(dT)(dT) |
| H7520-siKu80-3 |  |
| sense | CCUCAUAUCAAGCAUAACUAU(dT)(dT) |
| antisense | AUAGUUAUGCUUGAUAUGAGG(dT)(dT) |
| H5591-siDNA-PKCS-1 |  |
| sense | CAUUGAACUCGAUGUUAGA(dT)(dT) |
| antisense | UCUAACAUCGAGUUCAAUG(dT)(dT) |
| H5591-siDNA-PKCS-2 |  |
| sense | AUCUAUACAGCUUAGCAAG(dT)(dT) |
| antisense | CUUGCUAAGCUGUAUAGAU(dT)(dT) |
| H5591-siDNA-PKCS-3 |  |
| sense | AAUUGUAGUCGCAAGUUUC(dT)(dT) |
| antisense | GAAACUUGCGACUACAAUU(dT)(dT) |
| H5888-siRAD51-1 |  |
| sense | GCAGUGAUGUCCUGGAUAA(dT)(dT) |
| antisense | UUAUCCAGGACAUCACUGC(dT)(dT) |
| H5888-siRAD51-2 |  |
| sense | CGAUGUGAAGAAAUUGGAA(dT)(dT) |
| antisense | UUCCAAUUUCUUCACAUCG(dT)(dT) |
| H5888-siRAD51-3 |  |
| sense | GACUGGAUCUAUCACAGAA(dT)(dT) |
| antisense | UUCUGUGAUAGAUCCAGUC(dT)(dT) |
| Negative control |  |
| sense | UUCUCCGAACGUGUCACGU(dT)(dT) |
| antisense | ACGUGACACGUUCGGAGA4(dT)(dT) |
