# Supplementary material for: Extrachromosomal circular DNA expressing miRNA promotes ovarian cancer progression
Source: Clin Transl Med. 2025 Sep 23;15(9):e70445. doi: 10.1002/ctm2.70445 (PMC12455017; doi:10.1002/ctm2.70445)
Supplement: Supplementary file 10 — Supporting Information [file CTM2-15-e70445-s009.docx]

**Table S5 pmirGLO**

|  | **miRNA sequence (5'-3')** | **UTR sequence (5'-3')** | **UTR sequence (R&C)** | **oligo 5'-linker** | **oligo linker-3'** | **UTR-oligo-F** | **UTR-oligo-R** |
| --- | --- | --- | --- | --- | --- | --- | --- |
| hsa-mir-3661 | UGACCUGGGACUCGGACAGCUG | CAGCTGTCCGAGTCCCAGGTCA | TGACCTGGGACTCGGACAGCTG | TCGA | GGCC | TCGACAGCTGTCCGAGTCCCAGGTCA | GGCCTGACCTGGGACTCGGACAGCTG |
| hsa-mir-618 | AAACUCUACUUGUCCUUCUGAGU | ACTCAGAAGGACAAGTAGAGTTT | AAACTCTACTTGTCCTTCTGAGT | TCGA | GGCC | TCGAACTCAGAAGGACAAGTAGAGTTT | GGCCAAACTCTACTTGTCCTTCTGAGT |
| hsa-mir-2277 (3p) | UGACAGCGCCCUGCCUGGCUC | GAGCCAGGCAGGGCGCTGTCA | TGACAGCGCCCTGCCTGGCTC | tTCGA | GGCC | TCGAGAGCCAGGCAGGGCGCTGTCA | GGCCTGACAGCGCCCTGCCTGGCTC |
